# Understanding the Combined Effects of High Glucose Induced Hyper-Osmotic Stress and Oxygen Tension in the Progression of Tumourigenesis: From Mechanism to Anti-Cancer Therapeutics

**DOI:** 10.3390/cells12060825

**Published:** 2023-03-07

**Authors:** Gayathri K. G., Puja Laxmanrao Shinde, Sebastian John, Sivakumar K. C., Rashmi Mishra

**Affiliations:** 1 Laboratory of Translational Mechanobiology, Rajiv Gandhi Centre for Biotechnology, Thiruvananthapuram 695014, Kerala, India; 2 Manipal Academy of Higher Education, Manipal 576104, Karnataka, India; 3 Distributed Information Sub-Centre (Bioinformatics Centre), Bio-Innovation Center (BIC), Rajiv Gandhi Centre for Biotechnology, Thiruvananthapuram 695014, Kerala, India

**Keywords:** mechanical force, high-glucose hyper-osmotic stress, SUMO2, pH3(Ser10), tumour proliferation, natural product therapy

## Abstract

High glucose (HG), a hallmark of the tumour microenvironment, is also a biomechanical stressor, as it exerts hyper-osmotic stress (HG-HO), but not much is known regarding how tumour cells mechanoadapt to HG-HO. Therefore, this study aimed to delineate the novel molecular mechanisms by which tumour cells mechanoadapt to HG/HG-HO and whether phytochemical-based interference in these mechanisms can generate tumour-cell-selective vulnerability to cell death. Mannitol and L-glucose were used as hyper-osmotic equivalents of high glucose. The results revealed that the tumour cells can efficiently mechanoadapt to HG-HO only in the normoxic microenvironment. Under normoxic HG/HG-HO stress, tumour cells polySUMOylate a higher pool of mitotic driver pH3(Ser10), which translocates to the nucleus and promotes faster cell divisions. On the contrary, acute hypoxia dampens HG/HG-HO-associated excessive proliferation by upregulating sentrin protease SENP7. SENP7 promotes abnormal SUMOylation of pH3(Ser10), thereby restricting its nuclear entry and promoting the M-phase arrest and cell loss. However, the hypoxia-arrested cells that managed to survive showed relapse upon reversal to normoxia as well as upregulation of pro-survival-associated SENP1, and players in tumour growth signalling, autophagy, glycolytic pathways etc. Depletion of SENP1 in both normoxia and hypoxia caused significant loss of tumour cells vs undepleted controls. SENP1 was ascertained to restrict the abnormal SUMOylation of pH3(Ser10) in both normoxia and hypoxia, although not so efficiently in hypoxia, due to the opposing activity of SENP7. Co-treatment with Momordin Ic (MC), a natural SENP1 inhibitor, and Gallic Acid (GA), an inhibitor of identified major pro-tumourigenic signalling (both enriched in *Momordica charantia*), eliminated surviving tumour cells in normal glucose, HG and HG-HO normoxic and hypoxic microenvironments, suggesting that appropriate and enhanced polySUMOylation of pH3(Ser10) in response to HG/HG-HO stress was attenuated by this treatment along with further dampening of other key tumourigenic signalling, due to which tumour cells could no longer proliferate and grow.

## 1. Introduction

Global Cancer Statistics (GLOBOCAN, 2020) shows an alarming rise in the cancer burden [1]. The primary reasons for treatment failure are due to rapidly expanding tumour heterogeneity and recurrence, chemo- and radio-resistance, oncogenic mutations, alternative splice variants, gene polymorphisms and rapid chromatin remodelling, all of which have posed enormous difficulties and challenges in identifying the specific cellular targets for rational anti-tumour therapeutics and prophylactic vaccine design [2,3,4,5]. Therefore, deeper insights and newer arenas that expose tumour cell vulnerabilities need to be rapidly explored to discover tumour-selective drugs with high therapeutic efficacy, sustainable costs and successful clinical outcomes.

Tumour cells display robust adaptation to the microenvironment (TME)-associated biomechanical challenges. Therefore, therapeutic targeting of TME-associated mechanoadaptation is emerging as a ‘hot spot’ of tumour cell vulnerability and a promising avenue in cancer eradication [6,7,8,9,10,11,12,13,14,15,16,17,18,19,20]. High glucose (HG) is not only a hallmark of cancer but also acts as a hyper-osmotic stressor in TME. Therefore, the major objectives of this study were to excavate the mechanisms by which tumour cells osmo-adapt to high glucose associated hyper-osmotic stress in normoxic and hypoxic microenvironments and how this adaptation can be disabled.

Irrespective of diabetes, hyperglycaemia is a prominent causative factor for cancer cell proliferation and promotes poor patient survival. Hyperglycaemia is often encountered in cancer patients due to several factors such as high carbohydrate uptake, altered metabolism, hormonal disorders, chronic stress, obesity, anti-cancer drugs’ side effects, etc. [21,22]. The blood glucose level in a healthy adult is between 90 to 100 mg/dL, and concentrations higher than 200 mg/dL (11.1 mM) are hyperglycaemic. Glioblastoma patients have been evidenced to have as high as 459 mg/dL (approx. 25.5 mM) of blood glucose levels, and hyperglycaemic concentrations are equally well correlated with cervical cancer progression in patients [23,24]. Although there are also glucose-restricted zones in tumours with 0.45 g/L (2.5 mM) of glucose, PET scan studies have confirmed the existence of hyperglycaemia in tumours vs other body organs, probably due to interstitial fluid flow [24]. In addition, NOD (non-obese diabetic) mice, which are the chosen model for tumour xenograft studies, show hyperglycaemic glucose levels between 10 to 30 mM, which is why tumour progression is enabled, confirming the crucial role of hyperglycaemia in driving tumourigenesis [25]. Furthermore, this is the base reason that tumour cells are standardly grown and passaged in 4.5 g/L (25 mM) glucose-containing medium, and tumour cell studies are performed in media containing 10–30 mM of glucose.

There are few reports that cancer cells rapidly adjust to high-glucose-mediated hyper-osmotic (HG-HO) stress by adjusting their shape and volume, which influence the net surface tension and enable cell survival [26,27]. In addition, the rounding of cells is reported to signal cells to enter the cell cycle and divide [28,29,30,31]. More alarmingly, the emergent phenotypes in osmotic stress conditions are associated with increased drug resistance, providing additional survival advantages to tumourous cells [27]. Therefore, given the timely relevance, we explored the contemporary area of mechano-oncology for excavating the mechanisms associated with tumour cell mechanical adaptations to hyperglycaemia-associated hyper-osmotic stress (HG-HO) under high and low oxygen tension.

Effects of the osmotic components of glucose can be dissected from its metabolic component and can be investigated by using the glucose osmotic mimics mannitol and l-glucose [32,33]. Hence, the treatment protocols to study HG/HG-HO-mediated molecular changes were performed in the presence of mannitol and l-glucose as independent controls for the media osmolality. Appropriate volumes of mannitol (osmo-mimic for d-glucose, not uptaken, not metabolized) or l-glucose (enantiomer of d-glucose, not metabolized) were added to the physiological or normal glucose baseline of 5.5 mM (NG), to obtain the equivalent osmolality as in the HG condition. Similar changes observed in HG and the HG-HO can be attributed to the function of the osmotic component of HG rather than its signalling component. However, it is essential not to take mannitol/l-glucose directly (bypassing baseline 5.5 mM glucose levels), because this can disturb the glucose-mediated signalling in normal cells and may generate artefacts [33].

The general experimental set-up is explained in Supplementary Figure S1. We escalated the major findings for validation in various human tumour cell lines. We sought convincing evidence of the same in the relevant clinical samples, which is imperative for bench-to-bedside translation. We further investigated whether phytochemical-base perturbation of crucial mechanisms in HG osmoregulation can promote the uptake of chemotherapies and attenuate tumour growth.

## 2. Materials and Methods

Please note that additional details on reagents and standard protocols are available in the supporting information file accompanying this article.

### 2.1. Cell Lines, Reagents

Human cervical cancer cell lines CaSki (HPV16+ve and HPV18+ve) and C-33A (HPV-ve); metastatic colon cancer cell line HCT116; metastatic pancreatic cancer cell line MIAPaCa-2; metastatic breast cancer cell line MDA-MB-231 and human normal keratinocytes cell line HaCaT were obtained from and authenticated by American Tissue Culture Collection, Manassas, VA, USA. SVG, the immortalized human astrocyte cell line, was a gift from Prof. Pankaj Seth, National Brain Research Centre, Manesar, India and was initially procured from Prof. Eugene O Major, NINDS/NIH, Bathesda, USA. H9C2, rat cardiomyoblasts, were procured from the cell line repository of the National Centre for Cell Science (NCCS), Pune, India. Cancer cells were maintained in DMEM (Gibco-12100-046) with 10% Foetal Bovine Serum (Himedia—RM9955) and 1x antibiotics (Himedia—A002). Normal cells were cultured as described previously [12,13,34]. All common chemicals were purchased from Sigma-Aldrich, St.Louis, MO unless otherwise indicated. The antibodies used are as follows: pH3(Ser10) [9701S], phospho-p44/42 MAPK (Thr202/Tyr204) [4370]—Cell Signaling Technologies, Danvers, MA, USA; pH3(Ser10) [ab267372], SUMO1(ab32058), SUMO4(ab126606), SUMO2/3 (ab81371)—Abcam, Waltham, MA, USA; SUMO2 (sc26972), HIF1a (sc10790), GAPDH—(sc47724)—Santacruz Biotechnology, Dallas, TX, USA; Cholesterol (abx100311)—Abbexa, Houston, TX, USA; AURKB (A1020), ALDOC (A11618), Phospho p70S6K (T389) [AP1059]—Abclonal, Woburn, MA, USA.

### 2.2. Cell Culture Treatments with Normal, High-Glucose and Hyper-Osmotic Equivalents in Normoxia and Hypoxia Conditions

**Figure 1:**
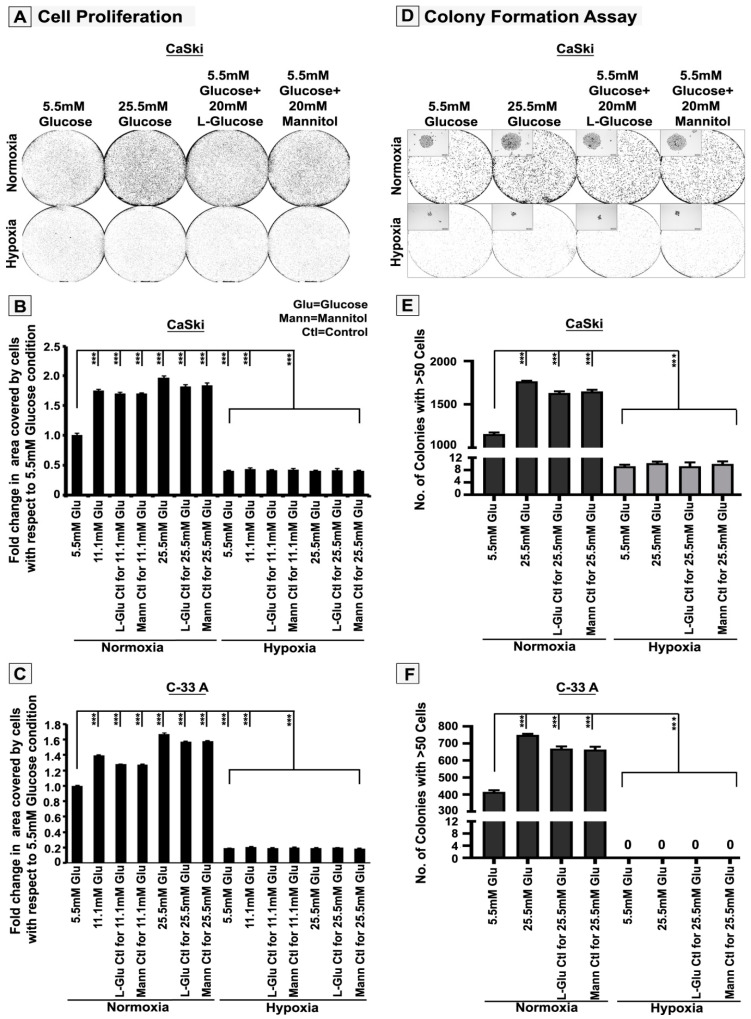
**Tumour cells show efficient adaptation to the hyper-osmotic stress generating property of high glucose and significantly proliferate in normoxia.** CaSki/C-33A cervical cancer cells were cultured for 96 h in NG, HG and HG-HO, with or without hypoxia stimulation. (**A**–**C**) Tumour cell proliferation, (**D**–**F**) Colony-forming analyses were performed, and all comparisons were made with NG condition unless otherwise indicated. Statistical significance is represented by *p* values: *** *p* ≤ 0.001. See related Supplementary Figure S3A.

**Figure 2:**
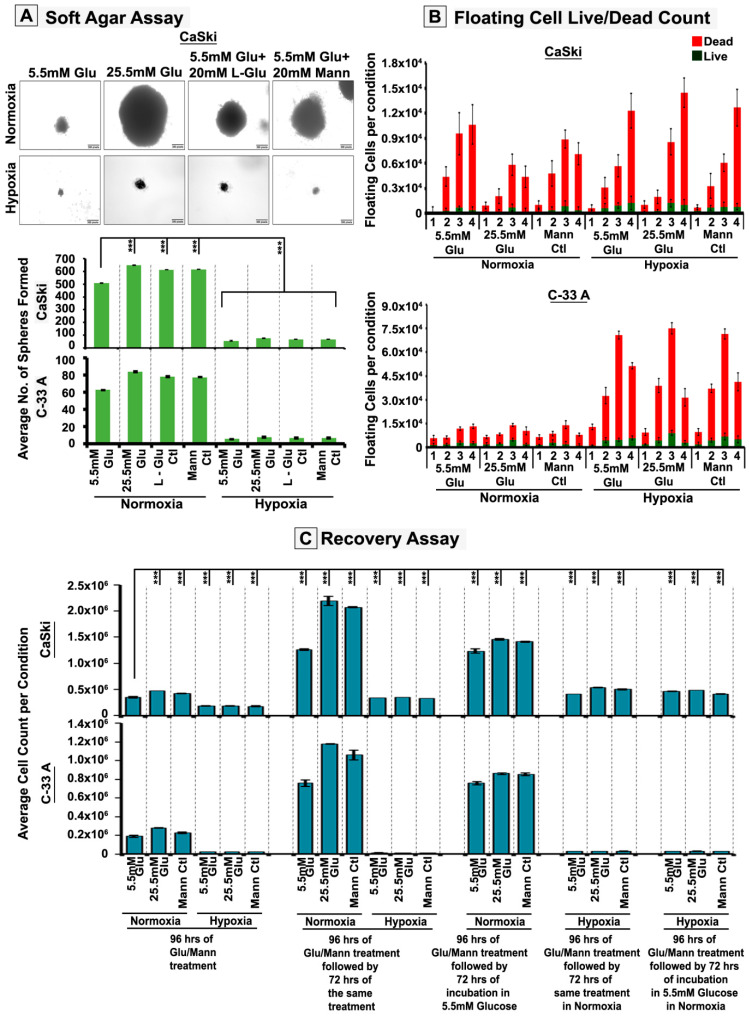
**Acute hypoxia attenuates high glucose hyper-osmotic stress pro-proliferative mechanoadaptive effects and generates dormant tumour cell phenotypes, which can recover upon intercepting normoxia.** (**A**) CaSki/C-33A cervical cancer cells were subjected to soft agar-based anchorage-independent NG, HG and HG-HO conditions, with or without hypoxia stimulation. The number of spheroids formed showed substrate-independent self-renewable capacity of a single cell. (**B**) CaSki/C-33A cells showed many dead floating cells in NG, HG and HG-HO hypoxic conditions. (**C**) CaSki/C-33A cervical cancer cells were cultured as described for panel (**A**). (**i**) The normoxic HG and HG-HO stress conditions were then subjected to NG for the next 72 h, and cell proliferation was determined to estimate the effect of glucose normalization on tumour growth. (**ii**) Tumour cells, initially grown in NG, HG and HG-HO hypoxic conditions were subsequently cultured in normoxia for the next 72 h. (**iii**) Hypoxia-grown cells were reversed to NG normoxic conditions; cell proliferation was determined in (**ii**) and (**iii**) to estimate the renewability/relapse of residual growth in hypoxia upon intercepting conducive normoxic NG, HG and HG-HO conditions. Statistical significance is represented by *p* values: *** *p* ≤ 0.001.See related Supplementary Figure S3B,C.

The treatments were conducted by culturing the cells either under normoxia or chemically induced hypoxia by the addition of 150 µM of CoCl_2_ [35]. In both conditions, the effects of normal as well as high glucose (HG) concentrations and the contribution of its corresponding osmotic component (HG-HO) to the observed effects were studied by treating the cells with either 20 mM of glucose as the hyperglycaemic condition or 20 mM of mannitol as the osmotic control for the hyperglycaemic condition, while maintaining 5.5 mM of glucose as basal level (NG) in all the conditions [32,33]. In the figures, normal glucose level, high glucose level and mannitol osmotic control are designated as Nor Glu, High Glu and Mann Ctl, respectively, except in Figures 1 and 2. Figures 1 and 2 have data on various concentrations of high glucose and two osmotic controls; therefore, normal glucose level, high glucose levels, l-glucose osmotic controls and mannitol osmotic controls are designated as 5.5 mM Glu, 11.5/25.5 mM High Glu, 11.5/25.5 mM L-Glu Ctl and 11.5/25.5 mM Mann Ctl, respectively. In the text, 5.5 mM normal glucose level is abbreviated as NG, high glucose as HG and high-glucose hyper-osmotic stress as HG-HO.

### 2.3. Confluence Analysis

Cells were cultured in 6-well plates with an initial seeding density of 15,000 cells/well. After exposing cells to the basic experimental set-up treatments, the cells were fixed with 1.5% PFA for 20 min at RT. The wells were imaged at 4x magnification using the TECAN Multimode reader/imager. The whole well images were thresholded using the Otsu tool in Fiji image analysis software (FIJI is just ImageJ 2.9.0/1.53t; Java 1.8.0_322; 64-bit) [36],and the number of cells was counted by running the “Analyze particles” option.

### 2.4. Floating Cell Count with Trypan Blue

The cells were seeded in 100 mm cell culture dishes and were treated with high glucose/mannitol in conjunction with either normoxia or hypoxia for 96 h. The medium was collected daily, and the floating cells were pelleted down by centrifuging the media at 2000 rpm for 5 min. The floating cell pellet was re-suspended in 30 µL medium, and equal volumes of this cell suspension were mixed with trypan blue solution. Cell count was scored manually using a hemocytometer, and dead and live cell counts per quadrant were registered.

### 2.5. Proximity Ligation Assay (PLA)

A Duolink in situ PLA kit from Sigma-Aldrich, St. Louis, MO, USA was used to investigate the interaction between SUMO2 and pH3(Ser10), according to the manufacturer’s instructions. Briefly, the cells were fixed with 1.5% PFA for 20 min at RT after the treatments. Cell permeabilization was conducted with 0.25% saponin in PBS. One drop of blocking solution per 1 cm^2^ area was added, and the slides were incubated in a pre-heated humidified chamber at 37 °C for 30 min. Respective primary antibodies were diluted to recommended concentrations in the antibody diluent and were added to the cells, and the slides were incubated overnight at 4 °C in a humidified chamber. Post incubation, the slides were washed with 1x wash buffer A, and the plus and minus PLA probes, diluted in 1:5 in antibody diluent, were added to the cells. After incubating the cells for 1 h at 37 °C with the PLA probe solution, the slides were washed with 1x wash buffer A. The slides were then incubated with 40 µL of ligation mix per well in a humidified chamber for one hour at 37 °C. After washing with 1× wash buffer A, the required volume of signal amplification mix with polymerase enzyme was added to the cells. It was incubated for one hundred minutes at 37 °C in a humidified chamber. Final washes were given with wash buffer A and wash buffer B, and the cells were stained for Hoechst for six minutes at RT, followed by mounting in 70% glycerol.

### 2.6. Immunohistochemistry on Tissue Microarrays

Uterine cervical tumour tissue array (US Biomax, Derwood, MD, USA, cat no. CR2087): uterine cervical carcinoma tissue microarray containing 104 cases of malignant tumour (87 squamous cell carcinoma, 3 adeno squamous carcinomas and 14 adenocarcinomas) with duplicate cores were used for IHC. The multiple tumour tissue array (cat nos. T6235700-1) was from BioChain Institute Inc., Newark, CA, USA. Antigen retrieval was performed on TMA slides by boiling the tissues in basic heat-induced epitope recovery citrate buffer (0.01 M Sodium Citrate buffer, pH6). The TMA slides were then permeabilized with 0.01% digitonin for 30 min at RT and blocked for 1 h in the blocking mixture, constituting 5% BSA and 2% donkey serum. TMA slides were incubated with primary antibodies overnight at 4 °C. Fluorophore-labelled secondary antibodies were incubated for 1.5 h at RT. Slides were mounted in 70% glycerol in PBS after Hoechst staining for 5 min at RT. The stained sections were visualized and imaged using a confocal microscope and were analysed with Fiji Image analysis software. 

### 2.7. FRET Analysis

Antibody-mediated FRET assay was performed to confirm the direct interaction between SUMO2 and pH3(Ser10). Briefly, the cells were fixed after treatment with 1.5% PFA for 20 min at RT. The cells were immunostained with pH3(Ser10) (9701S) and SUMO2 (sc-26972) antibodies. Signal amplification was performed for pH3(Ser10) to obtain strong donor fluorescence intensity by performing TSA-Biotin-mediated fluorescence enhancement. Further, immunostaining of pH3(Ser10) and SUMO2 was developed with donkey anti-mouse Cy3 (Jackson Immuno Research, West Grove, PA, USA; 1:200) and donkey anti-goat IgG conjugated with Alexa 647 (Invitrogen, Waltham, MA, USA; 1:100), respectively. The FRET signal intensities on mitotic chromosomes were obtained by outlining the chromosomes’ periphery in Fiji and subsequent use of the ‘Measure’ tool. The nuclear and cytoplasmic FRET channel (acceptor emission after donor excitation) signals were likewise obtained, and nuclear vs cytoplasmic FRET signal intensities were graphically represented.

### 2.8. Three-Dimensional Pellet Cultures and Drug Treatments

In 96-wellround-bottom plates, 3 × 10^4^ cells were seeded per well in MEM media [12]. The plates were centrifuged in a swinging bucket rotor at 500× *g* for 5 min to generate the 3D cell pellet. The HG, HG-HO and oxygen tension treatments were started post 24 h. After establishing each microenvironmental condition for 96 h, individual and in-combination drug treatments were started, which were continued for up to 9 days to capture the response of individual cell lines to the therapeutics. Momordin Ic (MC) was used at a concentration of 25 µM and Gallic Acid (GA) at 100 µM, as these concentrations did not show toxicity to normal cells in the MTT assay. In addition, these dosages have been validated in cell culture studies, where the experiments were escalated to in vivo tumour models [37,38]. The pellet images were captured daily, and the volume analysis was performed using the Reconstruction and Visualization from a Single Projection (ReViSP; https://sourceforge.net/p/revisp, accessed on 21 February 2023) tool, which enables the 3D volume reconstruction by quantitating the voxels (3D pixels) [39,40].

### 2.9. Image Analysis

Confocal images were hyper-stacked to visualize the maximum intensity projections using Fiji software, and the cell outlines were manually drawn. The fluorescence intensity was measured for the individual cells and their respective nuclei. Cytoplasmic fluorescence intensity was obtained by subtracting the integrated fluorescence intensity of the nucleus from the whole cell. Image acquisition parameters for each channel were kept the same across each condition and over independent replicates. Colocalization analysis was performed using the Coloc tool, and the extent of colocalization was expressed as Mander’s coefficient of colocalization (R).

### 2.10. Statistical Analysis

Statistical analyses were performed using one-tailed unpaired Bonferroni’s *t*-test. The following *p* values represented statistical significance: * *p* ≤ 0.05, ** *p* ≤ 0.01 and *** *p* ≤ 0.001. All comparisons were made with the control 5.5 mM normoxic condition unless otherwise indicated. Data are represented as means ± SD and averaged from at least three independent experiments.

## 3. Results

### 3.1. High-Glucose-Induced Hyper-Osmotic Stress Drives Tumour Cell Proliferation under Normoxia

In order to delineate the mechanisms by which tumour cells osmo-adapt to high-glucose-associated hyper-osmotic (HG-HO) stress in normoxic and hypoxic microenvironments, the experimental set-up was designed, which is explained in Supplementary Figure S1. Acute hypoxia was generated as described previously [35], and elevated hypoxia marker HIF1a was confirmed in 12 and 48 h (Supplementary Figure S2). Tumour cells exposed to high glucose concentrations (HG, 11.5 and 25.5 mM) and the hyper-osmotic equivalents (HG-HO) showed high proliferation of the bulk tumour population in normoxia (Figure 1A–C and Figure S3A). The 2D colony formation and 3D anchorage-independent spheroid-based growth assays (soft agar assay) that determine the capacities of cells to self-renew also showed that individual tumour cells could efficiently grow in HG-HO condition and thereby adapt to this stress, but only in normoxia (Figure 1D–F, Figure 2A and Figure S3B,C). Overall, these assays suggest that tumour cells can over-proliferate not only in response to better growth conditions provided by HG (5.6–20 mM extra glucose) but also due to HG-HO stress, which has only baseline glucose levels of 5.5 mM. Indeed, proliferation-associated mitotic rounding has been reported to enable osmo-adaptive membrane tension and, thereby, could be a clever mechanoadaptive strategy to survive in adverse conditions that present osmotic challenges [26,27,28,29,30,31].

### 3.2. Hypoxia Retards High-Glucose Hyper-Osmotic-Stress-Induced Tumour Cell Proliferation by Triggering G2/M Cell Cycle Arrest

We observed significantly less survival under acute hypoxia irrespective of the presence of NG, HG or HG-HO conditions. This suggests that acute hypoxia attenuated HG/HG-HO osmo-adaptive mechanisms. Therefore, delineating the identity of hypoxia-induced ‘anti-mechanoadaptive’ molecular mechanisms was particularly interesting at this stage. These were clues to strategies for transforming tumour cells vulnerable to death. Therefore, we delve deeper into the cell fate and molecular analysis of acute hypoxia-induced compromised survival. Under hypoxia, a significant number of floating cell populations were observed that were mostly dead. When this floating cell population was replated under normoxic conditions, a few cells revived and formed colonies, signifying the dangerous capacity to relapse upon the return of conducive conditions (Figure 2B). However, the acute hypoxia-induced cell death and the residual adherent cell growth were not due to senescence (Supplementary Figure S4A). Instead, such phenotypes expressed markers of stemness (Supplementary Figure S4B,C) and were able to reverse growth retardation upon exposure to normoxia (Figure 2C). In addition, the floating cell count, although significant, could not wholly account for the dramatic loss in tumour cell numbers observed in acute hypoxia.

**Figure 3:**
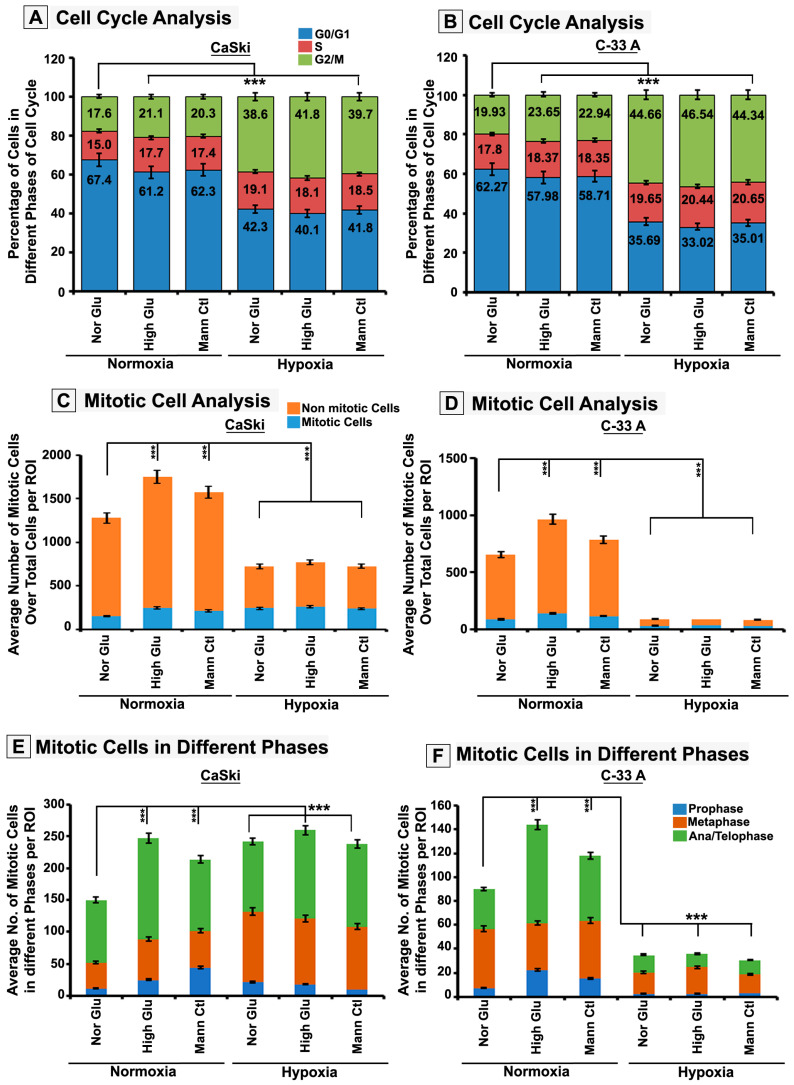
**Acute hypoxia attenuates high-glucose hyper-osmotic stress pro-proliferative mechanoadaptive effects by promoting mitotic M-phase arrest.** CaSki/C-33A cervical cancer cells were cultured for 96 h in NG, HG and HG-HO with or without hypoxia stimulation. (**A**,**B**) FACS-assisted cell cycle analysis was performed. (**C**,**D**) Average mitotic cell counts over the total cell number per ROI were obtained to estimate whether more cells were stalled in mitosis over total cells in each condition. (**E**,**F**) The images of mitotic cells per ROI were further scored into different mitotic (pro, meta and ana/telo) phases manually to estimate whether the distribution of mitotic cells in these phases could correlate with total cell numbers or not. Statistical significance is represented by *p* values: *** *p* ≤ 0.001.

Since cell proliferation is regulated by the cell cycle, flow-cytometry-assisted cell cycle analysis was performed, which revealed robust G2/M arrest in acute hypoxia (Figure 3A). Further, a higher mitotic cell count over the total number of cells was registered in hypoxic conditions, but this did not corroborate with the expected rise in proliferation (data shown in Figure 1 and Figure 2), which again suggests that acute hypoxia induces M-phase arrest (Figure 3B). Furthermore, the residual growth in hypoxia showed the formation of micronuclei, an indicator of abnormal cell cycle and mitotic chromosome segregation, which again points to the M-phase defects (Supplementary Figure S4D) [41]. A deeper analysis of the M-phase distribution of tumour cells revealed that cells in hypoxia were stuck in pro-metaphase and late anaphase/telophase stages (Figure 3C–F).

**Figure 4:**
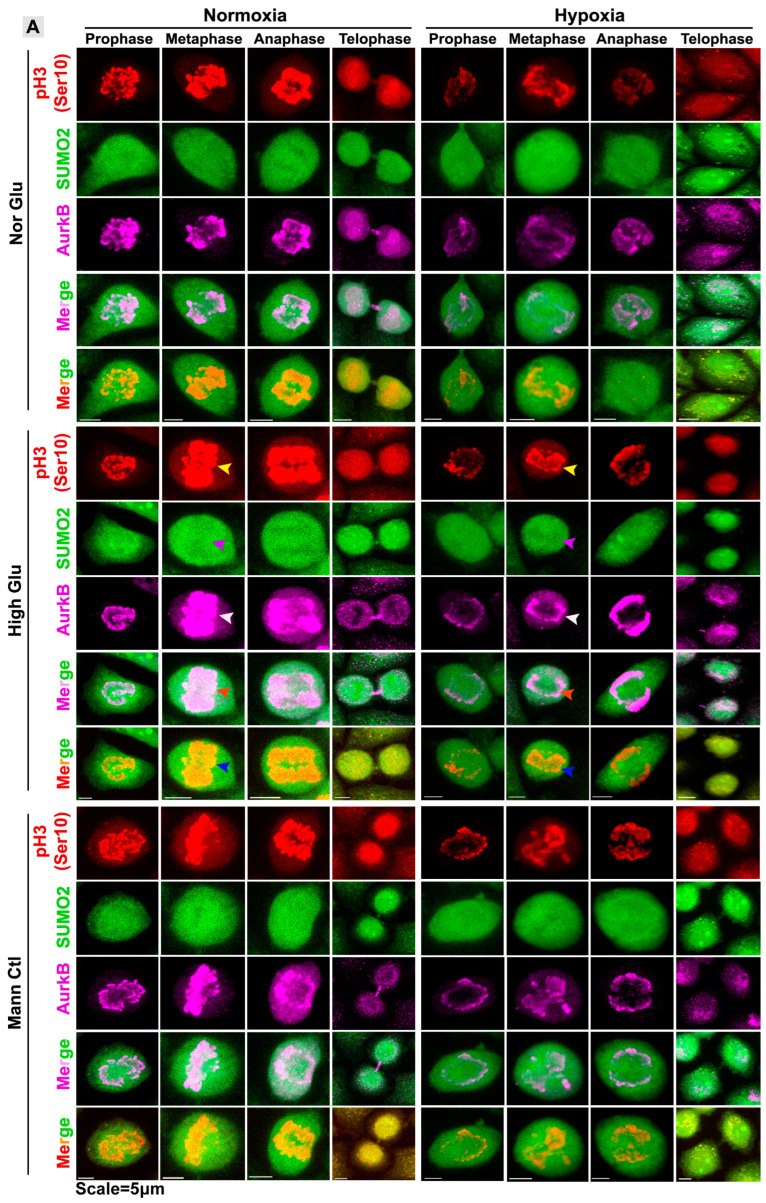

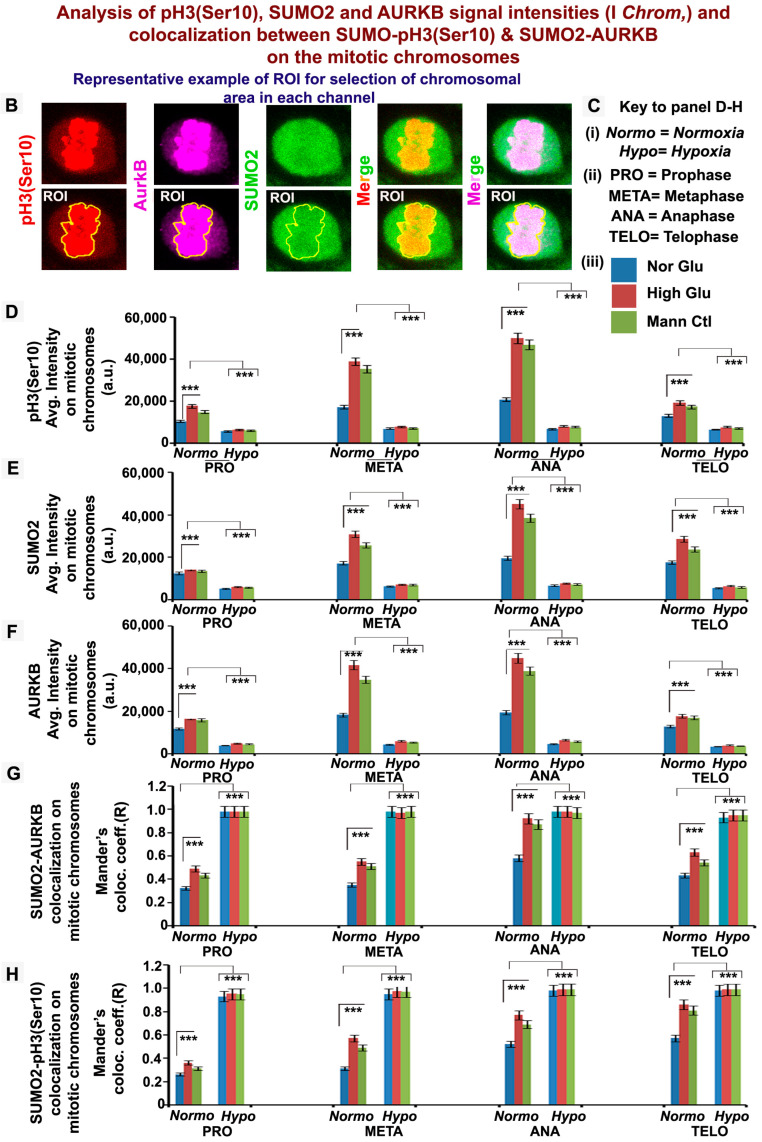
**Acute hypoxia restricts the localization of SUMO2, AURKB and pH3(Ser10) on the mitotic chromosomes of the tumour cells.** CaSki/C-33A cervical cancer cells were cultured for 96 h in NG, HG and HG-HO, with or without hypoxia stimulation. (**A**) Cells were immunostained with SUMO2, AURKB and pH3(Ser10). Coloured arrows in the middle panel indicate an example of differences in expression between normoxia and hypoxia. (**B**) An example of free-hand ROI selection for analysis of protein intensities and colocalization. (**C**–**F**) The fluorescence intensity (I) of each protein on mitotic chromosomes is indicated in the panel. (**G**,**H**) The extent of colocalization of SUMO2 with AURKB and pH3(Ser10) is expressed as Mander’s coefficient of colocalization (R). Statistical significance is represented by *p* values: *** *p* ≤ 0.001. In addition, see related Supplementary Figures S5 and S6.

Several kinases orchestrate the transition from G2 to the M phase. However, AURKB, predominantly involved in the exit from prophase and transition into metaphase–anaphase, has significant roles in the process of cytokinesis [42]. Most of the molecular players involved in M-phase progression, including AURKB, have been identified as substrates of SUMOylation, especially by the SUMO2 isoform [43,44] (Supplementary Table S1). Therefore, we examined the SUMO2-modified AURKB on mitotic cells, which was identified by immunostaining with pH3(Ser10), a master driver of mitosis [45,46]. We found that in acute hypoxia, the intensities of SUMO2 and AURKB were significantly less on the mitotic chromosomes and during cytokinesis (Figure 4A,B,F and Figure S5). In addition, the pH3(Ser10) intensity was less (Figure 4A,B,D). However, the low levels of AURKB and pH3(Ser10), which reached mitotic chromosomes, were mainly SUMOylated, as indicated by a high colocalization coefficient (R) in acute hypoxia (Figure 4G,H). In the non-cycling cells, hypoxic conditions showed higher cytoplasmic colocalization of SUMO2-pH3(Ser10) (Supplementary Figure S6).

### 3.3. pH3(Ser10), a Master Regulator of Mitosis, Is a Target of SUMO2 and Is Abnormally SUMOylated under Acute Hypoxic Conditions

**Figure 5:**
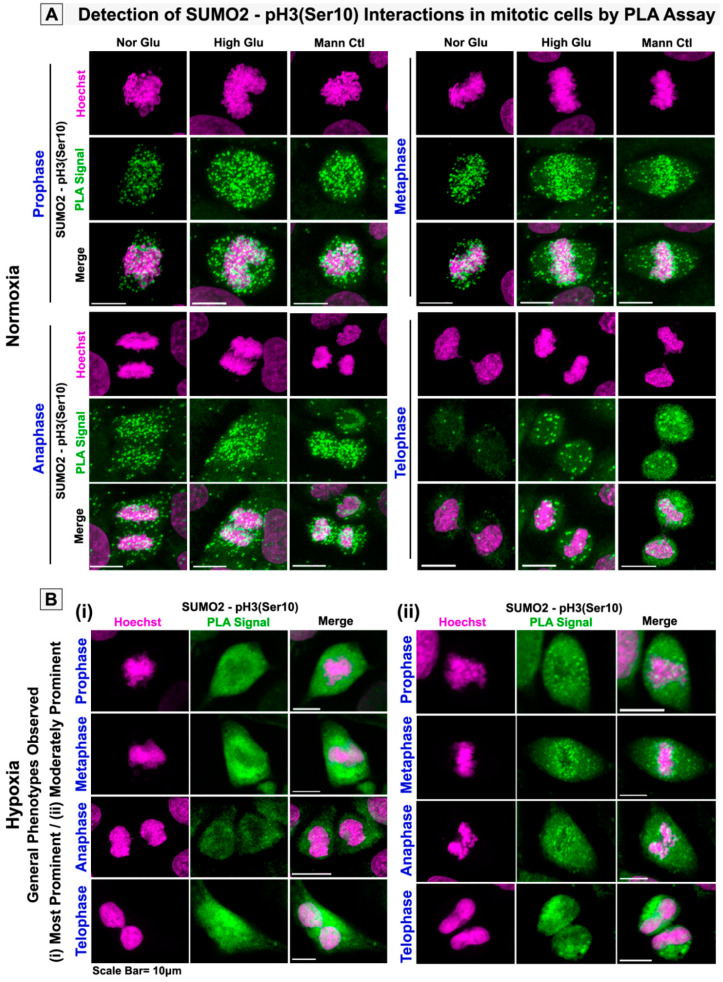
**Proximity ligation assay shows that acute hypoxia triggers cytoplasmic sequestration of SUMO2-modified pH3(Ser10) in mitotic cells.** CaSki cervical cancer cells were cultured in the general experimental set-up for 96 h. (**A**) Protein–protein interaction detection assay, the proximity ligation assay (PLA), showed enhanced localization of SUMO2-pH3(Ser10) PLA signal (green dots) in both HG and HG-HO conditions vs NG in normoxia. (**B**) On the contrary, the PLA signal was prominent in the cytoplasm and around the chromosome periphery in hypoxia-treated cells. Images shown in panel (**B**) (**i**) and (**ii**) represent observations across treatment conditions, irrespective of HG and HG-HO. See related Supplementary Figure S7 for correlated results in acute hypoxia-exposed non-mitotic tumour cells.

**Figure 6:**
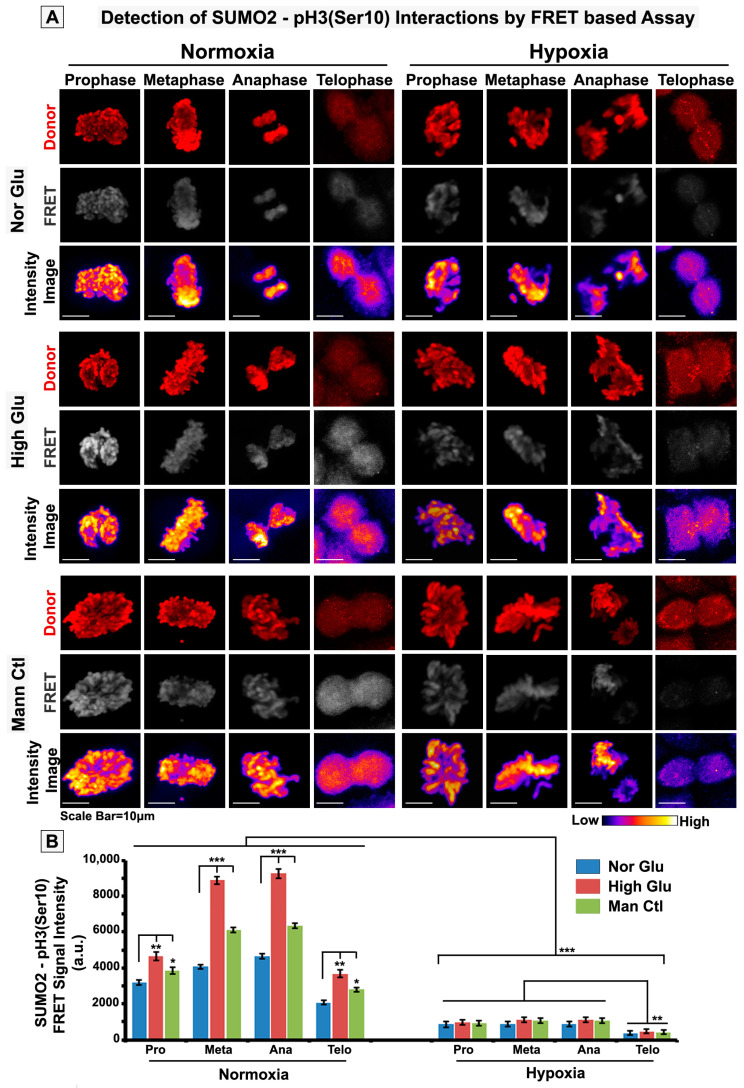
**Direct FRET between pH3(Ser10) and SUMO2 confirms cytoplasmic sequestration of SUMOylated pH3(Ser10) in acute hypoxia exposed mitotic cells.** CaSki cervical cancer cells were cultured in the general experimental set-up for 96 h. (**A**,**B**) Cells were immunostained with pH3(Ser10) and SUMO2 primary antibodies and were further tagged with Cy3 and Alexa flour 647 conjugated secondary antibodies, respectively, as Cy3-Alexafluor 647 are established FRET compatible donor–acceptor pairs. Analysis showed enhanced chromosomal localization of SUMO2-pH3(Ser10) FRET signal in both HG and HG-HO conditions vs NG in normoxia. On the contrary, the FRET signal was prominent in the cytoplasm of hypoxia-treated cells. Statistical significance is represented by *p* values: * *p* ≤ 0.05, ** *p* ≤ 0.01 and *** *p* ≤ 0.001. See related Supplementary Figure S8 for correlated results in acute hypoxia-exposed non-mitotic tumour cells.

The extensive colocalization of pH3(Ser10) with SUMO2 in the cytoplasm of tumour cells exposed to acute hypoxia prompted us to explore whether pH3(Ser10), a master mitotic driver, is a novel substrate of SUMO2. As SUMOylation is associated with nuclear trafficking and chromosomal loading of various cell-cycle-associated proteins, we wanted to examine whether appropriate SUMOylation of pH3(Ser10) is required for efficient nuclear localization and whether this process is predominantly hindered under acute hypoxia, which may result in observed retarded growth. In situ protein–protein interaction signal detection through proximity ligation (PLA assay) and FRET assay confirmed that a significant pool of pH3(Ser10) is conjugated to SUMO2 in both mitotic and non-mitotic cells (Figure 5, Figure 6 and Figures S7 and S8). The SUMOylated pH3(Ser10) levels were significantly higher in the cytoplasm and lowered on the mitotic chromosomes of cells exposed to acute hypoxia. In addition, cytoplasmic SUMOylated pH3(Ser10) showed aggregated organization in acute hypoxia-induced cells (Supplementary Figures S7 and S8, white arrows).

### 3.4. The Patient Tumour Tissue Array Validates the Extensive Cytoplasmic Sequestration of SUMO2-Modified pH3(Ser10) in Highly Hypoxic Regions

**Figure 7:**
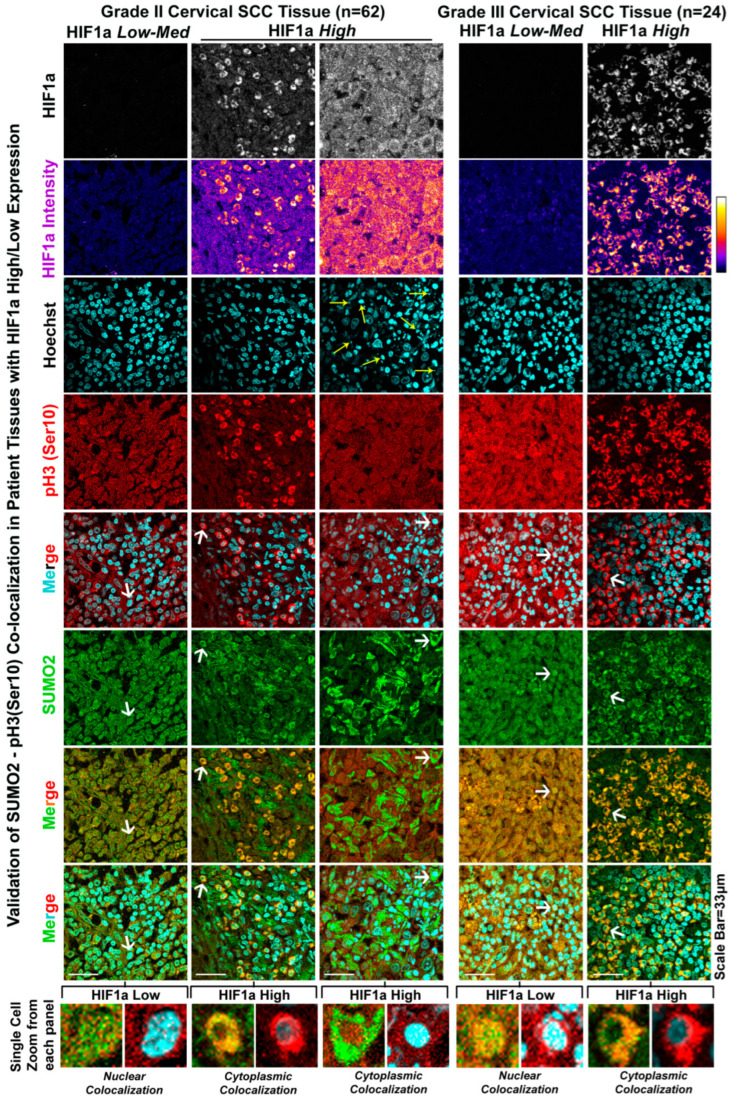
**High HIF1a-expressing regions in cancer tissues validate the role of hypoxia in cytoplasmic sequestration of SUMO2-pH3(Ser10).** Grade II and III cervical squamous cell carcinoma tissues showed hypoxic (HIF1a high) and normoxic (HIF1a low–medium) areas within the same block. Cytoplasmic localization of pH3(Ser10) and SUMO2 was observed in HIF1a-positive cells. On the contrary, HIF1a low to medium expressing regions showed appreciable nuclear colocalization. White arrowheads indicate the single cell with zoomed images in the lowermost panel. Yellow arrows indicate the condensed chromatin associated with apoptosis in acute hypoxic regions. See related Supplementary Figures S10–S15 for extended validation on uterine cervical cancer grade I, grade III and brain, breast colon, pancreatic, skeletal muscle, liver, and stomach cancer tissues.

In order to validate the correlation between cytoplasmic sequestration of SUMOylated pH3(Ser10) in hypoxia ‘very high’ regions in the context of actual clinical samples, we performed robust immunohistochemistry-based examination of various cancer tissues [47,48]. We confirmed that very high HIF1a-expressing cells have significant cytoplasmic localization of SUMO2-pH3(Ser 10) vs the nuclear enrichment in HIF1a ‘low or medium regions’ (Figure 7 and Figures S9–S15). Since highly hypoxic regions develop in advanced stages of tumour progression, it is noteworthy that significant cytoplasmic colocalization of SUMO2-pH3(Ser10) was identified in HIF1a-positive regions of advanced vs. lower tumour grades (Figure 7 and Figures S11–S13).

### 3.5. Nuclear Trafficking of SUMO2-Conjugated pH3(Ser10) Is Significantly Less in Acute Hypoxia Due to Its Phase Separation (LLPS) and Aggregation in the Cytoplasm

We were curious to know what causes cytoplasmic sequestration of SUMOylated pH3(Ser10) in acute hypoxia. SUMO2 can bind to its substrate as a polySUMO chain (chain of SUMOs bound to each other) or can conjugate as individual residues at one or multiple sites. We first tried to seek insights into how SUMO2 binds to pH3(Ser10). For this, we ran docking and molecular simulations experiments and found that SUMO2 can bind efficiently to pH3(Ser 10) in the tail region (Supplementary Figure S16A). This binding was found to be very strong for the SUMO2 isoform vs other isoforms. 

The tail region of histones is intrinsically disordered and is robustly post-translationally modified to enable various cellular, molecular and biological functions of histones. Co-relatively, two globally constant properties of SUMO2, documented for nuclear localization of its substrates, are the substrate binding preference in the disordered regions of proteins and SUMO-phosphorylation co-modification [44,49]. We identified the intrinsically disordered amino acid sequences in the tail region of pH3(Ser10) to which SUMO2 binds. The crucial lysine in the SUMO binding motif was identified to be at the 9th position in the sequence, near the phosphorylated residue, which is at the 10th position (Supplementary Figure S16B). In addition, the binding was specific to histone H3, which was phosphorylated at the Ser10 amino acid residue vs Ser 28, which is another phosphorylation site within this protein (Supplementary Figure S16C).

**Figure 8:**
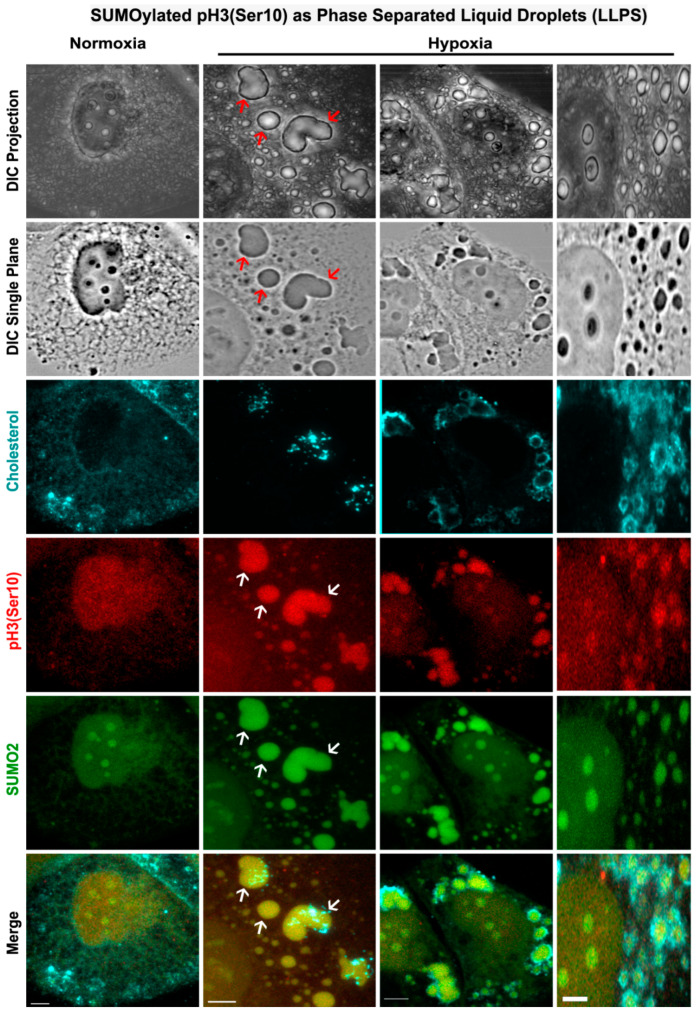
**SUMO2-conjugated pH3(Ser10) shows liquid–liquid phase separation (LLPS) in the cytoplasm of tumour cells exposed to acute hypoxia.** Cells were subjected to treatment conditions for 96 h. Zoomed images obtained through high-resolution confocal microscopy showed liquid–liquid phase partitioning of SUMO2-pH3(Ser10) in the cytoplasm of hypoxia exposed cells. An example of LLPS is indicated by red arrows in the DIC images and white arrows in the colour images. The LLPS-like regions showed punctate staining of cholesterol around the periphery, indicating that these membraneless aggregates were surrounded by membranous organelles.

Intrinsically disordered proteins are highly susceptible to protein aggregation, condensation and liquid–liquid phase separation (LLPS). Since histone H3 variants have significantly high coverage of disordered sequences (Supplementary Figure S16B), we reasoned that any aberrant post-translational modification (PTM) of the histone H3 variant could condense the protein into LLPS, which may significantly impact its nuclear trafficking, cell-cycle-associated functions and its role in cell proliferation [49]. We further speculated that acute hypoxia-induced abnormal site SUMOylation could be the type of PTM that sequesters the pH3(Ser10) in the cytoplasm via LLPS formation [50]. It could thereby explain the M-phase arrested phenotype in hypoxia. To this argument, we examined the tumour cells under NG, HG and HG-HO conditions in normoxia and hypoxia through a high-resolution confocal microscope. We found convincing evidence of SUMO2-pH3(Ser10) membraneless cytoplasmic phase separation (Figure 8). Intriguingly, membranous organelles, identified by membrane labelling with an anti-cholesterol antibody, frequently bordered these LLPS regions, like fences. The significance of this observation is yet to be clear. However, it could be that these organelles are randomly trapped at the periphery of the phase-separated aggregates, or it could be that these organelles actively border the LLPS to prevent its further spread in the cell, as LLPS expansion can invariably choke the cells to death. The latter possibility is likely to help the tumour cells survive acute hypoxia while lying dormant [51,52].

### 3.6. Abnormal Activity of SENPs, under Acute Hypoxia, Sequesters SUMOylated pH3(Ser10) in the Cytoplasm via LLPS, Thereby Generating Tumour Cell Growth Arrested Phenotype

**Figure 9:**
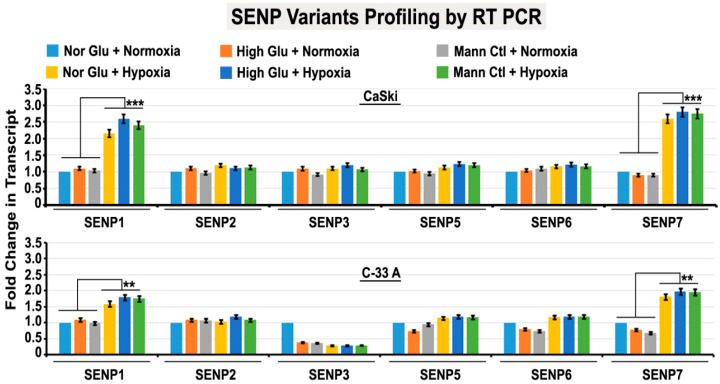
**Acute hypoxia upregulates SUMOylation regulatory proteins SENP1 and SENP7.** Tumour cells were subjected to the basic treatment conditions for 96 h. Real-Time-PCR-based data showed a significant upregulation in the transcript of SENP1 and SENP7 in acute hypoxia-induced conditions vs normoxia. All comparisons were made with NG normoxic condition unless indicated in the graph. Statistical significance is represented by *p* values: ** *p* ≤ 0.01 and *** *p* ≤ 0.001.

**Figure 10:**
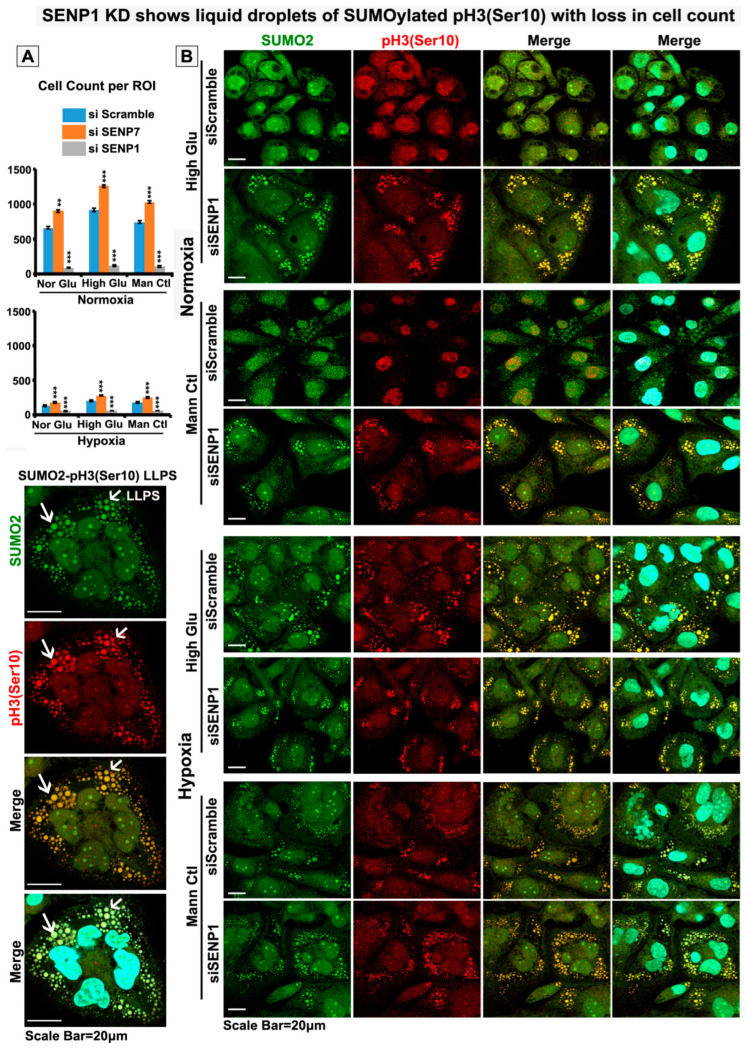
**siRNA-mediated knockdown of SENP1 and SENP7 reveals their opposite roles in SUMOylated pH3(Ser10) LLPS formation and tumour cell loss.** SENP1 and SENP7 were downregulated independently by the siRNA technique. Post downregulation, tumour cells were subjected to basic treatment conditions for 96 h. (**A**) Cell count was measured in Fiji image analysis software. (**B**) SENP1-depleted tumour cells were cultured under normoxic and hypoxic NG, HG and HG-HO conditions. Cytoplasmic sequestration of SUMO2-pH3(Ser10) in LLPS was determined. White arrows in zoomed image points to LLPS formation. The same experiment was performed in SENP7-depleted tumour cells, as shown in Supplementary Figures S17 and S18. All comparisons were made with NG normoxia conditions unless indicated in the graph. Statistical significance is represented by *p* values: ** *p* ≤ 0.01 and *** *p* ≤ 0.001.

Since SUMO2 conjugation and de-conjugation (de-SUMOylation) are regulated by sentrin proteases, we profiled the transcripts of SENP isoforms. We found that SENP1 and SENP7 were significantly high in acute hypoxia (Figure 9). Subsequent RNA interference studies showed that SENP7 downregulation enhanced proliferation in both hypoxia and normoxia, supported by an observed increase in cell counts in SENP7 downregulation, and the reverse was observed when SENP1 was downregulated (Figure 10A and Figure S17). This suggests that SENP7 does not support survival, while SENP1 does. Interestingly, SENP1 downregulation generated SUMO2-pH3(Ser10) LLPS even in normoxia, and in hypoxia, the LLPS-like phenotype intensified (Figure 10B and Figure S18). SENP1 downregulation can prevent SUMO2 substrate degradation and promote its abnormal accumulation. These observations again suggest that SENP1 is pro-survival and is not involved in LLPS generation in acute hypoxia [53,54,55,56,57,58,59]. It further indicates that SENP7 may be involved in LLPS generation, which is restricted by SENP1, due to which a few dormant stem-like tumour cells resist acute hypoxia challenge. SENP7’s polySUMO chain length shortening activity is structurally specific for SUMO2-conjugated substrates [56]. Therefore, in hypoxia with elevated levels of SENP7, the SUMO2 nuclear signal was less vs other isoforms (although some punctate cytoplasmic signals were observed for other isoforms as well in hypoxic vs normoxic conditions) (Supplementary Figure S19).

**Figure 11:**
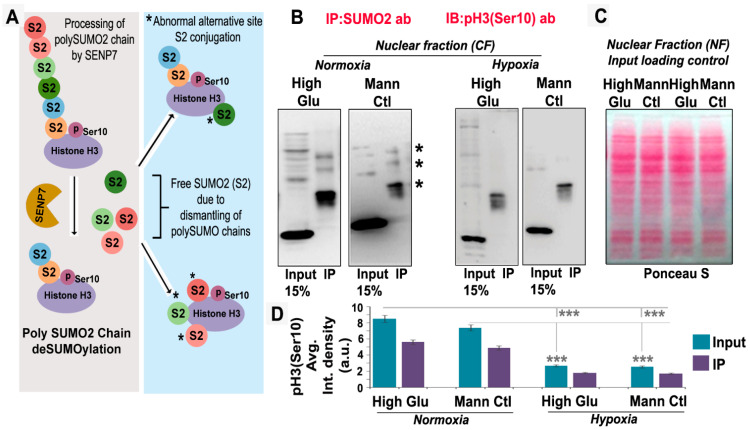
**Acute hypoxia promotes abnormal SUMOylation of pH3(Ser10), likely through SENP7.** (**A**) SENP7 is specific to the SUMO2 substrate, and its polySUMO2 chain shortening and deSUMOylation activity can generate free SUMO2 residues that can then bind to alternative abnormal sites in the same protein. Either way, SUMO2 chain shortening or abnormal multisite SUMOylation can compromise pH3(Ser10) solubility, nuclear trafficking and functions. (**B**) Tumour cells were subjected to the basic treatment conditions for 96 h. Nuclear lysates were prepared through subcellular fractionation and immunoprecipitated (IP) with SUMO2 antibody. The IP samples were immunoblotted for pH3(Ser10). Data unveiled that there was a loss of higher molecular weight pH3(Ser10) bands in hypoxia (see asterisks), and the levels of pH3(Ser10) by itself were less in input and IP samples in comparison to normoxic conditions. (**C**) Ponceau S staining of the IP samples showed equal loading. Further, input from the hypoxia sample showed lower molecular weight bands not identified in the normoxia sample. This suggests that SUMOylated pH3(Ser10) that managed to enter the nuclei of hypoxic tumour cells was differentially modified by SUMO2 vs normoxia counterparts. (**D**) Nuclear levels of total pH3Ser(10) in the input and SUMOylated pH3(Ser10) in the IPed samples were quantitated in HG and HG-HO conditions under normoxia and hypoxia. Data were represented as Average Integrated Density. Data show that in hypoxic HG and HG-HO conditions, total and SUMOylated pH3(Ser10) were significantly less compared to normoxic conditions. Statistical significance is represented by *p* values: * *p* ≤ 0.05, ** *p* ≤ 0.01 and *** *p* ≤ 0.001.

Further, the SUMO2 substrate, such as AURKB, was not associated with a significant LLPS phenotype in hypoxia, although its nuclear trafficking was inhibited (Supplementary Figure S20). Therefore, LLPS formation is very substrate-specific to certain SUMO targets only [57]. SENP7 is predominantly involved in the deSUMOylation of polySUMO chains, causing shortening in the polySUMO2 chain conjugated to its substrate. The SUMO2 freed from the polySUMO chain can further bind to the same substrate at other conducive sites via the activity of ligases (Figure 11A, diagrammatic representation of SENP7 action). Thus, the nature of SUMOylation and positional location of SUMOylation cumulatively determine the substrate’s physical state, solubility and functionality in a context-dependent manner. Therefore, polySUMO chain deSUMOylating SENPs can lead to the loss of SUMO substrate functions or vice versa. To test abnormal SUMO processing of pH3(Ser10), we needed to perform the immunoprecipitation experiments either from the adherent cell lysates or the cytoplasmic fraction, which were technically challenging for the hypoxic conditions due to the formation of LLPS. However, we extracted the nuclear lysates from tumour cells in HG and HG-HO hypoxic and normoxic conditions, and via co-immunoprecipitation with the SUMO2 antibody, profiled the SUMOylation states of pH3(Ser10) that managed to enter the nucleus (Figure 11B). The input showed relatively less levels of the pH3(Ser10) pool in the hypoxic nuclei. The higher molecular weight bands of SUMOylated pH3(Ser10) were missing in the immunoprecipitated hypoxic samples (Figure 11B, indicated by asterisks). In addition, the SUMOylated pH3(Ser10) levels were less (Figure 11C). This suggests that a pool of SUMOylated pH3(Ser10) that managed to enter the hypoxic nuclei was majorly differentially modified by SUMO2 and, thereby, may not be efficient in association with chromosomes, as documented in Figure 4, Figure 5 and Figure 6. This is correlated with the observation that depletion of SENP7 allows more SUMOylated pH3(Ser10) to enter the nuclei and thereby promotes proliferation (Supplementary Figure S17).

### 3.7. Momordin Ic (MC), an SENP1 Inhibitor, Can Mimic Hypoxia-Induced Abnormal SUMOylation of pH3(Ser10) and Thereby Retards Tumour Cell Proliferation in Normoxic High-Glucose Hyper-Osmotic Conditions

**Figure 12:**
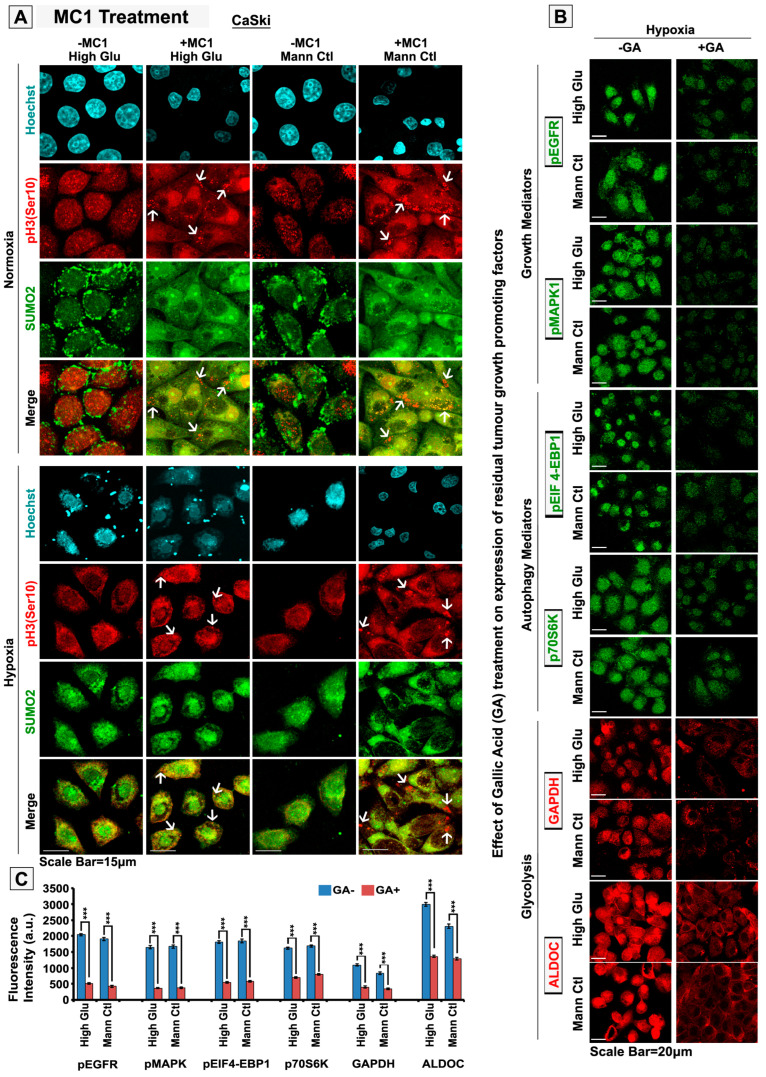
**Momordin Ic (MC), an SENP1 inhibitor, can hijack mechanoadaptation of normoxic high-glucose hyper-osmotic tumour cells by promoting abnormal SUMOylation of pH3(Ser10).** Cells were subjected to the basic treatment conditions for 96 h, followed by treatment with 25 µM Momordin Ic for 6, 12, 24 and 48 h.(**A**) Confocal images show massive cytoplasmic colocalization of SUMO2-pH3(Ser 10) in normoxic experimental conditions vs untreated conditions (see white arrows). In hypoxia, the treatment enhanced cytoplasmic sequestration of SUMO2-pH3(Ser10). (**B**,**C**) In order to more robustly eliminate the residual growth in hypoxia, the tumour cells were treated with 100 µM of Gallic Acid (GA), post 96 h of treatment in the standard experimental conditions. Gallic-Acid-treated cells were immuno-probed for key survival players such as pEGFR(1173), pMAPK1, pEIF-4EBP1, P70S6K, GAPDH and ALDOC. Confocal image analysis showed reduced levels of probed molecules in GA treated vs untreated conditions. Statistical significance is represented by *p* values: *** *p* ≤ 0.001. See related Supplementary Figures S22 and S23.

Our observations so far suggested that SENP1 downregulation drove SUMOylated pH3(Ser10) into an LLPS-like phenotype in normoxic conditions, thereby inhibiting the cell proliferation functions of pH3(Ser10) and retarding tumour growth (Figure 10A,B). It suggests that we need to reduce the levels of SENP1 to promote the abnormal accumulation of SUMO2-pH3(Ser10), so that it can stall mitosis. Normal cells are also expected to recruit SUMO2 substrates for mitotic events, but the required levels are lower than cancer cells [58,59]. In addition, SENP1 expression is also lower in normal cells, so SENP1 inhibition and resultant aberrant SUMO2 substrate accumulation would be far less in normal cells. We used Momordin Ic (MC), a natural SENP1 inhibitor, to produce the growth-retarding effects associated with abnormal SUMO2 processing of pH3(Ser10) [37]. MC concentrations were titrated for non-toxic effects on normal cells and were determined to be 25 µM (Supplementary Figure S21). Upon Momordin Ic treatment, we noticed an increase in SUMO2-pH3(Ser10) abnormal colocalization. In addition, in several regions of the tumour cell, pH3(Ser10) was observed to form large aggregates (Figure 12A, white arrows). Within 12 h of MC treatment, a significant rise in mitotic cell numbers was noticed in normoxic HO and HG-HO conditions (Supplementary Figure S22). However, by 24 h of MC treatment, there was a significant loss in cell count. It suggests that cells first underwent M-phase arrest, which manifested as higher mitotic cell numbers per field of view, but there was no actual growth. By the next 24 h of treatment (total of 48 h), most cells lifted off the substrate and died by anoikis, as determined by trypan blue hemocytometry-based live/dead assay.

The effect of MC on residual cell growth associated with acute hypoxia was far less, probably because SUMOylated pH3(Ser10) was already inhibited from associating with the mitotic chromosomes (Supplementary Figure S23). In addition, there was a pre-existing M-phase arrest. 

**Figure 13:**
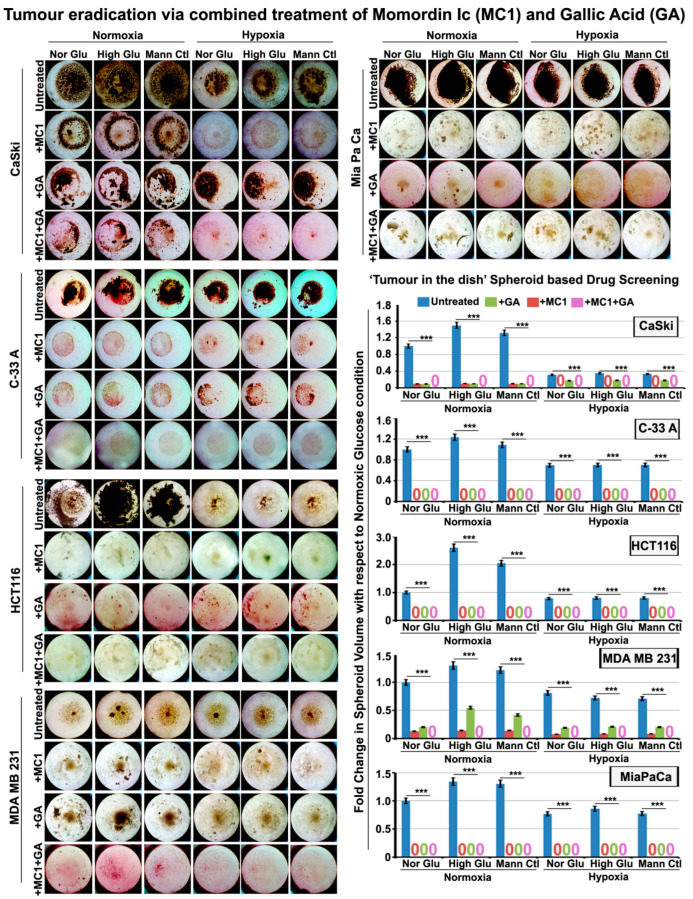
**Momordin Ic (MC), an SENP1 inhibitor, and Gallic Acid combination treatment can eliminate tumour cell survival in normoxia and hypoxia.** Three-dimensional tumour spheres generated from human cervical, colon, breast and pancreatic cell lines were subjected to the basic treatment conditions for 96 h, followed by treatment with 25 µM Momordin Ic (MC) or 100 µM Gallic Acid (GA) or in combination for the next 9 days. Data revealed that the individual treatments alone could reduce the tumour spheroid growth. However, the combined treatment of Momordin Ic and Gallic Acid had a significant synergistic effect in attenuating the tumour cell growth in both normoxia and hypoxia across all the cell lines shown. The numerical “zero” in graph indicates no spheroid growth in the mentioned conditions. The representative images are shown for the growth status of the spheres on the 9th day. Statistical significance is represented by *p* values: *** *p* ≤ 0.001.

**Figure 14:**
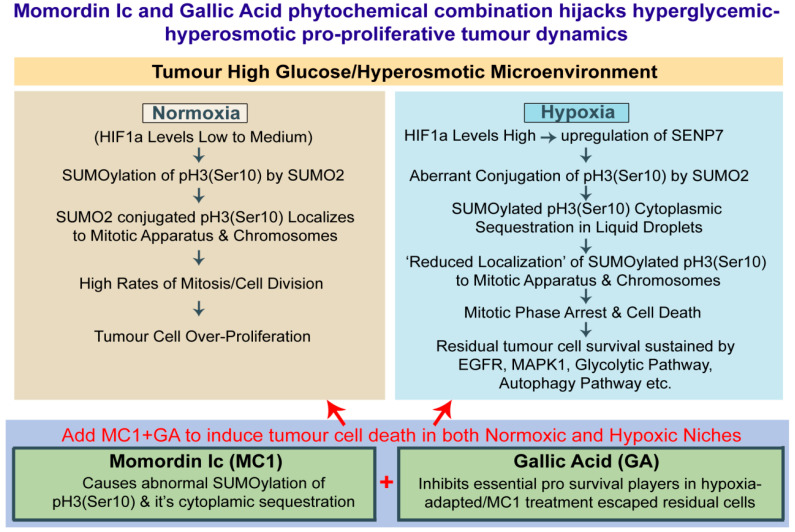
**Momordin Ic (MC) and Gallic Acid phytochemical combination can hijack tumour cells’ pro-proliferative mechanoadaptation to high-glucose hyper-osmotic stress.** In summary, it was found that in the normoxic HG microenvironment, tumour cells proliferate extensively as a biophysical adaptation to high-glucose hyper-osmotic stress (HG-HO), which predominantly involves an appropriate SUMOylation of pH3(Ser10). Acute hypoxia triggers the accumulation of abnormally SUMOylated pH3(Ser10) due to the upregulated activity of SENP7 that leads to mitotic arrest. Inhibition of SENP1 via Momordin Ic (MC) can cause similar M-phase arrest in proliferating normoxic cells, irrespective of HG and HG-HO pro-proliferative stress. The M-phase arrested population could be cleared by suppressing residual growth signalling via Gallic Acid (GA) treatment. Both MC and GA are enriched in *Momordica charantia*.

In order to kill the hypoxia-induced residual cell survival, which possesses a capacity for re-growth, a robust strategy was needed. In our previous experience, we reported that the plants from which the bioactive drug molecule is derived also have many other chemotherapeutics and chemosensitizers [34]. Generally, the administration of plant extracts from which the active molecule is derived is preferred in traditional medicine because such plants are enriched in triterpenes and flavonoids in the correct concentrations to harm tumorous growth selectively. We therefore scrutinized the chemical composition of *Momordica charantia* (enriched in Momordin Ic) and pinned our hope on the Gallic Acid found in it, as the literature survey showed that it could target major cancer survival players such as EGFR, MAPK, autophagy effectors, glycolysis, etc. [38,60,61,62,63,64,65]. We titrated the Gallic Acid effective dose that is non-toxic to normal cells (Supplementary Figure S24). We tested its effects on lowering the growth factors such as EGFR and MAPK1, autophagy effectors such as pEIF4EBP1 and p70-S6K (RPS6KB1) and glycolysis regulators ALDOC and GAPDH (Figure 12B). Next, we tested MC and GA’s individual and combined effects in the complex tumouroid assays with both bulk and cancer stem cell populations. This combination therapy was efficient on cervical cancer cells and other tumour cells (Figure 13). The floating cells emerging from this treatment were dead, and even when these pooled floating cells were replated in conducive conditions, no growth was observed for two months. The outcome of this study is summarized in Figure 14.

## 4. Discussion

High glucose (HG) is a significant hallmark of tumour progression. However, targeting known glucose signalling, glucose transporters and metabolic enzymes in cancer has been a concern, as it adversely impacts normal physiology. Since high glucose is also an osmotic stressor to which cancer cells intriguingly adapt, we were enthusiastic about teasing out the most prominent “hyper osmolarity adaptative mechanism” with the hope that targeting it may selectively promote the death of tumour cells without affecting the normal cells [8,32,33]. Due to interstitial fluid flow, which bathes the entire tumour tissue, high glucose can even be carried to the hypoxic interiors and exert its hyper-osmotic challenge. Therefore, by molecularly dissecting how tumour cells reshape their gene signatures and adjust to the high-glucose hyper-osmotic stress and oxygen-tension-associated dual biophysical challenge, we were motivated that we may be able to attenuate the mechanoadaptation therapeutically and consequently induce selective vulnerability to death.

In a cervical cancer cell model, we report that high glucose and its osmotic mimetics, the mannitol and l-glucose at similar concentrations, promote comparable proliferation effects in normoxia. However, this effect is attenuated in an acutely hypoxic microenvironment, and cells enter dormancy due to mitotic arrest. The mechanism associated with this effect was related to hypoxia-induced excessive accumulation of SUMO2-modified pH3(Ser10) in the cytoplasm, compromising its availability for mitosis progression. Excessive phosphorylation of histone H3 and its variant H3.3 at the Ser 10 position has been associated with aggressive tumours, and it is a known master driver of mitosis [66,67]. Stalling of dynamic mitotic rounding, due to less chromosomal association of SUMOylated pH3(Ser10) in hypoxia, could inhibit tumour cells from balancing their cortical tension to osmo-adaptive values and thereby cause the death of mitotic cells that are under arrest for long time [68,69,70].

SUMO conjugation and substrate stabilization are regulated by the sentrin-specific proteases family of proteins, which has both redundant and unique functions [50]. Acute hypoxia was found to trigger SENP1 and SENP7 isoforms, but SENP1 was identified to be pro-survival. SENP1 higher levels have been associated with tumorigenesis [53,54,55,70]. We found that the over-activity of SENP7 promotes differential processing of SUMOylated pH3(Ser10), which shortens the SUMO2 chain bound to the substrate, impacting pH3(Ser10) solubility. Further, the liberated SUMO2 residues, in effect, likely bind to the alternative sites, rendering pH3(Ser10) accumulation in the cytoplasm with the LLPS-like M-phase phenotype.

Induction of mitotic arrest by various anti-cancer drugs has been attractive for drug development because it controls metastasis and circulating tumour cells [68,69,71]. However, we find that such mitotically arrested, adherent and anchorage-independent phenotypes can relapse upon arrival of conducive conditions. Therefore, not only mitotic arrest but attenuation of residual survival cell signalling needs to be targeted simultaneously. However, the first step is to render the tumour dormant for the ‘kill while they sleep’ approach. Based on our observation, we could choose between two routes: the first was to upregulate SENP7 activity in normoxia to induce the LLPS phenotype and promote G2/M arrest. Alternatively, we could deplete SENP1, as our observation suggests that this could lead to massive abnormal accumulation of SUMOylated pH3(Ser10) due to lack of proteasomal degradation. Due to the availability of the natural SENP1 inhibitor Momordin Ic, we chose the second option. Momordin Ic treatment led to excessive accumulation of SUMOylated pH3(Ser10) in the cytoplasm akin to a hypoxic condition and induced dormancy in the normoxic HG-HO conditions. Clinical samples from cervical and other cancer also showed high cytoplasmic accumulation of SUMOylated pH3(Ser10) in the acutely hypoxic zones (identified by high HIF1a expression). Thus, the study suggests that Momordin Ic could effectively hijack hyperglycaemic–hyper-osmotic mechanoadaptation, thereby inducing therapeutic vulnerabilities to cell death.

In order to further target the minimal residual growth generated in this treatment, we took the rational approach of delving deeper into the other phytochemicals that are enriched in plants from which MC is extracted and scored Gallic Acid for multiple reasons. Gallic Acid is known for its anti-cancer effects [62,63,64,65]. It can attenuate almost all hallmarks of cancer, such as inflammation, redox imbalances, hyperglycaemia, hyperlipidaemia, extracellular matrix stiffness, immune evasion, etc. Momordin Ic and Gallic Acid are already independently tested in animal models of tumours for safety and efficacy [37,38]. Therefore, plant extracts enriched in Momordin Ic and Gallic Acid, such as *Momordica charantia*, will be tested in further studies [72,73,74]. *Momordica charantia* is in Phase II clinical trials in modern medical practice (https://clinicaltrials.gov/ct2/show/results/NCT02397447, accessed on 21 February 2023); https://go.drugbank.com/drugs/DB14265, accessed on 21 February 2023). It is already a certified nutraceutical-based Ayurvedic medicine, a form of a medical practice prevalent in India and Asia. In addition, Gallic Acid is also enriched in other ayurvedic medicines such as Amalakirasayana and Arjunarishta [75,76,77].

## 5. Conclusions

In summary, we find that hyper-osmotic stress associated with high glucose concentrations drives tumour cell proliferation under normoxia, but the effect is attenuated under hypoxia due to G2/M-phase arrest. Mitotic-phase arrested tumour cells, in acute hypoxia, show extensive cytoplasmic aggregates of SUMO2 modified pH3(Ser10) due to liquid–liquid phase separation (LLPS). We validated this phenotype in high HIF1a regions of cancer tissues of different organs of origin. The high levels of SENP7 in acute hypoxia likely dismantle the polySUMO2 chain from pH3(Ser10), which promotes free and accumulated SUMO2 individual residues to bind to pH3(Ser10) at abnormal sites. This aberrant processing impacts pH3(Ser10) cytoplasmic solubility, generates the LLPS phenotype and thereby restricts its critical functions as a mitotic driver, causing tumour dormancy. Momordin Ic (MC), an SENP1 inhibitor, enhances SUMO2-pH3(Ser10) cytoplasmic accumulation in normoxic normal, high-glucose and hyper-osmotic conditions, thereby causing M-phase arrest and death of tumour cells. The combination of Momordin Ic with Gallic Acid (GA), both enriched in the extract of *Momordica charantia* (under Phase II clinical trials, available for sale under traditional medicine systems), significantly eliminates both normoxic active and hypoxic dormant tumour cells; therefore, this represents a promising lead in anti-cancer drug development. As high-grade patient tissues show the cytoplasmic accumulation of SUMO2-pH3(Ser10) in the HIF1a high areas, we propose SUMO2, pH3(Ser10) and HIF1a co-immunodetection as multiplexed panel biomarkers for prognosis and for predicting the probability of developing metastasis and relapse.

## Data Availability

Data is contained within the article or supplementary material.

## Supplementary Materials

The following supporting information can be downloaded at: https://www.mdpi.com/article/10.3390/cells12060825/s1, Supplementary Information Files: Supplementary Information Files comprising Supplementary Table S1 (Additional File S1) and Supplementary Figures and Methods (Additional File S2) accompany this manuscript and are available online. Supplementary Table S1: List of SUMO2 conjugated mitosis regulatory proteins obtained from Eifler et al., 2015 and MsigDB data; Supplementary Figure S1: General experimental set-up for all experiments; Supplementary Figure S2: HIF1a, a known hypoxia marker, showed stable expression by 48 h of treatment with 150 µM Cobalt chloride in the tumour cell culture medium; Supplementary Figure S3: Representative images of confluency, colony formation and soft agar assay in C-33A cervical cancer cells. Supplementary Figure S4: Cell fate analysis of tumour cells in normal, high glucose and hyperosmotic equivalent conditions under normoxia and hypoxia; Supplementary Figure S5: AURKB localization to the midbody was less in acute hypoxia-exposed tumour cells; Supplementary Figure S6: pH3(Ser10) is prominently colocalized with SUMO2 in the cytoplasm of acute hypoxia-treated cancer cells in contrast to a nuclear colocalization in normoxia; Supplementary Figure S7: Protein-Protein interaction detection assay, the proximity ligation assay (PLA), showed a cytoplasmic signal of pH3(Ser10) and SUMO2 in acute hypoxia treated cancer cells, irrespective of high glucose/hyperosmotic conditions; Supplementary Figure S8: Direct FRET between pH3(Ser10) and SUMO2 confirms cytoplasmic sequestration of SUMOylated pH3(Ser10) in acute hypoxia; Supplementary Figure S9: The details of the US Biomax uterine cervical cancer tissue array used to generate data are shown in main Figure 7 and Supplementary Figures S10–S15; Supplementary Figure S10: Cytoplasmic colocalization of pH3(Ser10) and SUMO2 is observed in HIF1a high regions of grade I cervical squamous cell carcinoma tissues; Supplementary Figure S11: Significant cytoplasmic colocalization of pH3(Ser10) and SUMO2 is observed in HIF1a high regions of grade III cervical squamous cell carcinoma tissues, which are negative for the HPV16; Supplementary Figure S12: Significant cytoplasmic colocalization of pH3(Ser10) and SUMO2 is observed in HIF1a high regions of grade III cervical squamous cell carcinoma tissues, which are also positive for HPV16; Supplementary Figure S13: Cytoplasmic colocalization of pH3(Ser10) and SUMO2 is observed in HIF1a high regions of Grade III uterine adenocarcinoma patient tissues; Supplementary Figure S14: Cytoplasmic colocalization of pH3(Ser10) and SUMO2 is observed in HIF1a high regions of brain, breast and colon cancer patient tissues; Supplementary Figure S15: Cytoplasmic colocalization of pH3(Ser10) and SUMO2 is observed in HIF1a high regions of pancreatic, skeletal muscle, liver, skin and stomach cancer patient tissues; Supplementary Figure S16: Molecular dynamics simulations predict a stable conjugation of SUMO2 to pH3(Ser10) in the disordered tail region of the protein; Supplementary Figure S17: siRNA-mediated knockdown of SENP7 resulted in increased nuclear localization of pH3(Ser10) in tumour cells exposed to hypoxic conditions; Supplementary Figure S18: siRNA-mediated knockdown of SENP1 resulted in decreased nuclear localization of pH3(Ser10) in normoxic normal glucose conditions, whereas the reverse was observed with SENP7 knockdown in tumour cells; Supplementary Figure S19: SUMO isoforms 1, 3, and 4 can localize to tumour cells’ nuclei in both hypoxic and normoxic conditions; Supplementary Figure S20: The prominent nuclear localization of AURKB observed in normoxia was abrogated in the acute hypoxic conditions, but no LLPS phenotype was observed; Supplementary Figure S21: Momordin Ic dose titration by MTT assay showed that the normal cells were unaffected up to a concentration of 25 µM; Supplementary Figure S22: Under both hypoxia and normoxia, high glucose conditions showed mitotic cell cycle arrest, in tumour cells, upon treatment with Momordin Ic; Supplementary Figure S23: Under both hypoxia and normoxia, high glucose hyperosmotic stress conditions showed mitotic cell cycle arrest in tumour cells upon treatment with Momordin Ic; Supplementary Figure S24: MTT assay showed the viability of normal cells was not affected by the individual or combined doses of Momordin Ic and Gallic Acid drug treatments in this investigation

## Abbreviations

4e-BP1: eukaryotic translation initiation factor 4E-binding protein 1;a.u.: arbitrary units; ALDOA: Aldolase, Fructose-Bisphosphate A; ALDOB: Aldolase, Fructose-Bisphosphate B; ALDOC: Aldolase, Fructose-Bisphosphate C; AURKB: aurora kinase B; BSA: Bovine Serum Albumin; CoCl 2: Cobalt Chloride; ECM: extracellular matrix; EGFR: Epidermal Growth Factor Receptor; FACS: fluorescence-activated cell sorting; FRET: Förster Resonance Energy Transfer; GA: Gallic Acid; GAPDH: glyceraldehyde 3-phosphate dehydrogenase; HG: high glucose; HG-HO: high-glucose-mediated hyper-osmotic stress; HIF1a: hypoxia-inducible factor alpha; LLPS: liquid–liquid phase separation; MC: Momordin Ic; P70S6K: Phospho70 Ribosomal protein S6 kinase; PFA: Paraformaldehyde; PLA: proximity ligation assay; pH3(Ser 10): histone H3 phosphorylated at Ser10; ROI: regions of interest; RT: room temperature; RT-PCR: Reverse Transcription Polymerase Chain Reaction; SD: Standard Deviation; SENP: SUMO-specific proteases; siRNA: Small Interfering RNA; Sox2: SRY-Box Transcription Factor 2;SUMO: Small Ubiquitin-related Modifier; TMA: tissue microarray.
