# Supplementary material for: Understanding the Combined Effects of High Glucose Induced Hyper-Osmotic Stress and Oxygen Tension in the Progression of Tumourigenesis: From Mechanism to Anti-Cancer Therapeutics
Source: Cells. 2023 Mar 7;12(6):825. doi: 10.3390/cells12060825 (PMC10047272; doi:10.3390/cells12060825)
Supplement: Supplementary file 1 [file cells-12-00825-s001.zip › Additional File 2 - Supplementary Methods and Figures.pdf]

Additional File 2

Supplementary Materials For

Understanding the Combined Effects of High Glucose Induced Hyper-Osmotic Stress and Oxygen Tension in the Progression of Tumourigenesis: From Mechanism to Anti-Cancer Therapeutics

Gayathri K. G.[1,2], Puja Laxmanrao Shinde[1,2,†,] Sebastian John[1,2,†,] Sivakumar K. C. [3] and Rashmi Mishra[1,2,*]

[1]Laboratory of Translational Mechanobiology, Rajiv Gandhi Centre for Biotechnology, Thiruvananthapuram 695014, Kerala, India

[2]Manipal Academy of Higher Education, Manipal 576104, Karnataka, India

[3]Distributed Information Sub-Centre (Bioinformatics Centre), Bio-Innovation Centre (BIC), Rajiv Gandhi Centre for Biotechnology, Thiruvananthapuram 695014, Kerala, India

*Correspondence: rashmimishra@rgcb.res.in or rashmi.mpi.cbg@gmail.com

†These authors contributed equally to this work.

<u>This file includes:</u>

I) Supplementary Section S1: Supplementary Methods

II) Supplementary Section S2: Supplementary Figures Figs.S1-S24 with Figure Legends

Supplementary Section S1

SUPPLEMENTARY METHODS

1) Cell proliferation and Colony Formation Assay:

For cell proliferation assay, CaSki/C-33 A human cervical cancer cells were cultured for 96 hours in 5.5mM physiological levels of glucose, high glucose and hyper-osmotic stress equivalents (Mannitol and L-glucose), with or without hypoxia stimulation. Motorised stitching of the wells was done for image representations and analysis. The cell proliferation was determined by thresholding and counting using Fiji image analysis software.

For the colony formation assay, 3000 cells were seeded per well in a 6-well plate, and the treatments were started 24 hours post-seeding. The treatments were continued till individual colonies were formed from the single cells. Once individual colony formation units consisting of greater than or equal to 50 cells per colony were formed in the control well (which took 8 to 14 days, depending on the cell line), the colonies were fixed with Glacial acetic acid: Methanol in a 1:3 ratio solution for 5 minutes at RT, in the dark. The cells were stained with 0.5% Crystal violet solution in methanol in the dark for 15 minutes at RT. The stained 6-well plates were washed thoroughly using distilled water to remove the residual staining solution. The whole well images were acquired by motorised stitching using the 4x objective of the Olympus FV 3000 laser scanning confocal microscope with bright field settings. Colonies with more than 50 cells were scored using the Fiji software. The number of colony-forming units per well was graphically represented.

2) Soft Agar Assay:

Soft agar assay was performed to assess the effects of hyperglycemia, hyperosmotic stress and hypoxia on the anchorage-independent renewability of cervical cancer cells. A bottom layer of 0.5% agarose and a top layer of 0.3% agarose were cast with 3000 cells per well within the top layer of agarose gel. The treatments were started 24 hours post-seeding. After 21 days of incubation at 37°C, the spheroids were stained with 0.05% (w/v) crystal violet or processed for NBT/BCIP-based alkaline phosphatase assay, and the spheroids in each well were scored manually from the captured images. The individual spheroid images in 4x magnification were shown as a representative, and the number of spheroids in each condition was graphically represented.

3) BCIP/NBT Alkaline phosphatase Assay:

Alkaline phosphatase (AP) is one of the key markers in the identification of stem cells. Usually, this enzyme is enriched in membranes, but it is also enriched cytoplasmically in stem cells. 5-bromo-4-chloro-3-indolyl phosphate (BCIP)/ nitro blue tetrazolium (NBT) is an artificially manufactured chromogenic substrate of AP and enables colourimetric image-based detection of the abundance of AP. BCIP-NBT tablets (Sigma - B5655) were used for visualizing the alkaline phosphatase-positive cell population according to the manufacturer's recommendations. Briefly, the cells were fixed with pre-chilled methanol at -20°C for 5 minutes, and the staining solution, prepared by dissolving the tablet in water, was added to the wells. The plate was incubated overnight at RT, and the residual staining solution was washed off post-incubation, followed by drying the plate. Images were captured using the Olympus IX71 microscope.

4) Senescence Assay:

According to the manufacturer's recommendations, the senescence-associated β-Galactosidase expression was analyzed using the X-Gal reagent (Himedia - MB069). Briefly, the cells were fixed with 4% paraformaldehyde fixing reagent for 5 minutes at RT in the dark. After washing the cells with 1x PBS to remove any residual fixing reagent, X-Gal staining solution was added to the cells and was incubated for 12 hours at 37°C in the dark. Hydrogen peroxide was used as a positive control at 200 µM and 400 µM final concentration. Images were captured after the staining with the Olympus IX71 microscope.

5) Flow Cytometry:

2.5 x 10^5 cells were seeded in 100mm cell culture dishes. The treatments were started after 24 hours and were continued till 96 hours. Cells were trypsinised and were collected by centrifuging at 5000 rpm for 2 min at 4°C. The cell pellets were washed with 250 µl of ice-cold 1x PBS and were collected again by centrifuging at 5000 rpm for 2 min at 4°C. The cells were resuspended in 100 µl of ice-cold 1x PBS and were fixed by adding 700 µl of ice-cold 70% ethanol and incubated on ice for 45 min. The fixed cells were collected by centrifuging at 5000 rpm for 10 min at room temperature. The pellets were resuspended in 1x PBS and centrifuged again at 5000 rpm for 10 min at room temperature. The cell pellets were resuspended in 250 µl of 1x PBS, and 5 µl of 10 mg/ml RNase enzyme was added. Cells were then incubated at 37°C for one hour. To the cell suspension, 10µl of 1mg/ml propidium iodide (PI) was added and cells were filtered through a 40 µm filter to eliminate the cell clumps. The cell suspensions were kept in the dark at 4°C till the samples were run in BD

FACS Aria II. The samples were run thrice to obtain technical replicates, and 10000 cells were analyzed per run. The data was plotted into histograms using the FACS Diva software.

6) Mitotic cell index analysis:

pH3(Ser10) and Hoechst double-stained cells were imaged in 20x magnification. Fix square ROI was chosen with the help of CellSens software, and 5 fields were imaged per condition. The mitotic cells were identified with the help of pH3(Ser10) staining and counted with the help of software. The total number of cells per ROI was identified with Hoechst nuclear staining and measured in Fiji Software with the help of the Otsu tool. The mitotic cells were counted manually. The prophase, metaphase, anaphase and telophase cells were identified in each area, and the numbers were normalized to percentages.

7) Immunocytochemistry:

The 8-well chamber slide was washed with 1x PBS, and the cells were permeabilized with 0.25% saponin for 20 minutes. The saponin solution was removed entirely by washing with 1x PBS, and the blocking was done with a solution containing 5% BSA and 2% donkey serum for 1 hour at RT. The cells were incubated in respective primary antibodies in a humidified chamber for 16-18 hours at 4° Celsius. After the primary antibody incubation, the cells were washed with 1x PBS, and the respective secondary antibody was added to the cells and incubated for 1.5 hours at RT in the dark. The cells were rewashed in 1x PBS and were Hoechst stained for 5 minutes at RT. The slide was mounted in 70% Glycerol in 1x PBS with a clean coverslip. Imaging was done in Nikon A1R-si laser scanning confocal microscope.

8) RNA Isolation:

TRIzol (Invitrogen – 15596018) method was followed for the RNA isolation based on the manufacturer's recommendations. Briefly, the cells were homogenized with 1 mL TRIzol, and 200µl of Chloroform was added, followed by vigorous mixing. The samples were phase separated by centrifuging at 12000 g for 15 minutes at 4°C. The aqueous phase was separated, and an equal volume of Isopropanol was added to each sample to precipitate the RNA. After incubation of 10 minutes at RT, the samples were centrifuged at 12000g for 10 minutes at 4°C. The samples were washed twice in 75% ethanol, followed by centrifugation at 7500 g for 5 minutes each time. The RNA pellets were air-dried and re-dissolved in an appropriate volume of nuclease-free water. Samples were stored in a -80° Celsius freezer.

9) Real-Time PCR:

According to the manufacturer's protocol, the high-capacity cDNA reverse transcription kit (Thermofisher - 4368814) was used for cDNA synthesis from the RNA samples. Briefly, 1.5 µg of RNA sample was mixed with 1x cDNA synthesis reagents, and the manufacturer-recommended PCR cycle protocol was run. From the cDNA samples, 1 µl was used as the template per well in 50 µl final reaction volume for the real-time PCR and results were analyzed by the Livak method, using the delta-delta-Ct (ddCT) algorithm.

The RT-PCR primer sequences used were:

1. SENP1 Forward CATTTCGCCTGACCATTACACGC
2. SENP1 Reverse CACACTTGGCAAGCCCTTCTCT
3. SENP2 Forward CAGAGACGATGGTCGGAATCAG
4. SENP2 Reverse CCTCCTGAGTAAGCCATTGCTTC
5. SENP3 Forward ATCCACCTGGAGGTGCATTGGT
6. SENP3 Reverse TCTTTACCGCCTCTGCCTGTAG
7. SENP5 Forward GTCAGAAAGCCTCTCCAGTGGA
8. SENP5 Reverse CAAGGACTTCTTTTTCACTGAGTG
9. SENP6 Forward GAGCATCAAAGGAAGTTGTGGGC
10. SENP6 Reverse GAAGATGGTGTGGTTTTCTCCAG
11. SENP7 ForwardTCTTTCCCTGCTGGTGTTGCTG
12. SENP7 Reverse CAACCGCTACTTTGCTTCTGCAG

10) Sub-Cellular fractionation:

The cells were seeded at a confluence of $1x10^6$ cells per 100 mm dish, and the treatments were followed for 96 hours. Cells were then harvested by scraping and resuspended in Buffer A (10mM Hepes pH-7.5, 10mM KCl, 2mM MgCl2, 1mM EDTA, 0.2% NP-40, 20mM NEM) containing 1x protease inhibitors with further incubation on ice for 30 min. The cell suspension was passaged 30 times using a 1 ml syringe with a 21G size needle, and 2 µl of the cell suspension was mixed with trypan blue to visualize the release of intact nuclei under the microscope. When almost 80-90% of the cells had released the nucleus, the suspension was centrifuged at 5000 rpm for 5 minutes at 4°C, and the supernatant was collected and labeled as the cytoplasmic fraction. After washing the nuclear pellet once with 1 mL of buffer A, the nuclei were again pelleted down by centrifugation at 500 g for 10 minutes at 4°C. The

nuclear pellets were homogenized by adding 100 µl of Buffer A++ (Buffer A supplemented with 500 mM NaCl and 25% glycerol) and incubating for 30 minutes on the tube rotator at 4°C. The nuclear pellets were sonicated with 5 seconds on – 10 seconds off cycle for 5 minutes at an amplitude of 37 to shear the dense chromatin in the nuclear lysate. The samples were then centrifuged at 15000 g for 15 minutes at 4°C, and the supernatant was collected and labeled as the nuclear fraction. All samples were stored at -20 for the short term and -80 for the long term.

11) Co-immunoprecipitation:

CaSki/C-33 A cells were harvested by scraping, and sub-cellular fractionation was performed to separate cytoplasmic (CF) and nuclear (NF) fractions. Each CF and NF sample (1000 µg protein) was made up to 1 mL with IP buffer (10 mM Hepes pH-7.5, 10 mM KCl, 2 mM MgCl2, 1mM EDTA, 0.2% NP-40, 20 mM NEM and 1x protease inhibitor cocktail). Each sample was incubated with 15 µl (3 µg) of anti-SUMO2 antibody (sc-26972) for 10 min at RT with gentle rotation. Dynabeads were used as per the manufacturer's instructions for immunoprecipitation. Briefly, 1mg of Dynabeads was added to each sample and was incubated for 1 hour at RT with gentle rotation. The Dynabead-antigen-antibody complex was eluted with 20 µl of elution buffer and 4 µl of 5x sample loading dye by heating the beads at 95°C for 5 minutes. The IP samples were loaded and separated by 4–20% SDS-PAGE, transferred to PVDF membrane, and blotted with anti-pH3(Ser10) (Abcam-ab267372) antibody (1:1,000) followed by peroxidase-conjugated affinity pure donkey anti-rabbit secondary antibody (Jackson Immuno Research, 1:10,000). Protein bands were visualized with ECL in Azure C600 Imaging System and were analyzed using Fiji.

12) MTT assay:

MTT ([3-(4,5-dimethylthiazole-2-yl)-2,5-diphenyltetrazolium bromide) assay was performed to analyze the cell viability after drug treatments. The normal cells-HaCaT (human keratinocytes), SVG (human astrocytes) and H9C2 (rat cardiomyoblasts) were seeded in 96-well plates (10000 cells/well) and post 24 hours of incubation; they were treated with different concentrations of Momordin Ic (10 µM, 25 µM, 50 µM, 100 µM and 200 µM) and Gallic Acid (individually or in combination). Followed by the drug treatment, the cells were re-fed with fresh media containing MTT solution at a final concentration of 400 µg/mL and were incubated for 2 hours. The media with MTT solution was discarded once the incubation time was over, and the formazan crystals were dissolved by adding 100 µl of DMSO per

well. Absorbance was measured with the Varioskan multimode reader at 490 nm wavelength. Absorbance normalization was done with plain DMSO, and the relative cell viability in percentage was calculated as (Normalized absorbance of treated samples/ Normalized absorbance of untreated samples) x 100.

13) siRNA transfection:

Transient transfection of the cervical cancer cell lines was performed with Jetprime (Polyplus) for cell proliferation recovery analysis and immunostaining, according to the manufacturer's recommendations. Briefly, the cells were cultured for 24 hours in MEM media supplemented with 10% (vol/vol) fetal bovine serum and antibiotic cocktail, followed by transfection at ~70-80% confluence. In the transfection buffer, 100 nM of SUMO2/3 siRNA (Santacruz Biotechnology, sc-37167)) was diluted, followed by the addition of Jetprime reagent. This siRNA-Jetprime mix was added to the cells for 36 hrs, and the 75-80% knockdown was confirmed by immunofluorescence staining and imaging. Post knockdown, the NG, HG, and HG-HO treatments were given along with normoxia or hypoxia for 72 hrs. Cells were then fixed for immunostaining or were progressed for estimation of proliferation per condition by Hoechst staining, imaging and cell count analysis through Fiji image analysis software.

14) Docking and MD simulations of pH3(Ser10) with SUMO variants:

Histone H3 present within the X-ray structure of the nucleosome core particle (PDB 1kx5) and SUMO2 (PDB 5d2m chain B) was used to generate the docking models by ZDOCK v3.0.2 program with the default parameters [R. Chen, L. Li, and Z. Weng, "ZDOCK: An initial-stage protein-docking algorithm," Proteins Struct. Funct. Genet., vol. 52, no. 1, pp. 80–87, Jul. 2003, doi: 10.1002/prot.10389]. The whole nucleosome core was used in the docking analysis, and the N-terminal tail region of Histone H3 (1kx5_A chain) was targeted for docking. The best ZDOCK score model was selected as the final one. The atomic coordinates of SUMO2 and Histone H3 tail (130 amino acids) were copied as separate structure complexes from the final docked model. Further, the SUMO2-Histone H3 tail complex obtained was subjected to Molecular Dynamics (MD) simulations using GROMACS v4.5.5 [B. Hess, C. Kutzner, D. van der Spoel, and E. Lindahl, "GROMACS 4: Algorithms for Highly Efficient, Load-Balanced, and Scalable Molecular Simulation," J. Chem. Theory Comput., vol. 4, no. 3, pp. 435–447, Mar. 2008, doi: 10.1021/ct700301q]. The complex was solvated with TIP3P water. AMBER FF99SB force field was employed to carry out the

calculations. The solvated system was subjected to 500 steps of energy minimization employing the steepest descent algorithm. 1000 ps of position restrained simulation followed this to equilibrate the water and ions. The production runs for 5 ns using 2 femtoseconds (fs) timestep in NPT ensemble at 300K and 1 atmospheric pressure controlled by Vrescale thermostat and Parrinello Rahman barostat, respectively, was carried out. LINCS algorithm [B. Hess, H. Bekker, H. J. C. Berendsen, and J. G. E. M. Fraaije, "LINCS: A linear constraint solver for molecular simulations," *J. Comput. Chem.*, vol. 18, no. 12, pp. 1463–1472, Sep. 1997, doi: 10.1002/(SICI)1096-987X(199709), 18:12<1463::AID-JCC4>3.0.CO;2-H] was used to constrain hydrogen atoms' bond lengths. The Particle Mesh Ewald (PME) was employed to calculate the electrostatic interaction with the cut-off of 10 Å. MD analyses were carried out using GROMACS analysis tools. For the complex visualizations, the PyMol package [DeLano, Warren L. "The PyMOL molecular graphics system." (2002)] was used.

15) Protein Preparation:

SUMO2 and Histone H3 with PDB codes 2N1W and 5BS7 were selected to analyze their protein-protein interaction. Histone H3.1 contains information on the two binding residues Ser10 and Ser28, selected explicitly for phosphorylation activity to understand their binding affinities with SUMO2. The processes for assigning binding orders that add hydrogen, treat metals, treat disulfides, remove water, and reduce potential steric collisions were performed by standard protein preparation protocol within Maestro to prepare the receptors for modelling [Schrödinger Release 2020: Maestro, Schrödinger, LLC, New York]. The assignment of bond order, formal charges, construction of the missing heavy atom and decreasing the energy was achieved with the OPLS3 force field with a 0.3 Å RMSD limit imposed as a constraint on the refinement of the proteins.

16) Protein-Protein Interaction:

The best allosteric site binding interaction of SUMO2 with Histone H3 was investigated by the docking server Cluspro [D. Kozakov et al., "The ClusPro web server for protein–protein docking," Nat. Protoc., vol. 12, no. 2, pp. 255–278, Feb. 2017, doi: 10.1038/nprot.2016.169] to achieve a set of possible complexes. The complex with Histone H3 near the allosteric site was chosen since it is necessary to find the importance of phosphorylation of the potential interacting serine residue in the allosteric region. The Ser10 and Ser28 were modified to phosphorylated Serine in the SUMO2-H3 complex using the 3D builder in maestro to create the following complexes (SUMO2_Ser10/H3, SUMO2_Ser28/H3, SUMO_Ser10+28/H3) to

understand the functional consequence of Histone H3 phosphorylation-dependent SUMOylation dynamics.

17) Molecular Dynamic Simulations:

Three phosphorylated SUMO2-H3 complexes were submitted for molecular dynamics (MD) simulation analysis using Desmond [Bowers et al., “Scalable Algorithms for Molecular Dynamics Simulations on Commodity Clusters,” in ACM/IEEE SC 2006 Conference (SC’06), Tampa, FL, Nov. 2006, pp. 43–43. doi: 10.1109/SC.2006.54]. The OPLS3 force fields were used to build aqueous biological systems, and the TIP3P water model was used to soak molecules and was further adjusted to minimize volume. The osmotic effect of water was mimicked in a 15 Å buffered box with orthorhombic periodic boundary conditions added by 0.15 mol /L sodium and chloride ions. The following settings were used to submit each system to the MD simulation: a simulation time of 100 ns, trajectory recording intervals of 20 ps and energy recordings of 5 ps. A constant temperature of NPT at 300 K was maintained throughout the simulation using the Nose-Hoover thermostat algorithm, and retaining 1 atm of pressure was done with the Martyna-Tobias-Klein Barostat algorithm, respectively with a relaxation time of 100 ps.

Supplementary Section S2 – Figures with Legends

SUPPLEMENTARY FIGURES WITH LEGENDS:

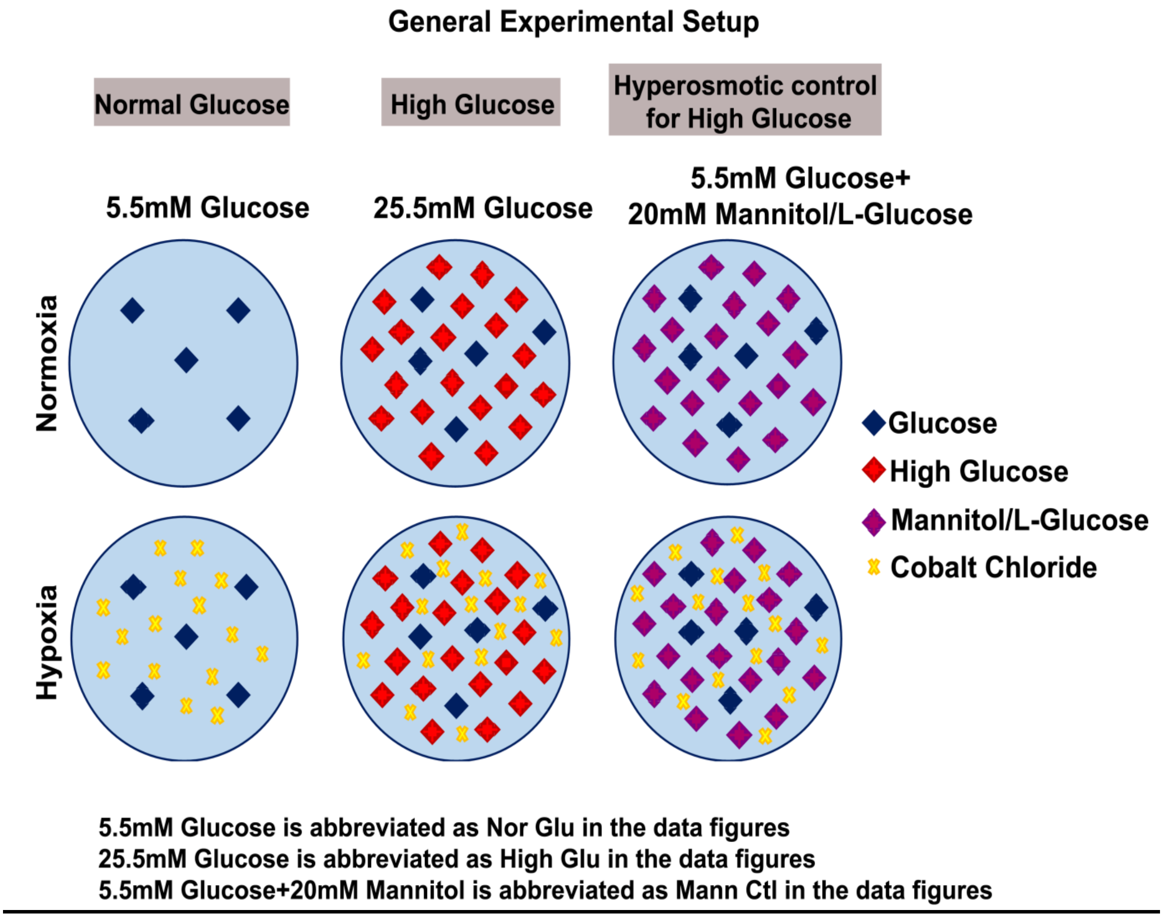

Supplementary Figure S1: General experimental set-up for all experiments.

The control group was given physiological levels of 5.5 mM glucose (NG or Nor Glu). The high glucose (HG or High Glu) and hyperosmotic equivalents (HGHO or L-glucose Ctl or Mann Glu Ctl) were generated by adding 20 mM of glucose or L-Glucose/mannitol, respectively, to baseline physiological glucose levels of 5.5 mM. The basal 5.5 mM glucose (NG) was not omitted from the hyperosmotic equivalents since that might affect the normal glucose signalling. Hypoxia was generated by adding 150 µM $CoCl_2$ to the conditions mentioned above. 150 µM $CoCl_2$ was kept in the media throughout the treatment conditions.

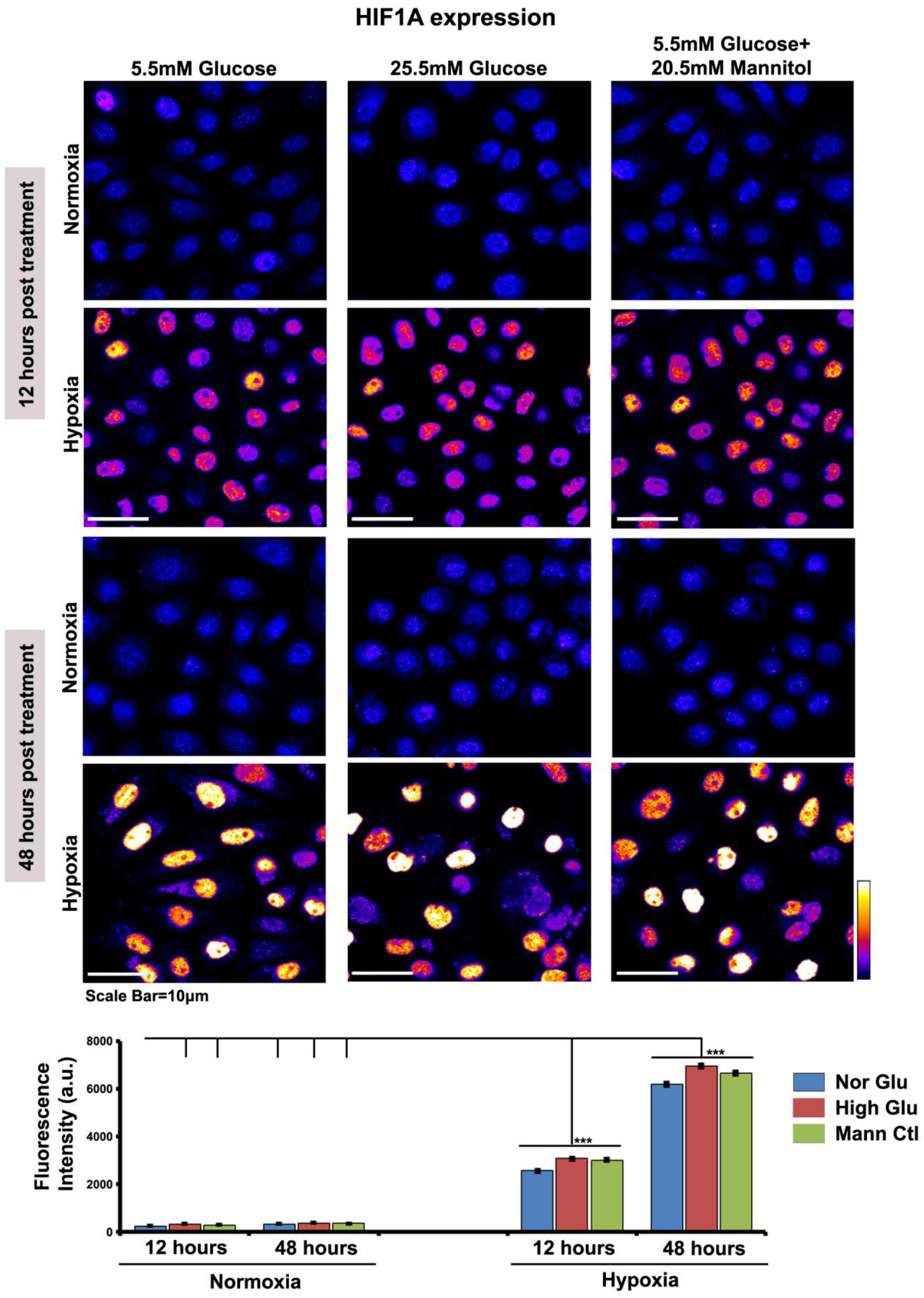

Supplementary Figure S2: HIF1a, a known hypoxia marker, showed stable expression by 48 hours of treatment with 150 µM Cobalt chloride in the tumour cell culture medium.

Chemical hypoxia was induced by adding 150 µM $CoCl_2$ in the CaSki cervical cancer cell culture medium. The confocal images showed an increase in the hypoxia marker, HIF1a, by 12 hours and stabilized higher expressions of the same by 48 hours (graph). Low levels of HIF1a were observed in normoxic conditions. This is expected as cancer cells tune to aerobic glycolysis and upregulate HIF1a at lower levels. Also, several growth factors upregulate HIF1a at lower levels, independent of hypoxia. 5 random fields were imaged per condition, and fluorescence intensity analysis was performed in 3 independent conditions. At least 30 cells in each condition were analyzed. Image acquisition parameters for each channel were kept the same across each condition and over independent replicates. Statistical significance is represented by p values: $*p \leq 0.05$, $**p \leq 0.01$ and $***p \leq 0.001$.

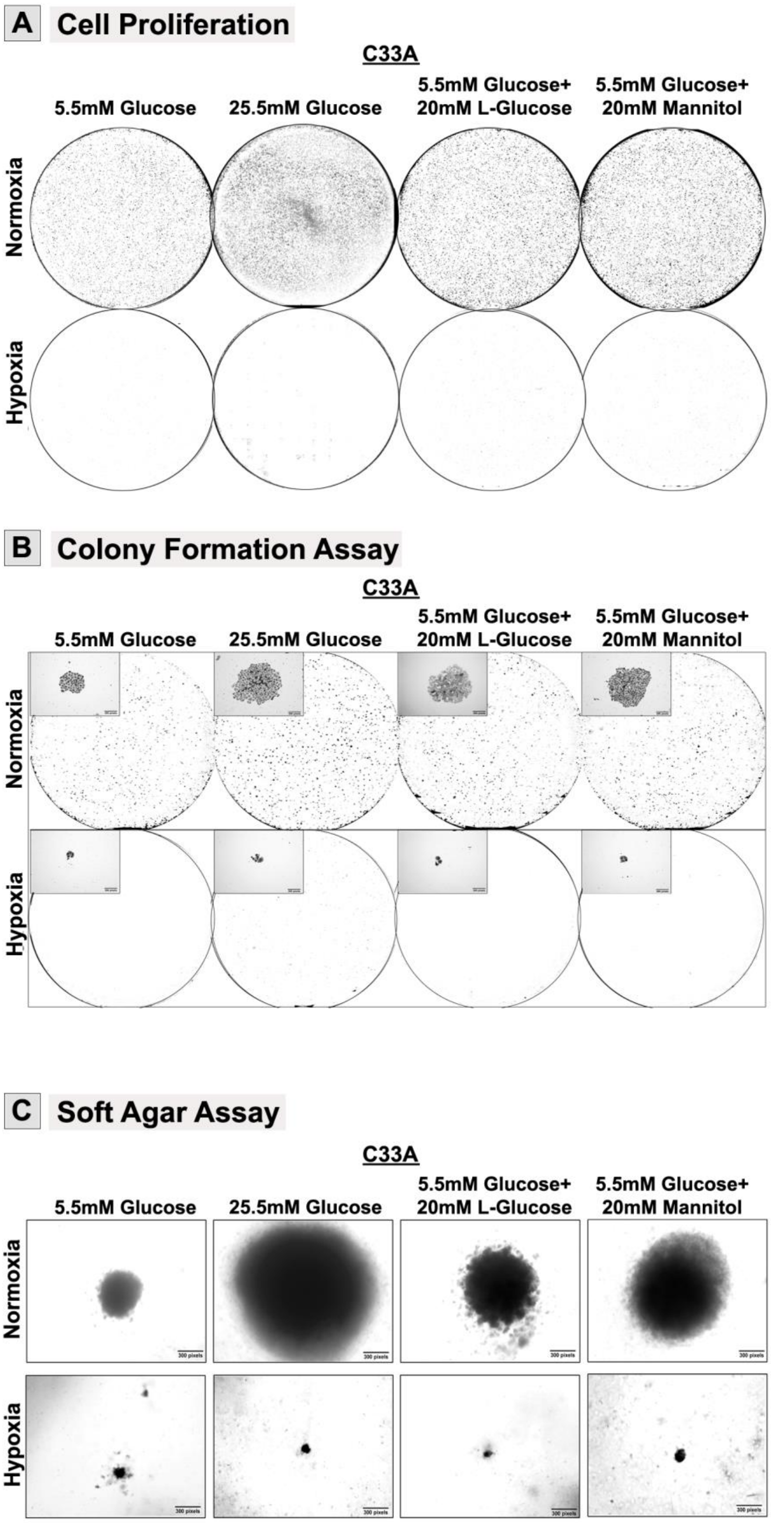

Supplementary Figure S3: Representative images of confluency, colony formation and soft agar assay in C-33A cervical cancer cells. This figure is associated with the main FIGURES 1C, 1E, and FIGURE 2A.

(A) C-33A cervical cancer cells were cultured either NG HG-HO with or without hypoxia for 96 hours. Microscope- based motorised stitching of the wells, in 4X magnification, was done

for image representations and analysis of cell proliferation. **(B)** C-33A cells were exposed to the treatment conditions for 12 days till the colony forming units were formed in control well. Motorised stitching of the wells in 4x magnification was done for image representations, and one representative colony image is shown in the inset. **(C)** C-33A cells were subjected to the basic experimental conditions in a soft agarose substrate for 21 days. Representative spheroid image in each condition is shown. Image acquisition parameters for each channel were kept the same across each condition and over independent replicates

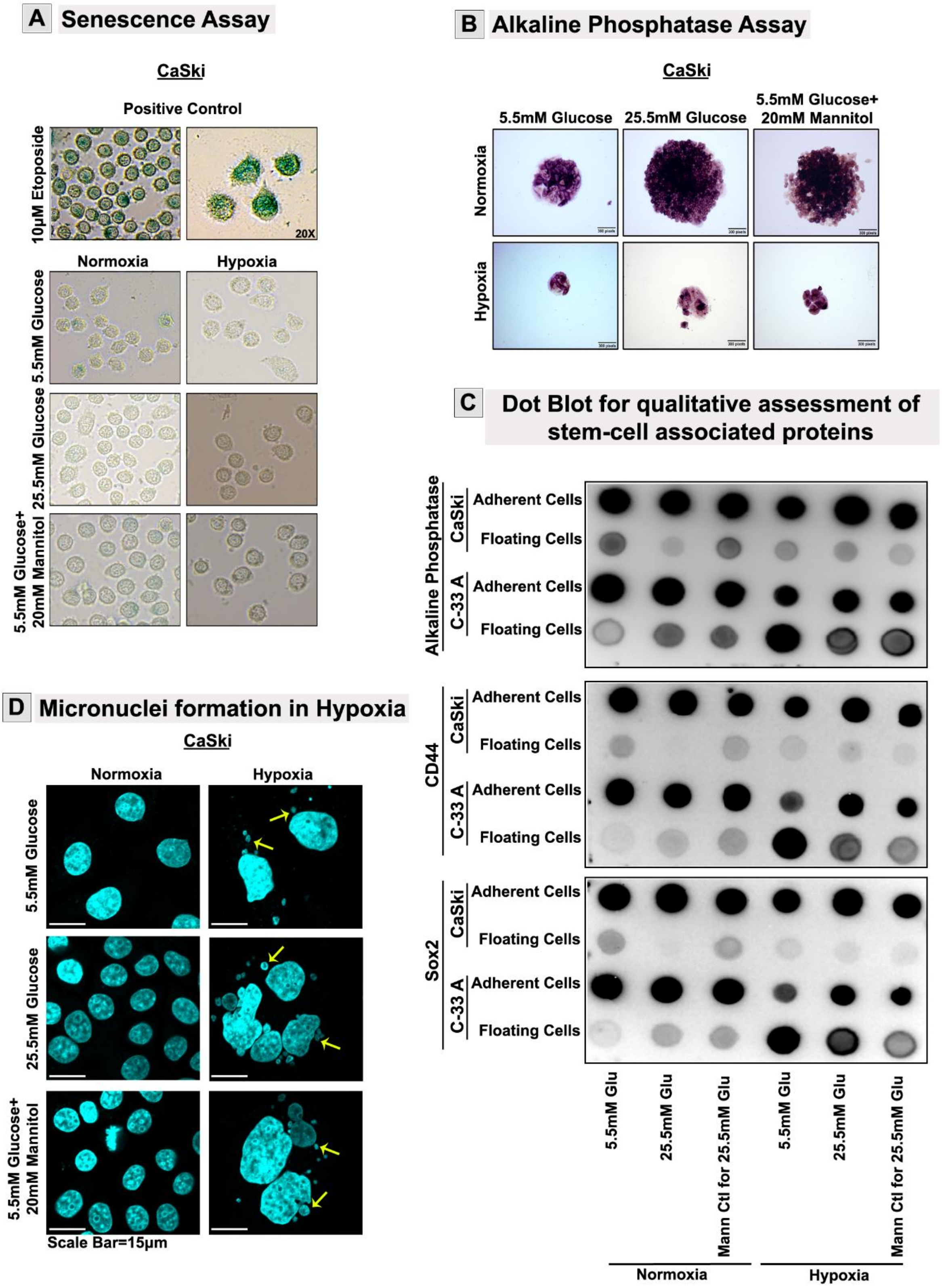

Supplementary Figure S4: Cell fate analysis of tumour cells in normal, high glucose and hyperosmotic equivalent conditions under normoxia and hypoxia.

(A) CaSki/C33A cervical cancer cells were cultured in the general experimental set-up for 96 hours and probed for senescence associated with SA-β-galactosidase by the X-gal method.

The images indicate that loss of cells in hypoxia was independent of senescence induction, as there was no significant difference in the staining of all conditions **(B)** CaSki/C33A cancer cells were cultured in the general experimental set-up for 12 days, till colony formation units (CFU) were formed in the dish. BCIP/NBT-based chromogenic detection of alkaline phosphatase showed residency of stem-like cells in all conditions compared NG normoxia control. **(C)** CaSki/C-33A cancer cells were exposed to the general experimental conditions for 96 hours. The floating cells and the adherent cell lysates were qualitatively probed for stemness markers-Alkaline phosphatase, CD44 and SOX2 via the dot blot assay. The dot blots showed the presence of stemness markers in both adherent and floating cell populations in tumour cell lines. **(D)** Confocal imaging of Hoechst-stained CaSki/C33A tumour cells showed the presence of micronuclei in hypoxia, indicative of abnormalities in the cell cycle and mitotic chromosome segregation. Yellow arrows in the images indicate the acute hypoxia-induced micronuclei. Image acquisition parameters for each channel were kept the same across each condition and over independent replicates.

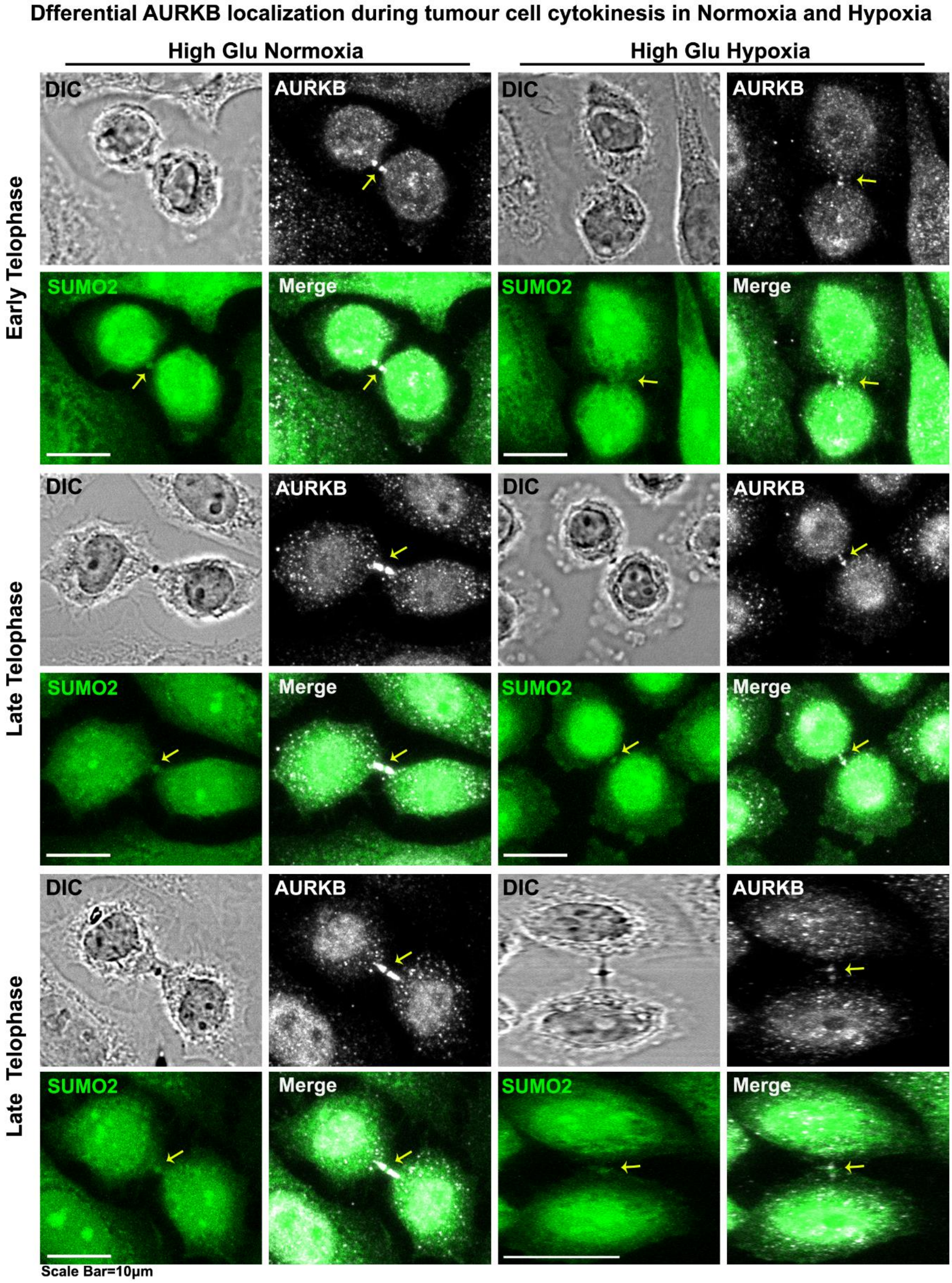

Supplementary Figure S5: AURKB localization to the midbody was less in acute hypoxia-exposed tumour cells.

CaSki cervical cancer cells were cultured in the general experimental set-up for 96 hours and were immunostained with AURKB and SUMO2. The images reveal a reduced mid-body

localization of crucial mitosis-associated kinase, AURKB, throughout telophase's early and late stages, which may account for stalled cytokinesis (cell division arrest) in the cells exposed to acute hypoxia. Yellow arrows indicate the localization of AURKB at the midbody region. Image acquisition parameters for each channel were kept the same across each condition and over independent replicates.

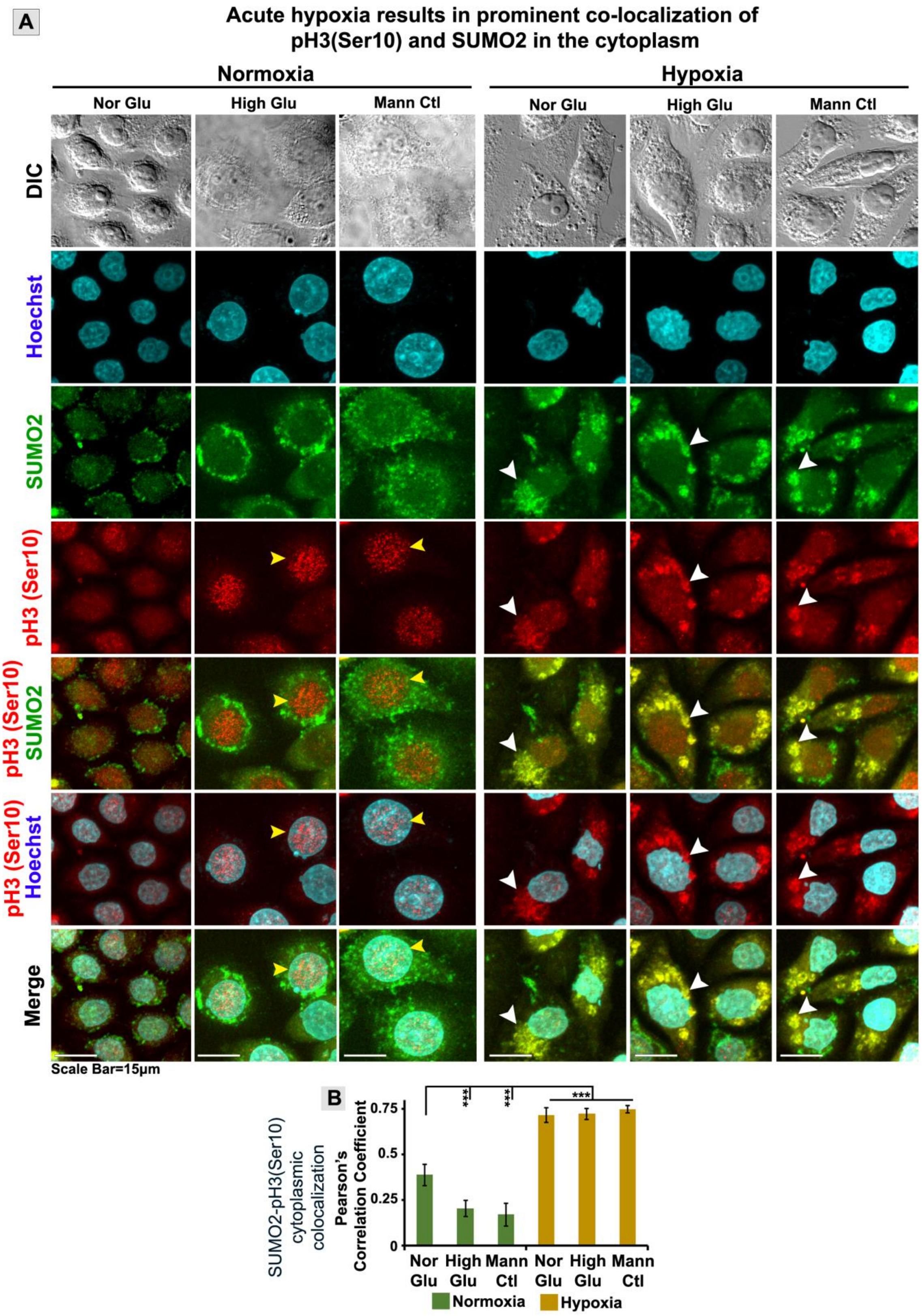

Supplementary Figure S6: pH3(Ser10) is prominently colocalized with SUMO2 in the cytoplasm of acute hypoxia-treated cancer cells in contrast to a nuclear colocalization in normoxia.

CaSki cervical cancer cells were cultured in the general experimental set-up for 96 hours and were immunostained with pH3(Ser10) and SUMO2.**A)** The confocal images showed robust cytoplasmic colocalization of these two proteins in acute hypoxia-treated cells. In contrast, the key mitotic protein pH3(Ser10) was observed to be localized in the nucleus in HG/HG-HO in normoxia. White arrowheads indicate the cytoplasmic colocalization, and the yellow arrowheads point at the nuclear localization of pH3(Ser10).Image acquisition parameters for each channel were kept the same across each condition and over independent replicates. **(B)** Pearson's correlation coefficient shows that the extent of cytoplasmic co-localization of SUMO2-pH3(Ser10) is very high compared to the normoxic conditions. Image acquisition parameters for each channel were kept the same across each condition and over independent replicates. Statistical significance is represented by p values: $*p \leq 0.05$, $**p \leq 0.01$ and $***p \leq 0.001$.

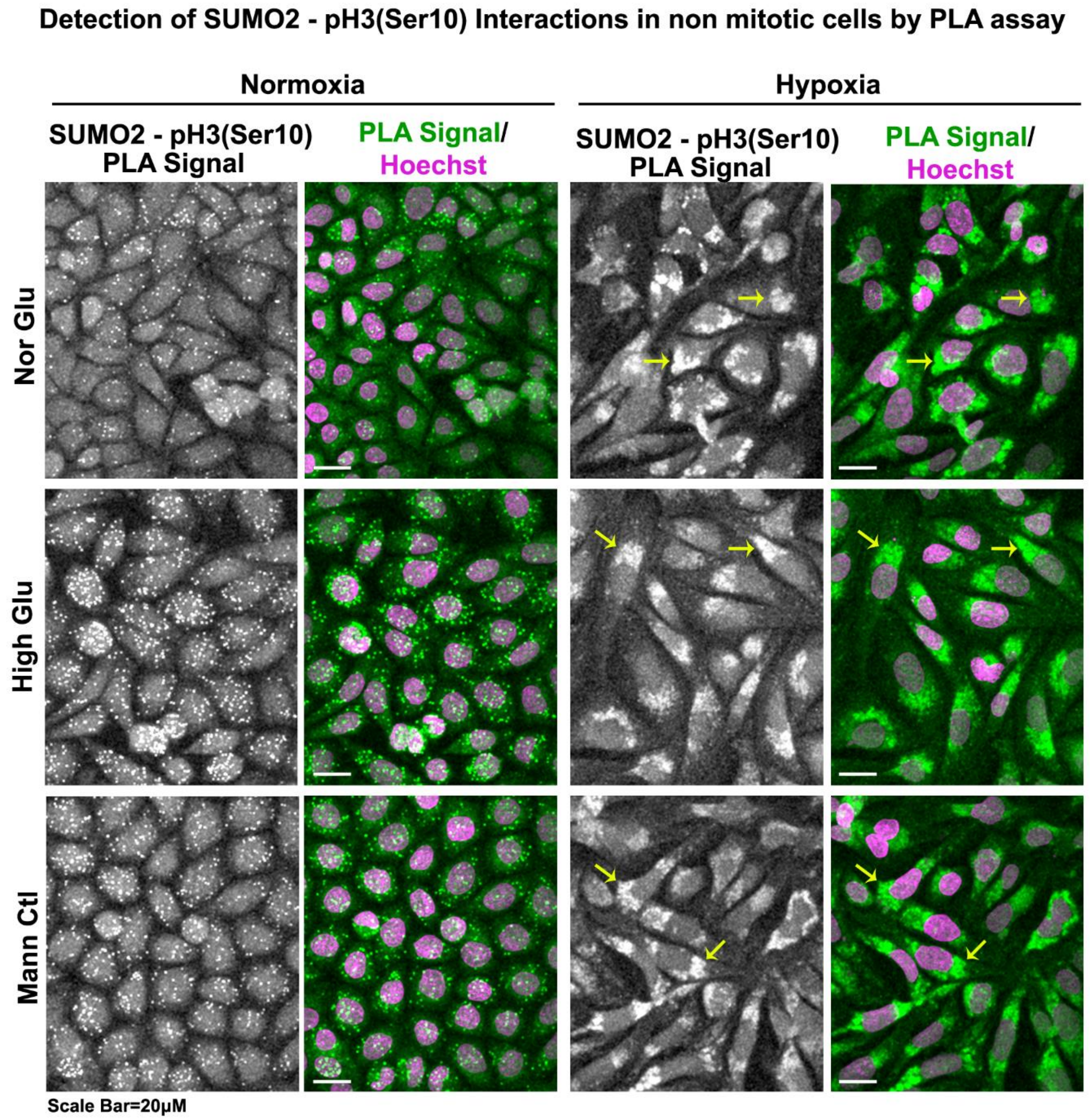

Supplementary Figure S7: Protein-Protein interaction detection assay, the proximity ligation assay (PLA), showed a cytoplasmic signal of pH3(Ser10) and SUMO2 in acute hypoxia treated cancer cells, irrespective of high glucose/hyperosmotic conditions.

CaSki cervical cancer cells were cultured in the general experimental set-up for 96 hours. Detection of interaction between SUMO2 and pH3(Ser10) in CaSki tumour cells revealed a prominent cytoplasmic signal in hypoxic conditions. In contrast, enhanced nuclear localization of the same was observed in HG and HG-HO conditions in normoxia. Yellow arrowheads indicate the cytoplasmic PLA signal, suggesting that SUMOylated pH3(Ser10) was sequestered in cytoplasm as aggregates. Image acquisition parameters for each channel were kept the same across each condition and over independent replicates.

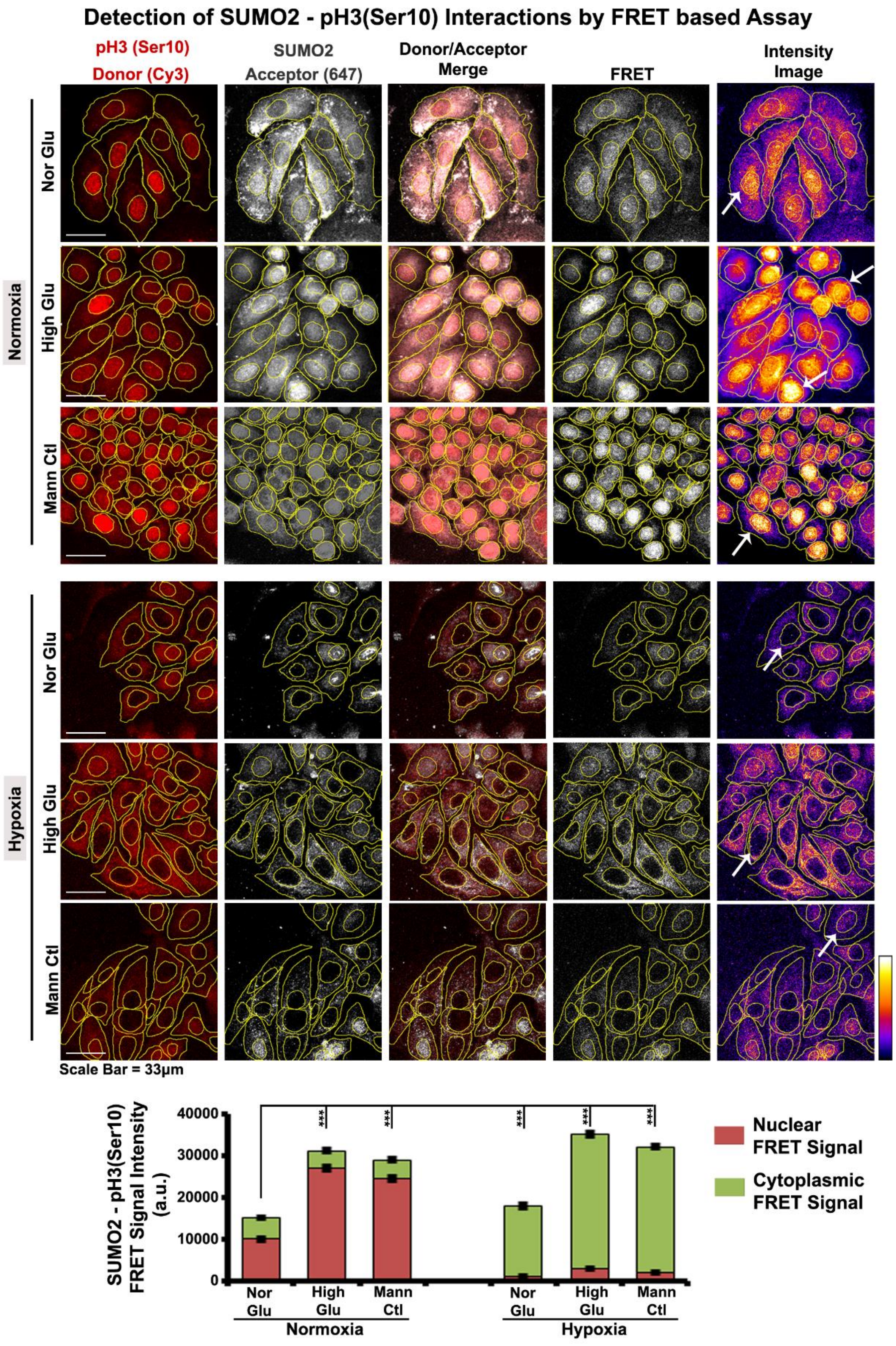

Supplementary Figure S8: Direct FRET between pH3(Ser10) and SUMO2 confirms cytoplasmic sequestration of SUMOylated pH3(Ser10) in acute hypoxia.

CaSki cervical cancer cells were cultured in the general experimental set-up for 96 hours. They were immunostained with pH3(Ser10) and SUMO2 primary antibodies, which were

immunolabeled with secondary antibodies conjugated with Cy3 and Alexaflour 647, respectively. Cy3 and Alexaflour 647 are established FRET donor-acceptor pairs. Higher nuclear vs cytoplasmic FRET signal between pH3(Ser10) and SUMO2 were noticed in normoxic HG/HG-HO treatments. In contrast, the cytoplasmic FRET signal was prominent in acute hypoxia-induced conditions, irrespective of the HG or HG-HO state. White arrows indicate the cytoplasmic FRET signals between SUMO2 and pH3(Ser10). At least 30 cells in each condition were analyzed. Image acquisition parameters for each channel were kept the same across each condition and over independent replicates. Statistical significance is represented by p values: $^{*}p \leq 0.05$, $^{**}p \leq 0.01$ and $^{***}p \leq 0.001$.

Consolidated information of the tissue array is presented in the table below; the grades are classified according to WHO 2013 edition

Uterine cervicl cancer survey tissue array, including pathological grade, TNM, clinical stage and IHC marker (P16 and HPV), 104 cases/208 scores. 104 cases of malignant cervical tumour includes 87 squamous cell carcinoma, 3 adenosquamous carcinoma, 14 adenocarcinoma.

The specification data sheet of CR2087 tissue array can be obtained from: https://www.tissuearray.com/tissue-arrays/Cervix/CR2087

Human Tumor Tissue Array	Grade	Stage	Total No. of Samples	No. of HPV Positive Samples	No. of HPV Negative Samples	Remarks
Grade 1 Patients = 7	1	I	2	1	-	Cervical Squamous Cell Carcinoma
	1	IIA	2	1	-	Cervical Squamous Cell Carcinoma
	1	IB	2	-	2	Cervical Squamous Cell Carcinoma
	1	IIB	1	-	1	Cervical Squamous Cell Carcinoma
Grade 2 Patients = 62	2	I	11	8	3	Cervical Squamous Cell Carcinoma
	2	IA	2	1	1	Cervical Squamous Cell Carcinoma
	2	IB	18	10	8	Cervical Squamous Cell Carcinoma
	2	II	2	1	1	Cervical Squamous Cell Carcinoma
	2	IIA	13	8	5	Cervical Squamous Cell Carcinoma
	2	IIB	11	10	1	Cervical Squamous Cell Carcinoma
	2	III	1	-	1	Cervical Squamous Cell Carcinoma
	2	IIIB	3	1	2	Cervical Squamous Cell Carcinoma
Grade 3 Patients = 24	3	I	5	4	1	One sample Uterine Adenocarcinoma
	3	IB	6	4	2	Two samples Uterine Adenocarcinoma
	3	II	1	1	-	Cervical Squamous Cell Carcinoma
	3	IIA	6	3	3	One sample Uterine Adenocarcinoma
	3	IIB	4	2	2	Cervical Squamous Cell Carcinoma
	3	IIIB	2	1	1	Cervical Squamous Cell Carcinoma

SUPPLEMENTARY FIGURE 9

Supplementary Figure S9: The details of the USBiomax uterine cervical cancer tissue array used to generate data are shown in main FIGURE 7 and Supplementary figures 10, 11,12,13,14 and 15.

Details of clinical samples, including grades and stages of the tumour with both HPV positive and HPV negative status, are presented in a tabular format. The specification data sheet of the

cervical tumour patient tissue array can be obtained from the link https://www.tissuearray.com/tissue-arrays/Cervix/CR2087.

.

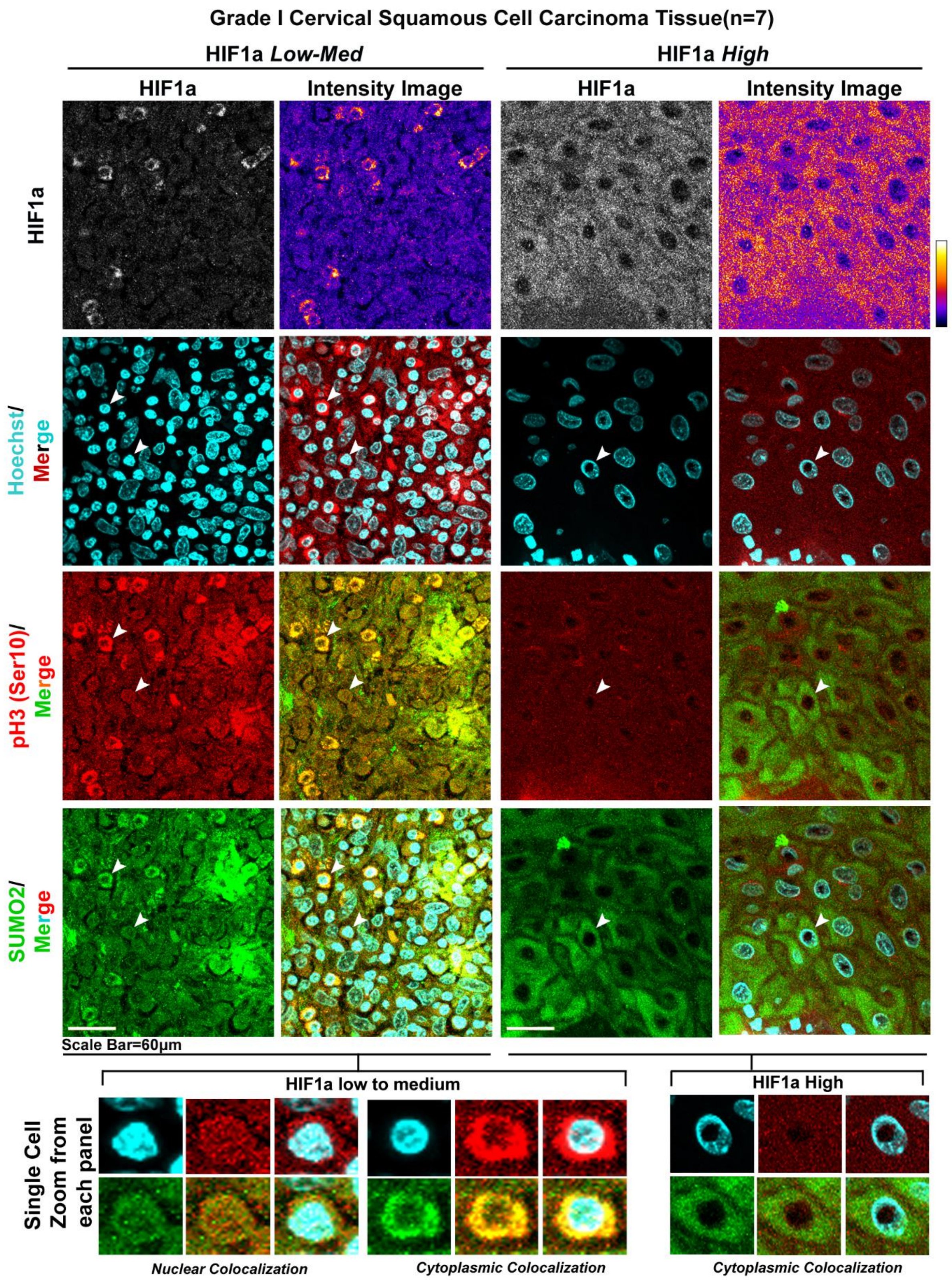

Supplementary Figure S10: Cytoplasmic colocalization of pH3(Ser10) and SUMO2 is observed in HIF1a high regions of grade I cervical squamous cell carcinoma tissues.

Grade I cervical squamous cell carcinoma patient tissues showed hypoxic (HIF1a high) and normoxic (HIF1a low-medium) areas within the same block. Cytoplasmic localization of

pH3(Ser10) and SUMO2 was observed in HIF1a antibody-positive cells. On contrary, HIF1a low to medium expressing regions showed appreciable nuclear colocalization. White arrowheads indicate the single cell with zoomed images in the lowermost panel. Image acquisition parameters for each channel were kept the same across each condition and over independent replicates.

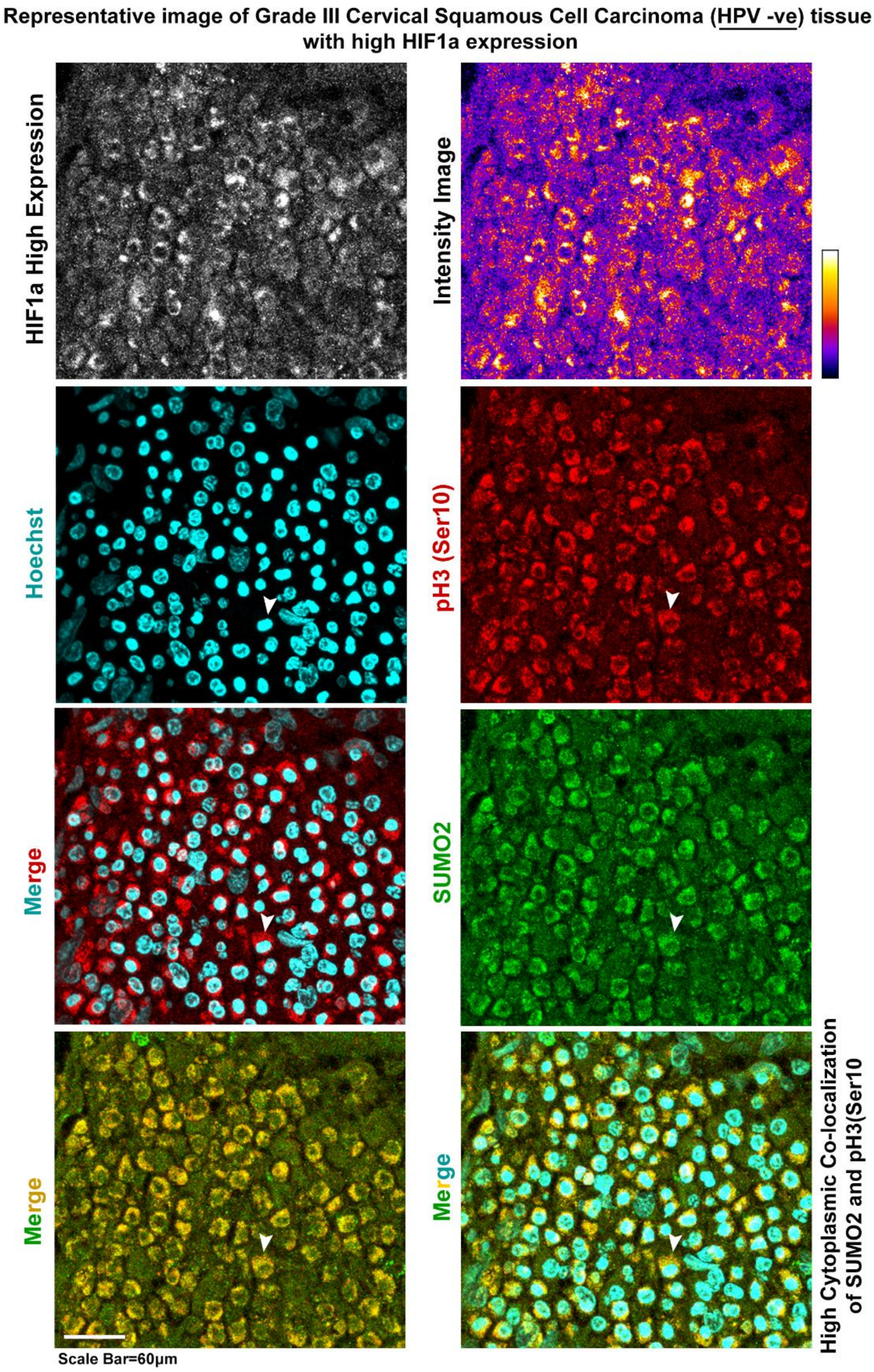

Supplementary Figure S11: Significant cytoplasmic colocalization of pH3(Ser10) and SUMO2 is observed in HIF1a high regions of grade III cervical squamous cell carcinoma tissues, which are negative for the HPV16 .

Grade III cervical squamous cell carcinoma (HPV negative) patient tissues showed hypoxic and normoxic areas within the same block. Significant cytoplasmic localization of

pH3(Ser10) and SUMO2 was observed in hypoxic (HIF1a high) areas probed with HIF1a antibody (white arrowheads indicate the described observation). Image acquisition parameters for each channel were kept the same across each condition and over independent replicates.

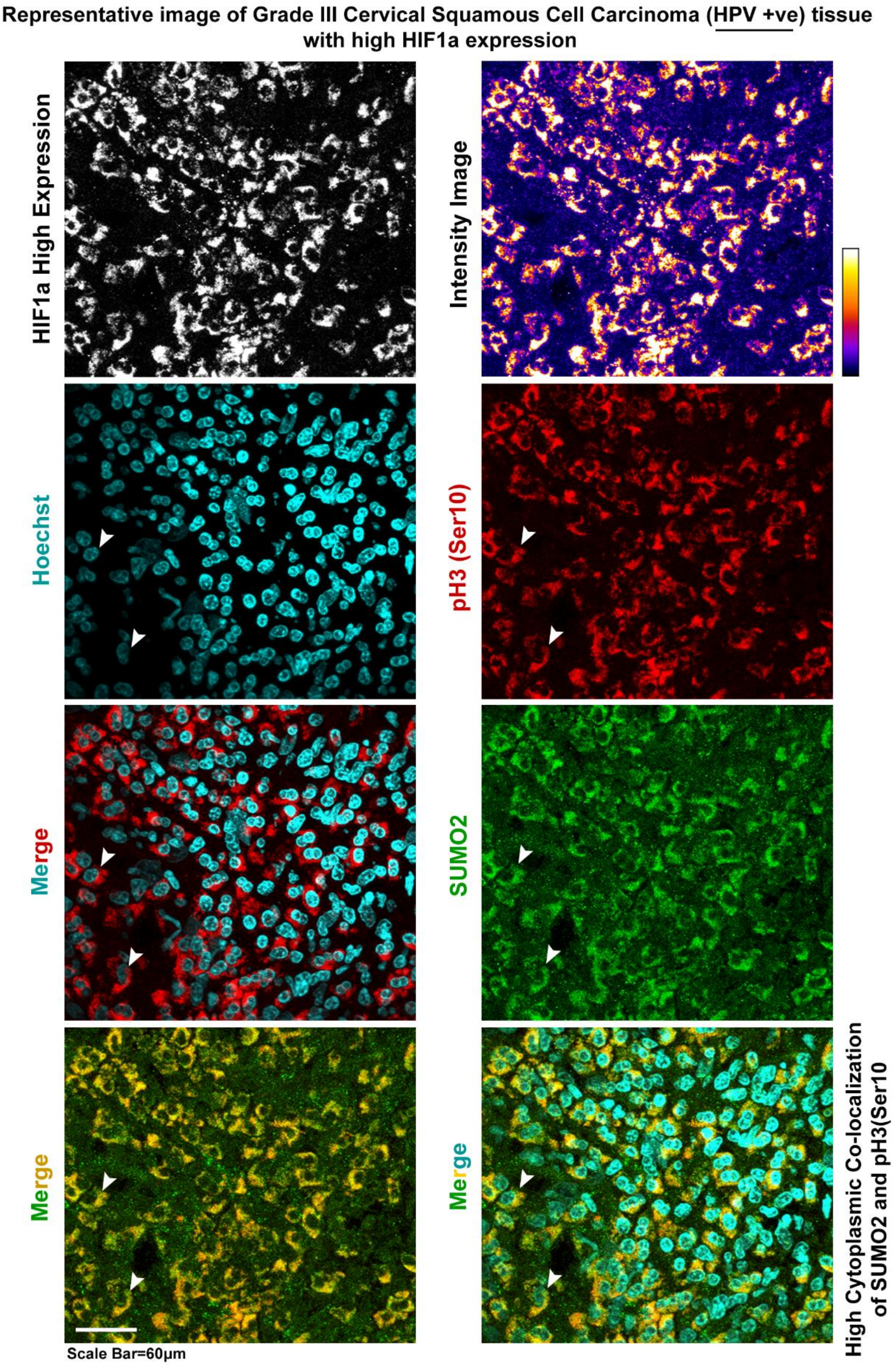

Supplementary Figure S12: Significant cytoplasmic colocalization of pH3(Ser10) and SUMO2 is observed in HIF1a high regions of grade III cervical squamous cell carcinoma tissues, which are also positive for HPV16 .

Grade III cervical squamous cell carcinoma (HPV positive) patient tissues showed both hypoxic (HIF1a high) and normoxic (HIF1a low-medium) areas within the same block.

Significant cytoplasmic localization of pH3(Ser10) and SUMO2 was observed in hypoxic areas probed with HIF1a antibody (white arrowheads indicate the described observation). Image acquisition parameters for each channel were kept the same across each condition and over independent replicates.

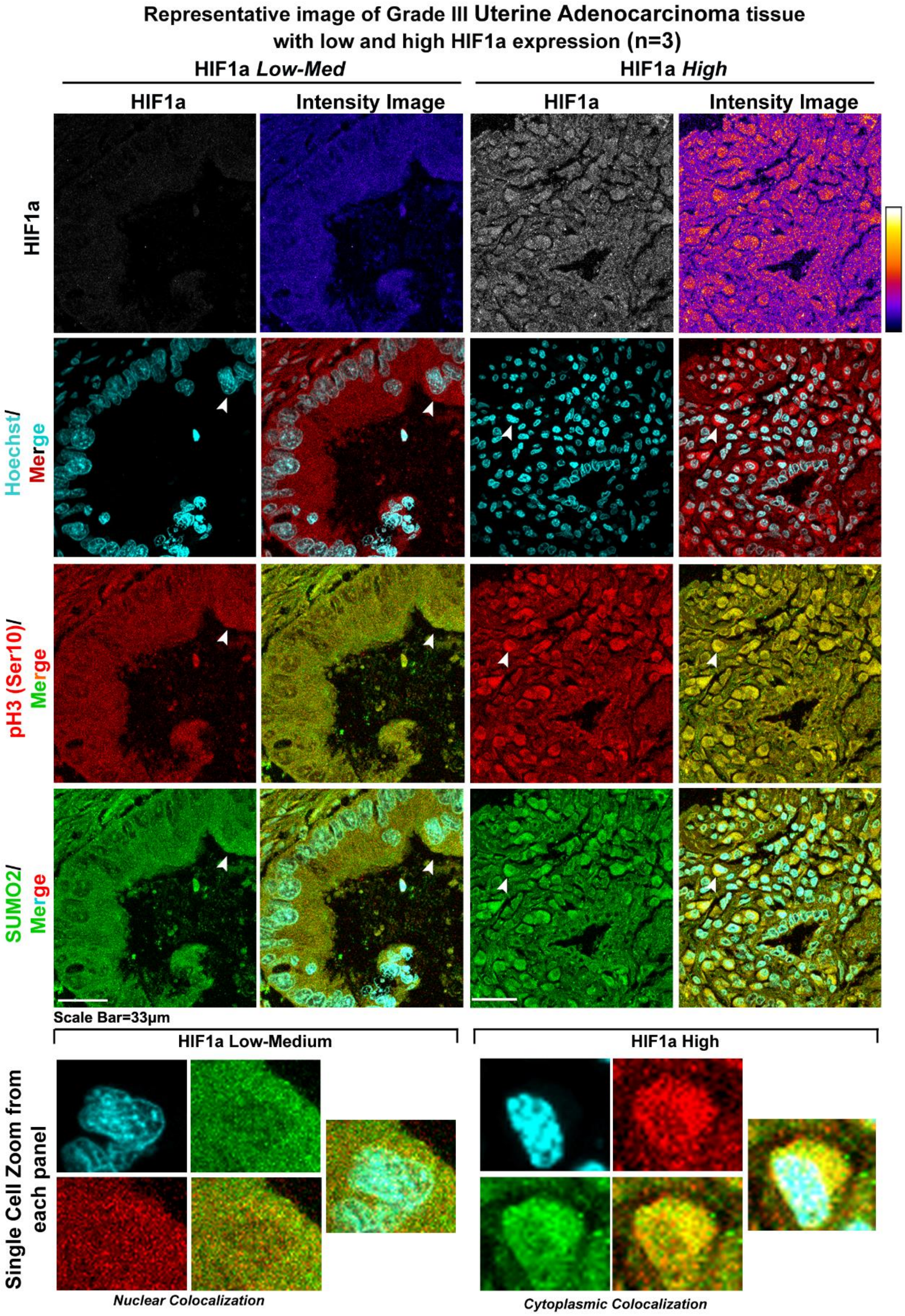

Supplementary Figure S13: Cytoplasmic colocalization of pH3(Ser10) and SUMO2 is observed in HIF1a high regions of Grade III uterine adenocarcinoma patient tissues.

Grade III uterine adenocarcinoma patient tissues showed hypoxic (HIF1a high) and normoxic (HIF1al low-medium) areas within the same block. Cytoplasmic localization of pH3(Ser10) and SUMO2 was observed with the HIF1a antibody. On the contrary, HIF1a low to medium expressing regions showed appreciable nuclear colocalization. White arrowheads indicate the single cell with zoomed images in the lowermost panel. Image acquisition parameters for each channel were kept the same across each condition and over independent replicates.

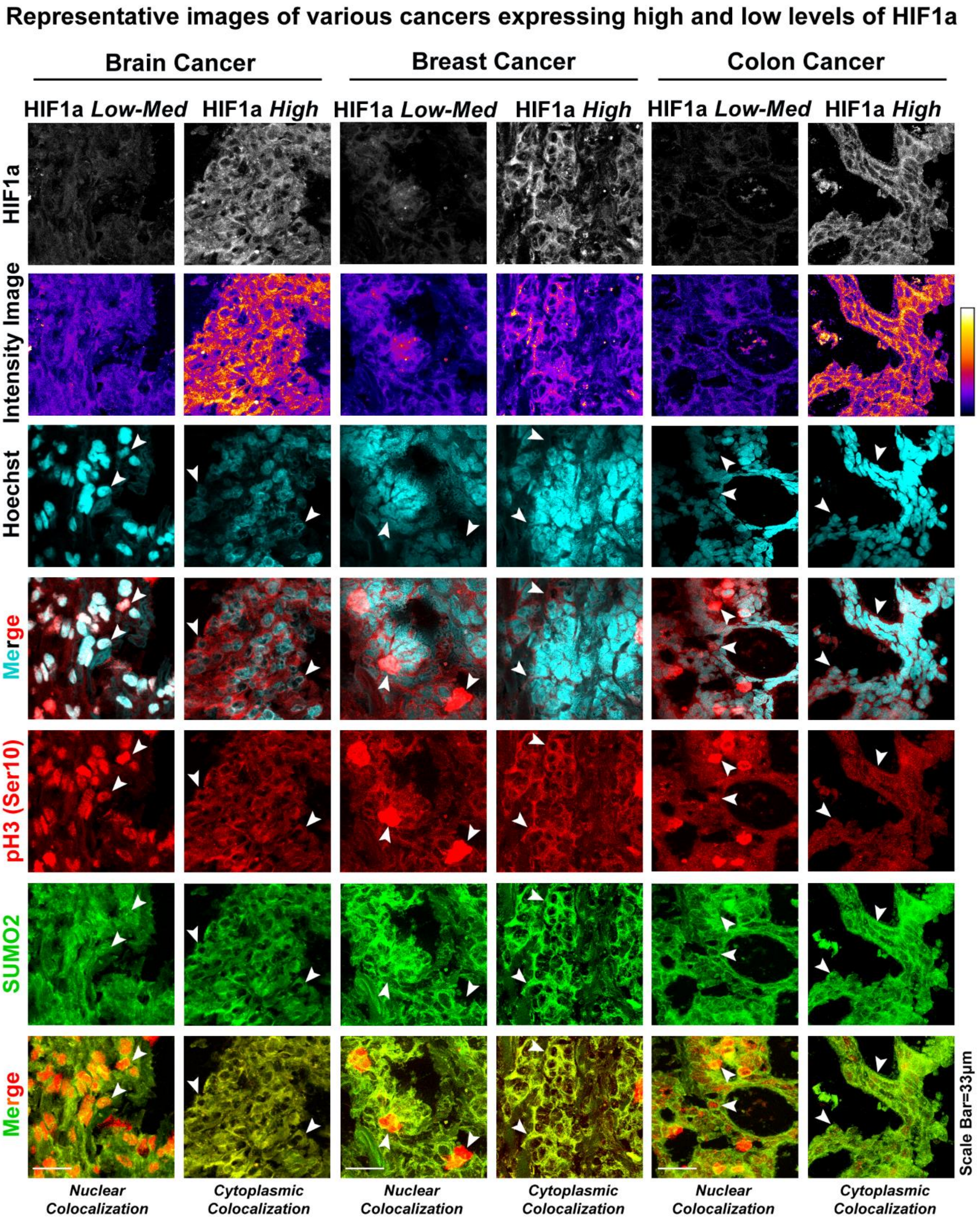

Supplementary Figure S14: Cytoplasmic colocalization of pH3(Ser10) and SUMO2 is observed in HIF1a high regions of brain, breast and colon cancer patient tissues.

Sections from brain, breast and colon cancer showed hypoxic (HIF1a high) and normoxic (HIF1a low-medium) areas within the same tissue. Cytoplasmic localization of pH3(Ser10) and SUMO2 was observed with the HIF1a antibody. On the contrary, HIF1a low to medium expressing regions showed appreciable nuclear colocalization. White arrowheads indicate the

single cells with nuclear or cytoplasmic colocalization of pH3(Ser10) and SUMO2. Image acquisition parameters for each channel were kept the same across each condition.

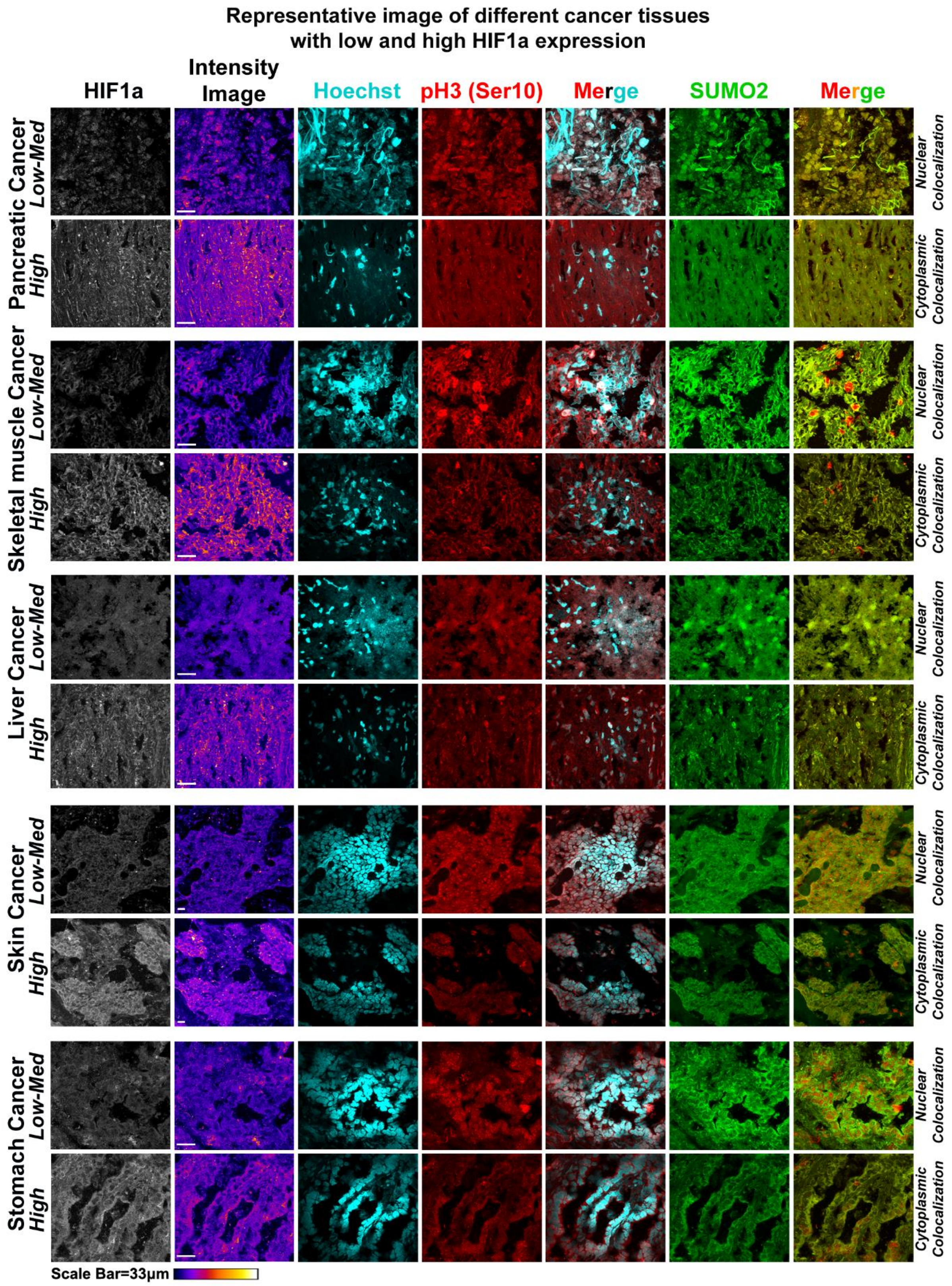

Supplementary Figure S15: Cytoplasmic colocalization of pH3(Ser10) and SUMO2 is observed in HIF1a high regions of pancreatic, skeletal muscle, liver, skin and stomach cancer patient tissues.

Sections from pancreatic, skeletal muscle, liver, skin and stomach cancer showed hypoxic (HIF1a high) and normoxic (HIF1a low-medium) areas within the same tissue. Cytoplasmic localization of pH3(Ser10) and SUMO2 was observed with the HIF1a antibody. On the contrary, HIF1a low to medium expressing regions showed appreciable nuclear colocalization. White arrowheads indicate the single cells with nuclear or cytoplasmic colocalization of pH3(Ser10) and SUMO2. Image acquisition parameters for each channel were kept the same across each condition.

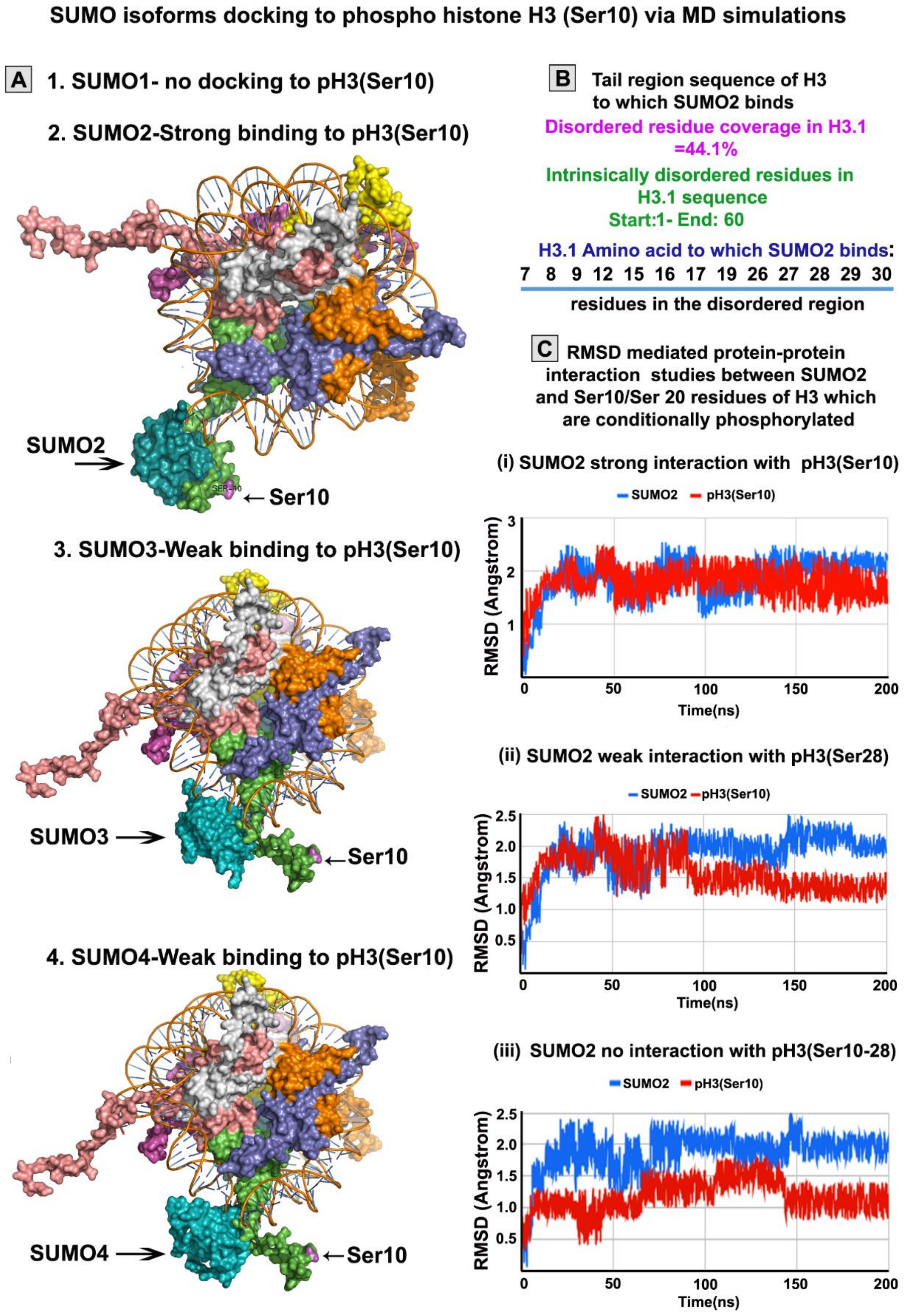

Supplementary Figure S16: Molecular dynamics simulations predict a stable conjugation of SUMO2 to pH3(Ser10) in the disordered tail region of the protein.

(A) The *in silico* predictions, via molecular dynamics simulations, indicated that among the four SUMO isoforms, only SUMO2 could conjugate with high fidelity with pH3(Ser10)

protein. **(B)** SUMO2 binding was observed in the tail region sequence of histone H3 protein. **(C)** Stable SUMO2 conjugation, over 200ns time frames, was observed with histone H3, phosphorylated explicitly at Ser10 residue and not Ser28 residue.

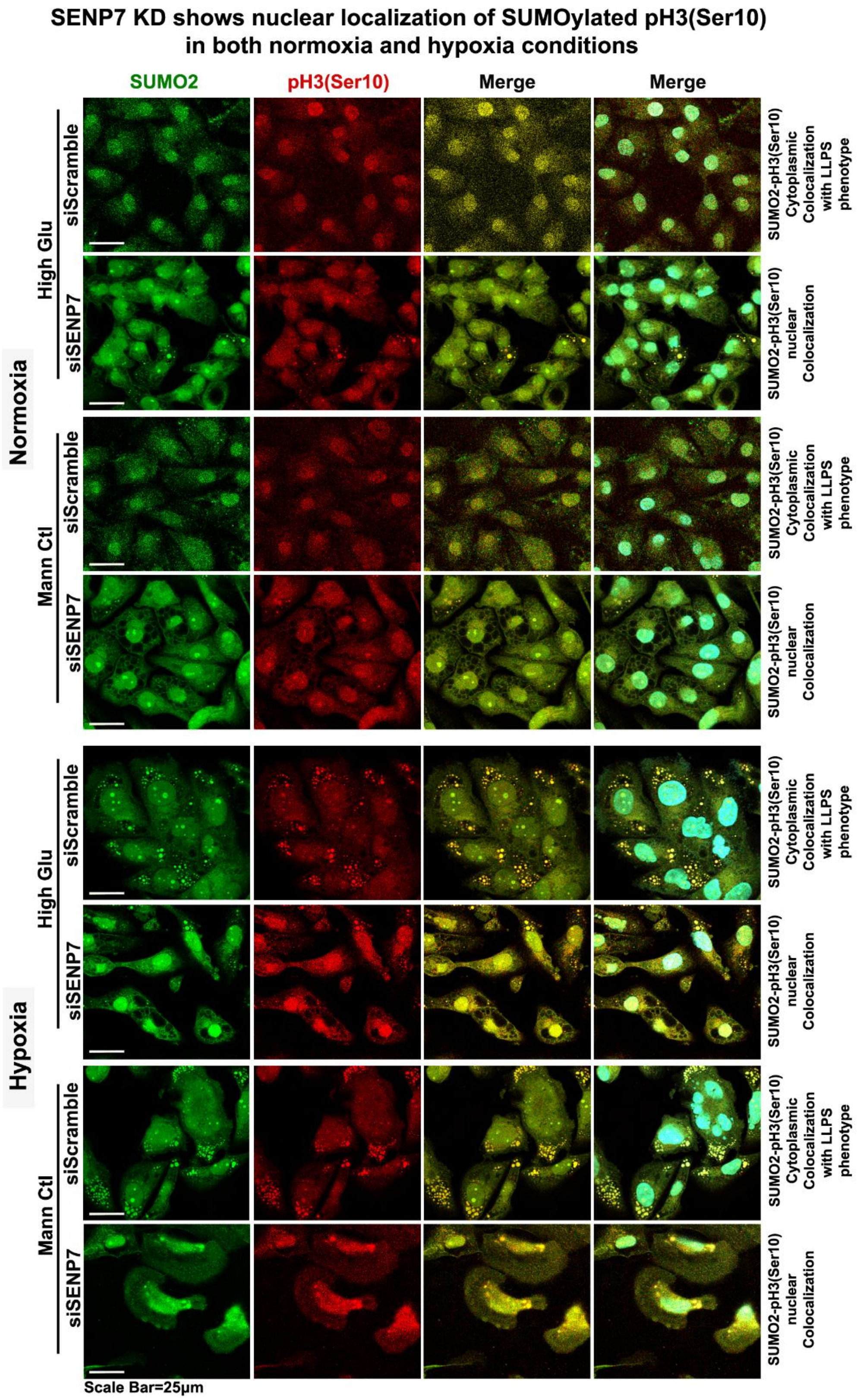

Supplementary Figure S17: siRNA-mediated knockdown of SENP7 resulted in increased nuclear localization of pH3(Ser10) in tumour cells exposed to hypoxic conditions.

CaSki cervical cancer cells were cultured in the general experimental set-up for 96 hours. Confocal images showed an increase in nuclear localization pH3(Ser10) upon siRNA-mediated downregulation of the sentrin protease, SENP7, even when acute hypoxia was present in the tumour microenvironment. Image acquisition parameters for each channel were kept the same across each condition and over independent replicates.

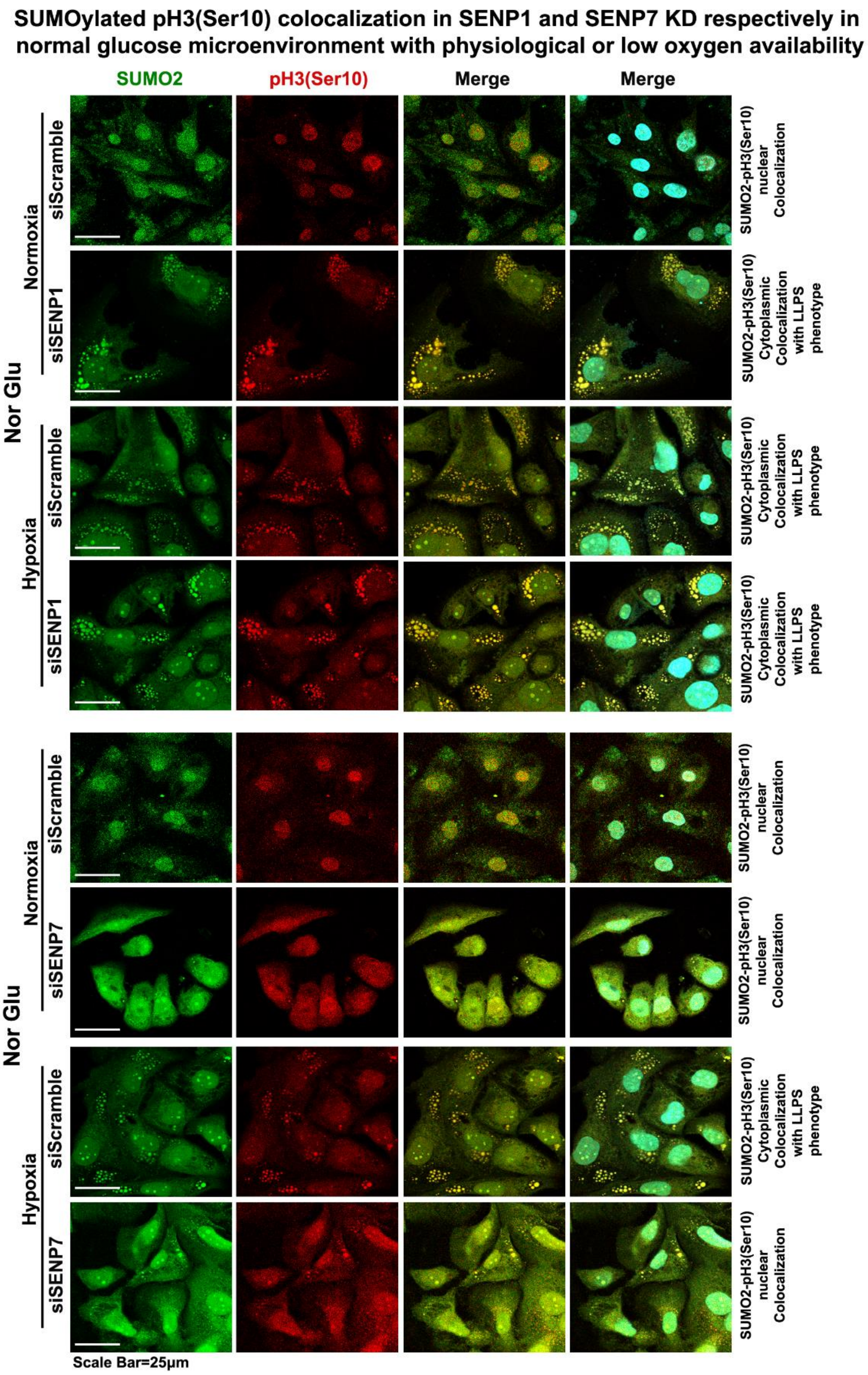

Supplementary Figure S18: siRNA-mediated knockdown of SENP1 resulted in decreased nuclear localization of pH3(Ser10) in normoxic normal glucose conditions, whereas the reverse was observed with SENP7 knockdown in tumour cells.

CaSki cervical cancer cells were cultured in the general experimental set-up for 96 hours. Confocal images showed that under normal glucose normoxia conditions, SENP1

downregulated tumour cells showed cytoplasmic sequestration and LLPS formation. Further, SENP1 downregulated tumour cells in NG hypoxia conditions had significantly higher cytoplasmic sequestration and LLPS formation. SENP7 downregulated tumour cells under NG normoxia conditions showed better nuclear localization than scrambled controls. SENP7 downregulated tumour cells in NG hypoxia condition also showed loss of LLPS phenotype and gained in nuclear localization vs scrambled controls. Image acquisition parameters for each channel were kept the same across each condition and over independent replicates.

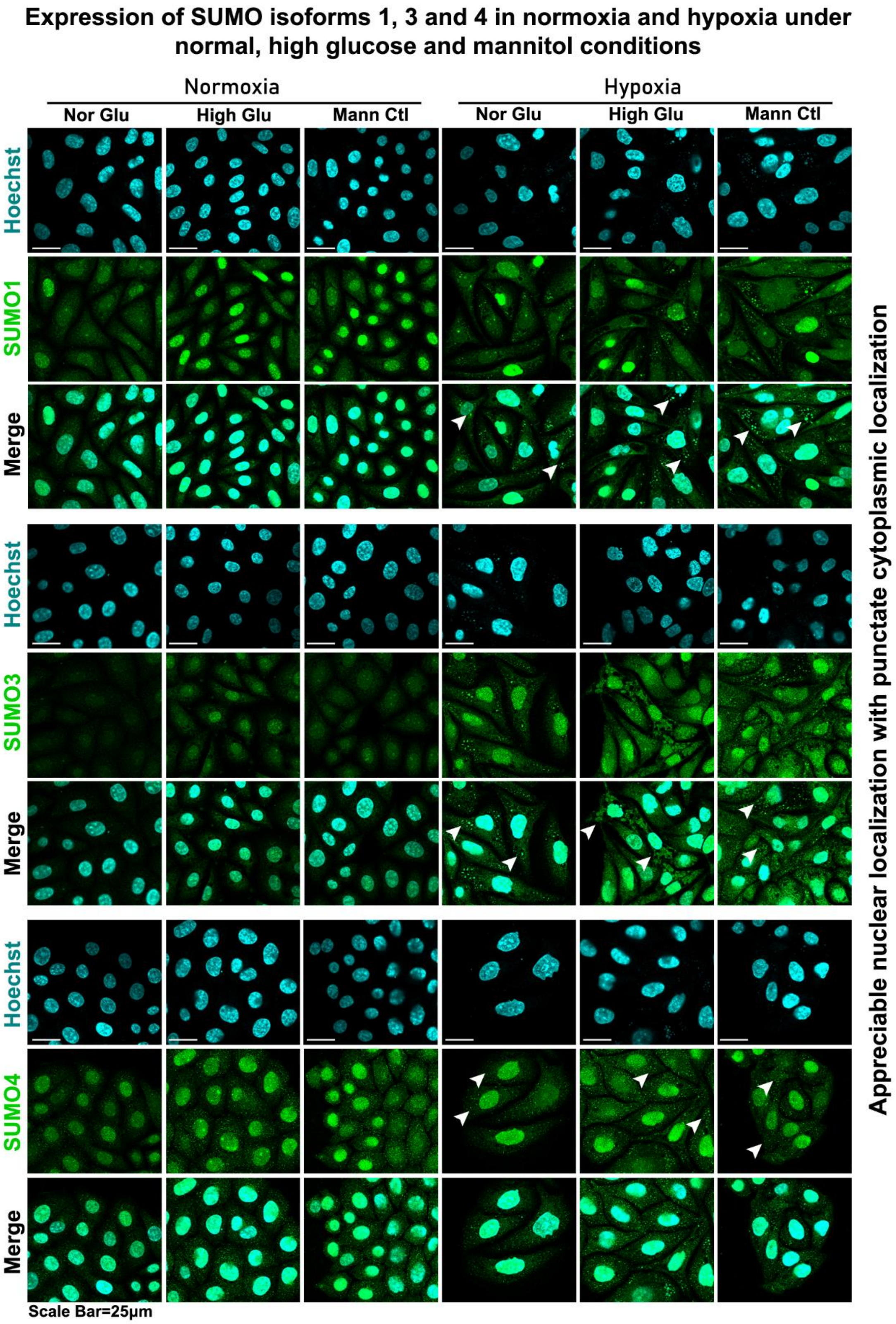

Supplementary Figure S19: SUMO isoforms 1, 3, and 4 can localize to tumour cells' nuclei in both hypoxic and normoxic conditions.

CaSki cervical cancer cells were cultured in the general experimental set-up for 96 hours. Confocal images showed that SUMO isoforms 1,3, and 4 could efficiently localize to the

nuclei across the treatment conditions in both normoxic and hypoxic microenvironments. However, some punctate signals were observed in the cytoplasm. Image acquisition parameters for each channel were kept the same across each condition and over independent replicates.

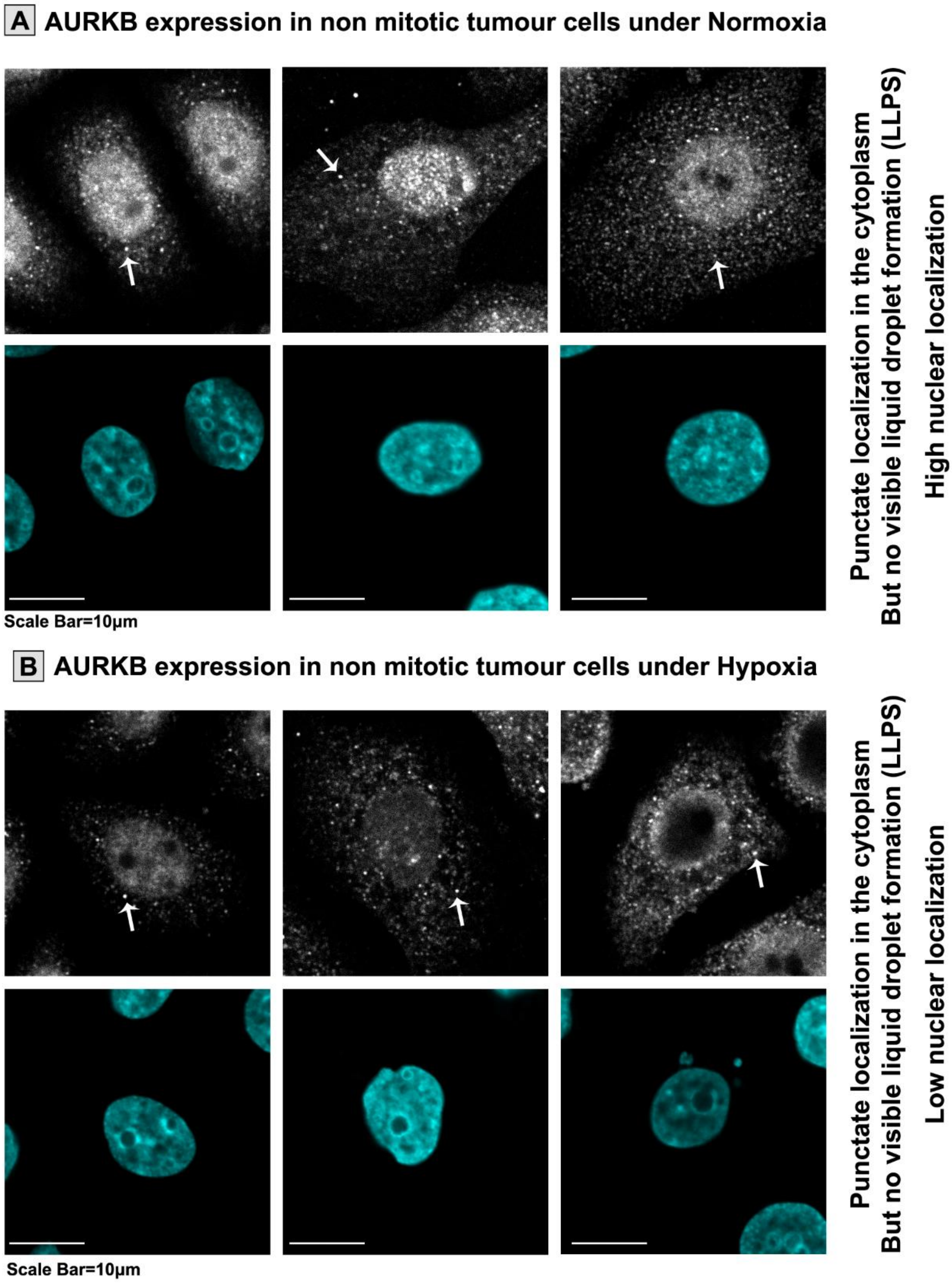

Supplementary Figure S20: The prominent nuclear localization of AURKB observed in normoxia was abrogated in the acute hypoxic conditions, but no LLPS phenotype was observed.

CaSki cervical cancer cells were cultured in the general experimental set-up for 96 hours. Confocal images showed prominent nuclear localization of AURKB protein in normoxic conditions. In contrast, in hypoxic conditions, there is a reduction in nuclear levels of

AURKB, which showed punctate signals in the cytoplasm. White arrows indicate nuclear localization of AURKB in normoxic conditions and punctate cytoplasmic localization in acute hypoxic conditions. Of note is that even in normoxia, AURKB bears a punctate cytoplasmic organization. Image acquisition parameters for each channel were kept the same across each condition and over independent replicates.

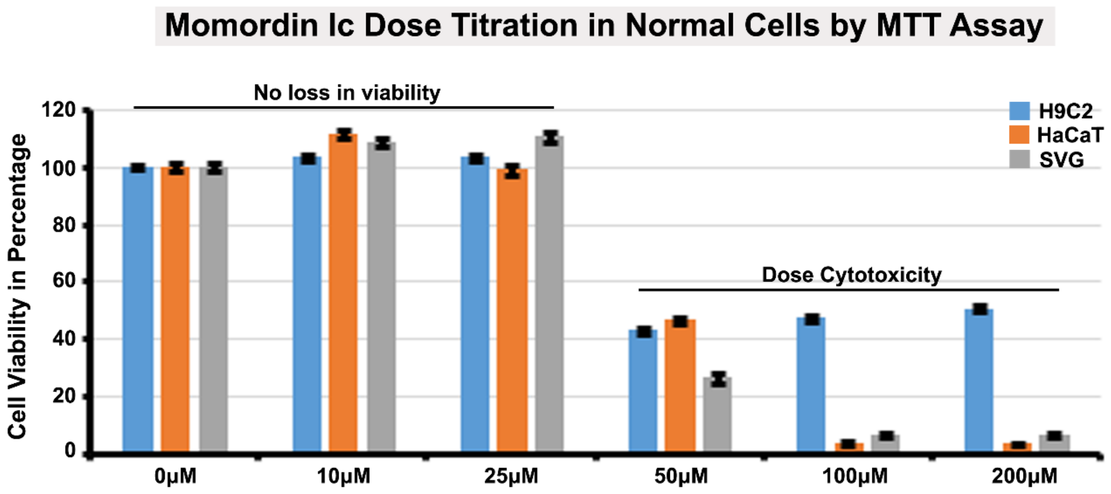

Supplementary Figure S21: Momordin Ic dose titration by MTT assay showed that the normal cells were unaffected up to a concentration of 25µM.

Normal cells: H9C2 (cardiomyoblasts), HaCaT (keratinocytes) and SVG (astrocytes) were treated with various concentrations of Momordin Ic for 24 hours, and an MTT-based viability assay was performed. MTT assay analysis showed that the viability of normal cells was unaffected by Momordin Ic till the concentration of 25 µM. Statistical significance is represented by p values: $*p \leq 0.05$, $**p \leq 0.01$ and $***p \leq 0.001$.

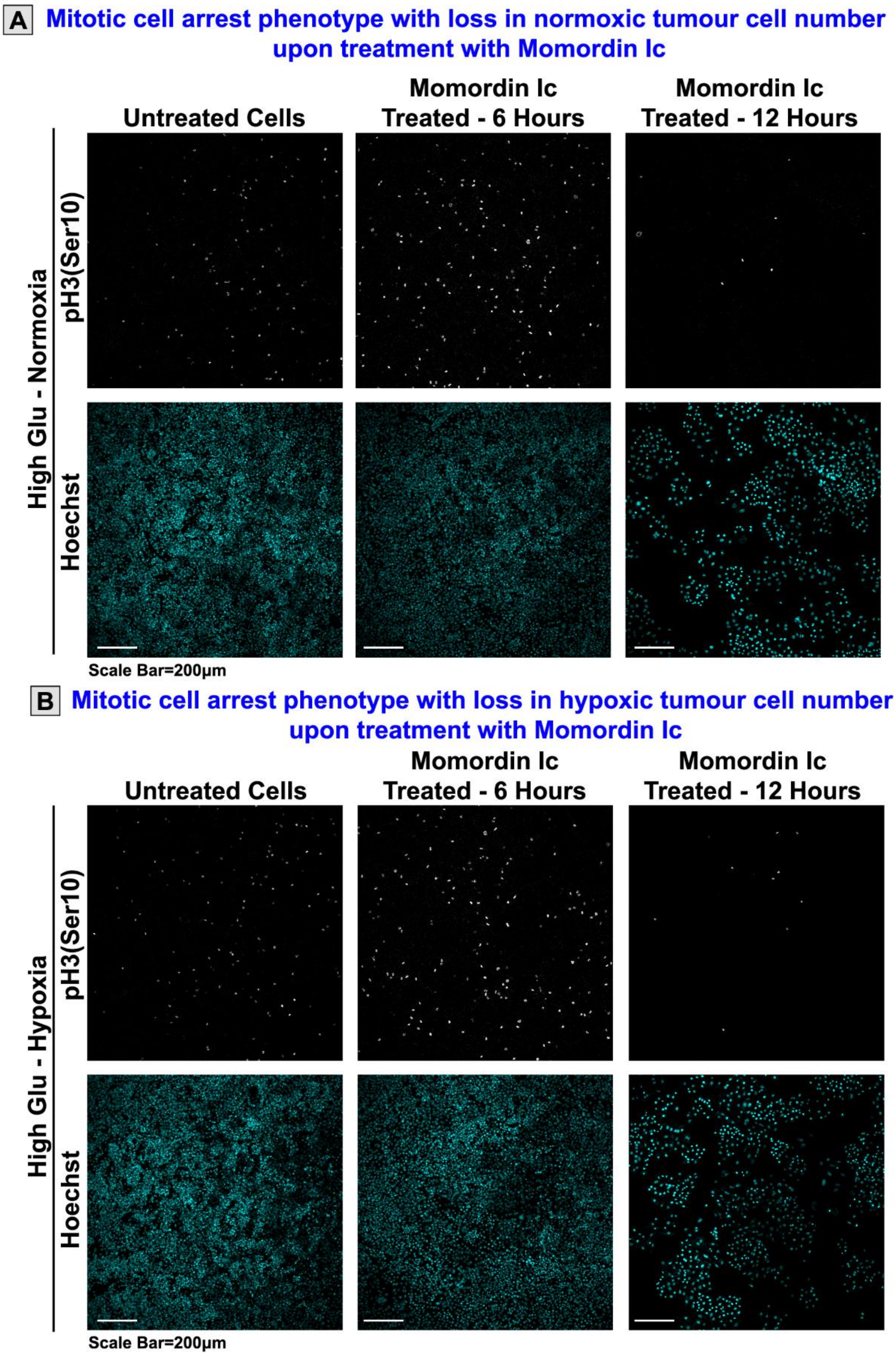

Supplementary Figure S22: Under both hypoxia and normoxia, high glucose conditions showed mitotic cell cycle arrest, in tumour cells, upon treatment with Momordin Ic.

CaSki cervical cancer cells were cultured in the general experimental set-up for 96 hours. Mitotic cell cycle arrest was observed within 6 hours of treatment with Momordin Ic in the

normoxic and hypoxic regions, with a noticeable reduction in tumour cell numbers occurring from 12 hours onwards, even in the presence of HG conditions. **(A)** Six hours of treatment with MC1 resulted in a steep increase in mitotic cells, although the cell count was seemingly unaffected; by 12 hours of treatment, the cell numbers started reducing in both normoxic and **(B)** hypoxic conditions in the presence of HG microenvironment. Although, the reduction in cell count in treated vs untreated conditions was less dramatic than in normoxia. Image acquisition parameters for each channel were kept the same across each condition and over independent replicates.

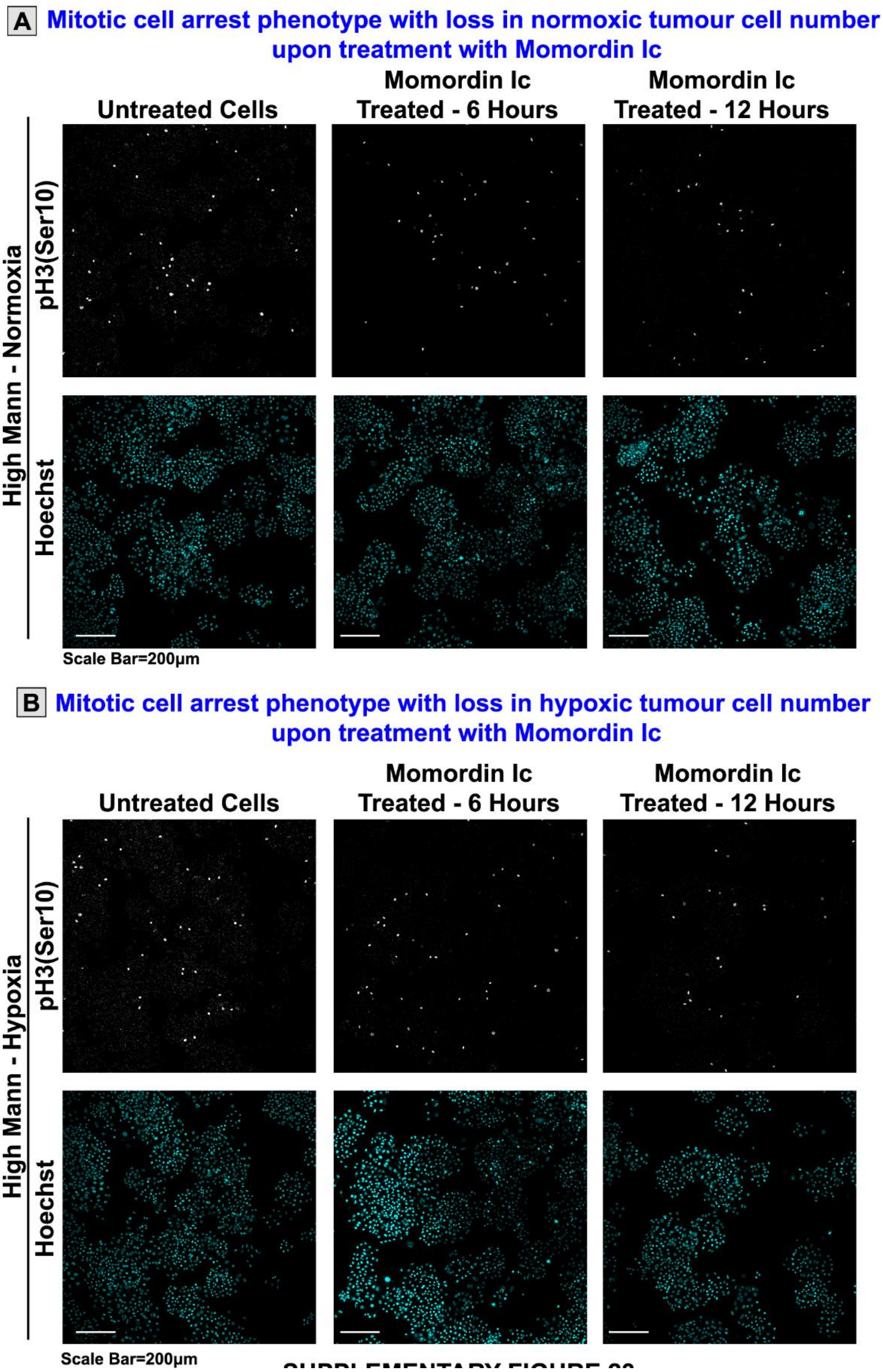

Supplementary Figure S23: Under both hypoxia and normoxia, high glucose hyperosmotic stress conditions showed mitotic cell cycle arrest in tumour cells upon treatment with Momordin Ic.

CaSki cervical cancer cells were cultured in the general experimental set-up for 96 hours. Mitotic cell cycle arrest was observed within 6 hours of treatment with Momordin Ic in the normoxic and hypoxic regions, with a noticeable reduction in cell number occurring from 12 hours onwards, even in the presence of HG-HO condition. **(A)** Six hours of treatment with MC1 resulted in a steep increase in mitotic cells: however, the cell count was seemingly unaffected. Nevertheless, by 12 hours of treatment, the cell number started reducing in both normoxic and **(B)** hypoxic conditions in the HG-HO niche. Image acquisition parameters for each channel were kept the same across each condition and over independent replicates.

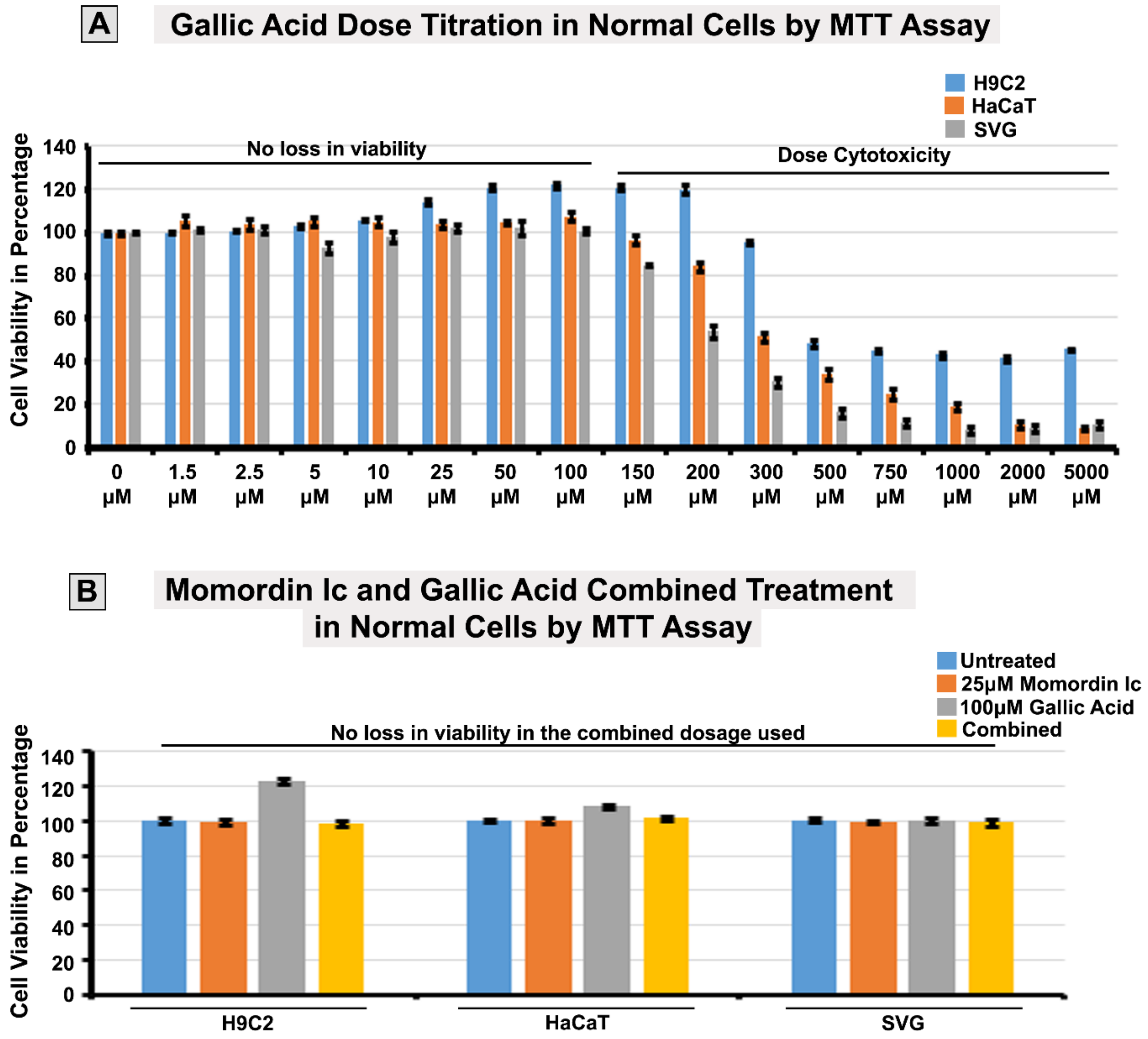

Supplementary Figure S24: MTT assay showed the viability of normal cells was not affected by the individual or combined doses of Momordin Ic and Gallic Acid drug treatments in this investigation.

A) Normal cells: H9C2 (cardiomyoblasts), HaCaT (keratinocytes) and SVG (astrocytes) were treated with various concentrations of gallic acid for 24 hours, and MTT-based viability assay was performed. MTT assay analysis showed that the viability of normal cells was unaffected by Gallic acid till the concentration of 100 µM. **B)** Normal cells: H9C2, HaCaT and SVG cells were treated with 25 µM Momordin Ic or 100 µM Gallic acid or combined for 24 hours. MTT-based cell viability assay showed that the viability of these normal cells was unaffected by the individual or in combination treatments. Statistical significance is represented by p values: $*p \leq 0.05$, $**p \leq 0.01$ and $***p \leq 0.001$.
